# Gut microbiota as a potential key to modulating humoral immunogenicity of new platform COVID-19 vaccines

**DOI:** 10.1038/s41392-023-01445-0

**Published:** 2023-05-03

**Authors:** Hye Seong, Bo Kyu Choi, Young-Hee Han, Jun Hyoung Kim, Jeong-An Gim, Sooyeon Lim, Ji Yun Noh, Hee Jin Cheong, Woo Joo Kim, Joon Young Song

**Affiliations:** 1 Department of Internal Medicine, Korea University College of Medicine, Seoul, Republic of Korea; 2 Asia Pacific Influenza Institute, Korea University College of Medicine, Seoul, Republic of Korea; 3 Vaccine Innovation Center, Korea University College of Medicine, Seoul, Republic of Korea; 4 Department of Biomedical Systems Informatics, Yonsei University College of Medicine, Seoul, Republic of Korea; 5 Department of Food and Nutrition, Chungbuk National University, Cheongju, Republic of Korea; 6 Department of Internal Medicine, Chungbuk National University Hospital, Cheongju, Republic of Korea; 7 Medical Science Research Center, Korea University College of Medicine, Seoul, Republic of Korea

**Keywords:** Vaccines, Vaccines, Predictive markers, Infectious diseases, Predictive markers

**Dear Editor**,

Coronavirus disease 2019 (COVID-19) is a novel respiratory infectious disease, caused by severe acute respiratory syndrome coronavirus 2 (SARS-CoV-2), which led to a global pandemic. Although vaccination is the best measure to overcome a pandemic, the immunogenicity of vaccines can be influenced by diverse factors, including intrinsic (age, sex, genetics, and comorbidities), extrinsic (diet, nutrition, and behavior), and vaccine-associated characteristics.^1^ In addition, the microbiome may play an essential role in controlling the immune response to both oral and parenteral vaccines.^2^ A recent human microbiome intervention study with a trivalent influenza vaccine suggested that microbiota dysbiosis was associated with increased inflammation and decreased vaccine immune response.^3^ Antibiotic abuse, obesity, diabetes, and other individual factors could cause intestinal microbiota dysbiosis, which might affect vaccine immunogenicity. Contrary to the influenza vaccine, in the case of COVID-19 vaccines, it would be possible to better evaluate the influence of gut microbes on vaccine immunogenicity in the absence of pre-existing immunity. In this study, we investigated the serial correlations between the gut microbiota and serum SARS-CoV-2 antibody levels after vaccination and analyzed the potential effects of vaccine platform (adenovirus-vectored versus mRNA vaccines).

We conducted a prospective cohort study of healthy adult participants fully vaccinated with BNT162b2 (mRNA vaccine) and ChAdOx1 (adenovirus-vectored vaccine) COVID-19 vaccines and collected stool and blood samples prior to the administration of the first (V1) and second doses (V2) and three weeks after the administration of the second dose (V3) (Fig. 1a). Overall and vaccine platform-dependent baseline characteristics and antibody responses are presented in supplementary Tables 1, 2, and 3. Examination of gut microbiota alterations following vaccination revealed that the mean community richness and microbial diversity (alpha diversity) gradually decreased from V1 to V2 to V3 in the ChAdOx1-vaccinated group (Fig. 1b and supplementary Fig. 1). Analysis using the Wilcoxon signed-rank test showed significant differences in Shannon diversity between V1 and V2 (*p* = 0.008) and V1 and V3 (*p* = 0.004) in ChAdOx1 recipients. Similarly, principal coordinate analysis of Bray–Curtis distances (beta diversity) using permutational multivariate analysis of variance indicated significantly distinct inter-set distances between the metagenome at V1 and that at V2 (*p* = 0.028) or V3 (*p* = 0.001) in ChAdOx1 recipients, but not in BNT162b2 recipients (Fig. 1b). Notable differences in changes in the distribution of bacterial taxonomic groups and their relative abundances at V1, V2, and V3 were also observed following ChAdOx1 vaccination compared to those observed following BNT162b2 vaccination (Fig. 1b and supplementary Fig. 2). Our results demonstrated that the two novel vaccine platforms differentially affected gut microbiota alteration. The interaction between the adenovirus vector and gut microbiome may have contributed to the platform-dependent differences in vaccine immunogenicity.

**Fig. 1:**
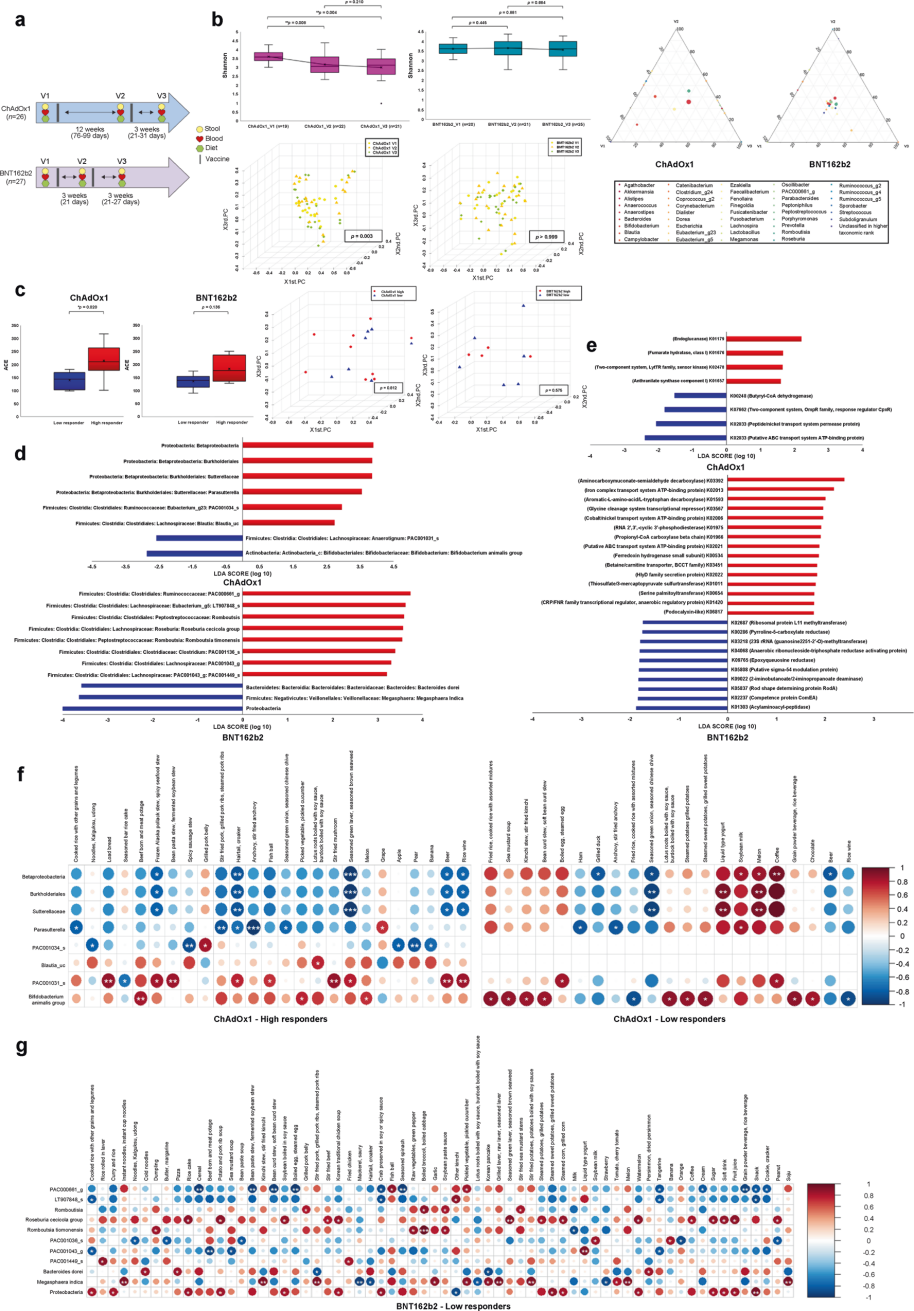
Serial associations between the gut microbiota, serum SARS-CoV-2 antibody levels and food intakes in adenovirus-vectored or mRNA vaccinees. **a** Schematic diagram of sample collection and survey. **b** Changes in alpha (Shannon) and beta diversity, and ternary plots following the administration of two different vaccine platforms. Changes in fecal microbiota diversity based on the Shannon index following ChAdOx1 and BNT162b2 vaccination. Beta diversity results assessed using principal coordinate analysis (PCoA) of Bray–Curtis distances at V1, V2, and V3 are shown for ChAdOx1 and BNT162b2 groups. Genus-level microbiota changes at V1, V2, and V3 are presented as ternary plots for ChAdOx1 and BNT162b2 groups. Each circle represents one genus, and the size of the circle reflects its relative abundance. **c** Baseline differences in the microbiome composition with respect to the immunogenicity of the vaccine platforms. Baseline differences in microbiota species richness (ACE) and inter-set distances with respect to the immunogenicity of the two different vaccine platforms. Comparison of high and low responders revealed a higher baseline ACE index. **d** Linear discriminant analysis effect size (LEfSe) analysis to identify taxonomic biomarkers. LEfSe was used to differentiate between high and low responders. The linear discrimination analysis scores revealed significant differences in microbiota composition according to vaccine immunogenicity in ChAdOx1- and BNT162b2-vaccinated groups. Only taxa with *p* < 0.05 are presented. **e** Linear discriminant analysis effect size analysis to identify functional biomarkers between high and low responders. Kyoto Encyclopedia of Genes and Genomes (KEGG) orthologs abundant in ChAdOx1- and BNT162b2-vaccinated groups are presented. Only orthologs with *p* < 0.05 are presented. **f**, **g** Taxonomy markers and significant items among 112 food items. Spearman rank analysis was conducted to evaluate the association between immunogenicity-related taxonomic biomarkers and 112 listed food items in **f** ChAdOx1 and **g** BNT162b2 recipients, respectively. The color gradients indicate the degree of correlation from red (positive correlation) to blue (negative correlation). *p < 0.05; **p = 0.01–0.001; ***p < 0.001

Next, to evaluate the role of the human gut microbiota in the humoral immune response to COVID-19 vaccines, the study participants were categorized as high or low responders according to their anti-SARS-CoV-2 S IgG titers at V3. Interestingly, we found that baseline microbial species richness (alpha diversity) was positively associated with humoral immunogenicity (Fig. 1c and supplementary Fig. 3). Subsequently, linear discriminant analysis effect size was used to determine and distinguish the composition of the gut microbiome based on immune responses in both ChAdOx1- and BNT162b2-vaccinated groups (Fig. 1d, supplementary Fig. 4, and supplementary Tables 4 and 5). In particular, a high abundance of the genus *Parasutterella* and the species *Eubacterium PAC001034_s* and *Blautia_uc* prior to vaccination was associated with high humoral immune responses in ChAdOx1 recipients. Bacteria of the genus *Parasutterella* produce succinate and enhance the abundance of tryptophan metabolites, which may exert potential beneficial effects on intestinal mucosal homeostasis by elevating hypoxanthine levels.^4^ Furthermore, *Parasutterella* has been suggested to play a potential role in the metabolism of cholesterol and maintenance of bile acids, especially secondary bile acids.^5^ Consistent with a previous finding that the reduction in secondary bile acid levels is associated with a diminished vaccine response,^3^ *Parasutterella* was noted as a crucial taxonomic biomarker for high immunogenicity in ChAdOx1 vaccine recipients. The species *Eubacterium PAC001034_s* and the genus *Blautia* belong to the families *Rumonococcaceae* and *Lachnospiraceae*, respectively, which are the primary producers of short-chain fatty acids that act as immunomodulatory metabolites. Moreover, similar to *Parasutterella, Blautia* also strengthens the intestinal barrier by inducing the expression of tight junction proteins and production of mucin by enterocytes and converting primary bile acids into secondary bile acids via 7-α-hydroxylation.^6^

In BNT162b2 recipients, a high abundance of the genera *Ruminococcaceae PAC000661_g*, *Romboutsia*, and *Lachnospiraceae PAC001043_g* and species *Clostridium PAC001136_s*, *Lachnospiraceae PAC001043_g PAC001449_s*, *Eubacterium LT907848_s*, *Romboutsia timonensis*, and *Roseburia cecicola* prior to vaccination was associated with a high immune response. The interaction of each microbiota may contribute to maintaining intestinal homeostasis and enhancing the vaccine immune response. *Clostridium PAC001136_s* was reported to be associated with mucosal healing.^7^ *Roseburia* species, a butyrate producer, is known to play a beneficial role in maintaining gut health and immune defense.^8^ *Lachnospiraceae* families might be associated with high immune response in both ChAdOx1 and BNT162b2 recipients owing to their ability to produce butyrate.

Based on taxonomic differences in each participant, we investigated the functional profiles that predicted vaccine response between the microbiota of the high and low-responder groups (Fig. 1e, supplementary Fig. 5, and supplementary Tables 6 and 7). Using the PICRUSt algorithm, a significant abundance of the module M00701 (multidrug resistance, efflux pump EmrAB) was observed in ChAdOx1 high responders, whereas the M00088 (ketone body biosynthesis) module and ko00380 (tryptophan metabolism) pathway were observed in BNT162b2 high responders. This may be related to the fact that the gut microbiome produces the butyrate metabolite 3-hydroxybutyrate and the essential amino acid tryptophan, which are involved in colonic homeostasis, mucosal integrity maintenance, and immunoregulation.^9^

Because the microbiota metabolizes food ingredients, diet is one of the significant determinants of microbiome composition and functional activity.^1^ The correlation analysis of taxonomic biomarkers with 112 food items, energy, and 13 nutrients was conducted before vaccination to examine the dietary link between the microbiome and humoral immune response (Fig. 1f, g and supplementary Figs. 6 and 7). We found that energy, carbohydrates, and sodium intakes were associated with a low abundance of the genus *Parasutterella*. Consistent with the findings of previous studies,^10^ high-fat consumption was negatively correlated with *Parasutterella* abundance in the present study. Consuming eggs and coffee resulted in a low abundance of *Anaerotignum PAC001031_s*, which exerts potentially beneficial effects on immunogenicity. Although riboflavin, niacin, and vitamin C intake may be beneficial owing to their positive correlation with bacteria enriched in high responders (the genus *Romboutsia* and the species *Eubacterium LT907848_s* and *Romboutsia timonensis*), these nutrients also correlated positively with bacteria enriched in low responders (*M. indica*). Because each nutrient can influence several bacteria differently, the effects of diet on microbiota and vaccine immunogenicity should be interpreted with caution.

There are several limitations in this study. First, the sample size was small, particularly because we excluded the intermediate responder group. However, we believe that clearer conclusion could be derived as we divide the participants into high or low responders, excepting intermediate responders (gray zone). Second, we could not evaluate the impact of microbiota among inactivated COVID-19 vaccine recipients because it was not avaialble during study periods. At the time of the study, SARS-CoV-2-naive adults were vaccinated against COVID-19 for the first time in their lives, but now more than 80% of the general population has experienced SARS-CoV-2 infection at least once. Moreover, bivalent COVID-19 vaccination is in progress. Thus, direct comparison might not be feasible with inactivated vaccine recipients in this study. In the previous study evaluating the correlation between gut microbiota composition and SARS-CoV-2 vaccines, *Bifidobacterium adolescentis* was persistently higher in subjects with high neutralizing antibodies to CoronaVac vaccine (inactivated COVID-19 vaccine), while BNT162b2 vaccinees showed a positive correlation with the total abundance of bacteria with flagella and fimbriae including *Roseburia faecis*. Further studies are warranted.

Collectively, this study revealed that the gut microbiome affects the immune response after COVID-19 vaccination and that the adenovirus vector induces changes in the microbiota that can affect the immune response after repeated vaccination. Moreover, we identified the specific taxonomic biomarkers, functional pathways, and potential nutrient factors that affect humoral immunogenicity. Further evaluation is warranted to validate the relevant biomarkers.

## Supplementary information

Supplementary information

## Data Availability

All data and materials are presented in the main manuscript or supplementary materials. Further data that support the findings of this study are available from the corresponding author upon request.
