# Supplementary material for: Gut microbiota as a potential key to modulating humoral immunogenicity of new platform COVID-19 vaccines
Source: Signal Transduct Target Ther. 2023 May 3;8:178. doi: 10.1038/s41392-023-01445-0 (PMC10154741; doi:10.1038/s41392-023-01445-0)

Supplementary Materials for

**Gut microbiota as a potential key to modulating humoral immunogenicity of new platform COVID-19 vaccines**

Hye Seong, Bo Kyu Choi, Young-Hee Han, Jun Hyoung Kim, Jeong-An Gim, Sooyeon Lim,

Ji Yun Noh, Hee Jin Cheong, Woo Joo Kim, and Joon Young Song

Correspondence to: infection@korea.ac.kr

**This PDF file includes:**

Materials and Methods

References

Tables S1 to S7

Figures S1 to S7

Materials and Methods

**Experimental model and participant details**

From February 25 to July 16, 2021, 53 healthy healthcare workers were prospectively recruited at a tertiary hospital in Seoul, Republic of Korea, and assigned to receive either BNT162b2 (n = 27) or ChAdOx1 (Oxford/AstraZeneca) (n = 26) vaccines. Participants who received medication that may have affected the gut microbiota—including antibiotics, laxatives, and motility drugs—in the month prior to vaccination, had a history of positive SARS-CoV-2 results after a nasopharyngeal PCR test, or tested positive for serum IgG against spike protein were excluded. No participant contracted COVID-19 during the study period as confirmed using nuclear capsid (N) protein antibody testing; anti-N antibody was measured using the SARS-CoV-2 IgG assay (Abbott Laboratories, Chicago, IL, USA) according to the manufacturer’s protocol.

Fecal and blood samples were collected serially at prior to the administration of the first (V1), second doses (V2), and three weeks after the administration of the second dose (V3). Patient data related to demographics, medications, probiotics, nutritional supplements, laboratory results, and dietary records were obtained by two trained physicians (Figure S1).

The study protocol was approved by the Institutional Review Board of the Korea University Guro Hospital (2021GR0097). Written informed consent was obtained from all participants. All procedures were performed per the ethical standards of relevant institutional and/or national research committees and in accordance with the 1964 Helsinki Declaration and its later amendments or comparable ethical standards.

**Sample collection and processing**

Fecal samples were collected using the Fecal Swab DNA Preservation & Transport Kits (Noble Bio, Hwaseong, South Korea) containing nucleic acid preservation media. Fecal samples in fecal swab transport medium were stored at –80 °C. Blood samples were obtained via venipuncture, collected in serum-separating tubes, and centrifuged at 2,500 rpm at –4 °C for 10 min; the serum-containing supernatant was pipetted into a clean plastic screw-cap vial and stored at –80 °C.

Immunoassay for quantitative determination of antibodies against the SARS-CoV-2 spike protein

Anti-S antibody titers were measured using the Elecsys® Anti-SARS-CoV-2 S assay kit (Roche, Rotkreuz, Switzerland), according to the manufacturer’s protocol. Titers below the lower limit of quantitation were set to 0.4 U/mL. Based on the data for immune correlation of protection and association between anti-S IgG and neutralizing antibody titers, participants with an IgG titer ≥ 2,500 U/mL or < 1,000 U/mL at V3 were considered high or low immune responders, respectively.^1-3^ In addition, participants with an IgG titer ≥ 120 U/mL or < 40 U/mL at V2 were considered V2 high responders or V2 low responders, respectively.

**Microbiological analysis**

*DNA extraction, PCR amplification, and sequencing*

Total DNA was extracted using the FastDNA® SPIN Kit for Soil (MP Biomedicals, Santa Ana, CA, USA), following the manufacturer’s instructions. PCR amplification was performed using fusion primers targeting the V3–V4 regions of the 16S rRNA gene, with the extracted DNA used as the template. For bacterial amplification, the following fusion primers were used: 341F (5ʹ-AATGATACGGCGACCACCGAGATCTACAC-XXXXXXXXTCGTCGGCAGCGTC-AGATGTGTATAAGAGACAG-CCTACGGGNGGCWGCAG-3ʹ; the underlined sequence indicates the target region primer) and 805R (5ʹ-CAAGCAGAAGACGGCATACGAGAT-XXXXXXXXGTCTCGTGGGCTCGG-AGATGTGTATAAGAGACAG-GACTACHVGGGTATCTAATCC-3ʹ). The fusion primers were constructed in the following order: P5 (P7) graft binding, i5 (i7) index, Nextera consensus, sequencing adaptor, and target region sequence. PCR amplification was carried out under the following conditions: initial denaturation at 95 °C for 3 min followed by 25 cycles of denaturation at 95 °C for 30 s, primer annealing at 55 °C for 30 s, and extension at 72 °C for 30 s, with a final elongation step at 72 °C for 5 min. The PCR products were confirmed by electrophoresis using 1% agarose gel followed by visualization using a Gel Doc system (Bio-Rad, Hercules, CA, USA). The amplified products were purified using the CleanPCR kit (CleanNA, Waddinxveen, Netherlands). Equal concentrations of the purified products were pooled, and short fragments (non-target products) were removed using the CleanPCR kit (CleanNA). Quality and product size were assessed on a Bioanalyzer 2100 system (Agilent, Palo Alto, CA, USA) using a DNA 7500 chip. Mixed amplicons were pooled, and the sequencing was performed at ChunLab, Inc. (Seoul, South Korea) using the Illumina MiSeq Sequencing System (Illumina, San Diego, CA, USA) according to the manufacturer’s instructions.

*DNA analysis pipeline*

The raw reads were quality-checked; low quality (<Q25) reads were filtered out using Trimmomatic ver.0.32. After quality control processing, paired-end sequence data were merged using the fastq_mergepairs command of VSEARCH ver.2.13.4 with default parameters. Next, the primers were trimmed using the Myers–Miller alignment algorithm^4^ at a similarity cut-off of 0.8. Non-specific amplicons, such as those that did not encode 16S rRNA, were detected using the nhmmer algorithm^5^ in the HMMER software package ver.3.2.1 with hmm profiles. Unique reads were extracted, and redundant reads were clustered with the unique reads using the deep-full-length command in VSEARCH.^6^ The EzBioCloud 16S rRNA database^7^ was used for taxonomic assignment using the usearch_global command of VSEARCH,^6^ followed by a more precise pairwise alignment.^4^ Chimeric reads were filtered to obtain reads with <97% similarity by reference-based chimeric read detection using the UCHIME algorithm^8^ and the non-chimeric 16S rRNA database from EzBioCloud. After chimeric filtering, reads that were not identified to the species level (with <97% similarity) in the EzBioCloud database were compiled, and the cluster_fast command^6^ was used for de novo clustering to generate additional operational taxonomic units (OTUs). OTUs with single reads (singletons) were omitted from further analysis. Secondary analyses, including diversity calculation and biomarker discovery, were performed using in-house programs of ChunLab, Inc.

The alpha diversity indices (ACE,^9^ Chao1,^10^ Jackknife,^11^ Shannon,^12^ NPShannon,^13^ Simpson,^12^ and phylogenetic diversity^14^) were estimated as previously described. To visualize sample differences, beta diversity distances were calculated using Jensen–Shannon,^15^ Bray–Curtis,^16^ Generalized UniFrac,^17^ and Fast UniFrac^18^ algorithms as appropriate. Functional profiles were predicted using the PICRUSt^19^ and MinPath^20^ algorithms, whereas taxonomic and functional biomarkers were identified using statistical comparison algorithms (linear discriminant analysis effect size [LEfSe]).^21^ All microbiome count data were normalized relative to 1,000 read counts before further use. All analyses were performed using the EzBioCloud 16S-based MTP, which is the bioinformatics cloud platform of ChunLab, Inc. (https://www.ezbiocloud.net).

**Nutritional surveys**

*Dietary intake survey*

The dietary intake survey was conducted using a food frequency questionnaire (FFQ) developed by the Korea National Health and Nutrition Examination Survey.^22,23^ The FFQ includes 112 items divided into rice, noodles and dumplings, bread and rice cakes, soups and stews, soybeans, eggs, meat and fish, vegetables, seaweed and potatoes, milk and dairy products, fruits, beverages, snacks, and alcoholic beverages. The frequency range of the FFQ has nine categories (rarely, once a month, 2−3 times a month, once a week, 2−4 times a week, 5−6 times a week, once a day, twice a day, and thrice a day), and the portion size includes three categories (small, medium, and large) compared with the standard amount.

*Energy and nutrient intake calculation*

After measuring the daily intake of each item by frequency and portion size, the daily mean values for intake of energy and 13 nutrients (carbohydrate, protein, fat, calcium, phosphorous, iron, sodium, potassium, vitamin A, thiamin, riboflavin, niacin, and vitamin C) was calculated using the nutrient database for each item.^23^ Additionally, information related to the product name, intake frequency, and intake amount of dietary supplements was obtained, and the intake of vitamins and minerals was calculated. Finally, by combining the results of the dietary intake survey and dietary supplements, the final daily intake of energy and nutrients was calculated for each subject.

**Statistical analysis**

All continuous variables were expressed as the median ± interquartile range (IQR; third IQR–first IQR), and non-parametric tests were used to compare the differences between different groups. Categorical variables were presented as numbers (percentages). The Mann–Whitney *U* test was used to compare ChAdOx1- and BNT162b2-vaccinated groups, whereas the Wilcoxon signed-rank test was used for pairwise comparisons in the same vaccination group. For correlation analysis, the Spearman rank test was performed for the 112 food items, whereas the Pearson correlation test was performed to calculate the nutrient intake. Statistical analyses were performed using the R Statistical Software version 4.1.2 (R Foundation for Statistical Computing, Vienna, Austria).

References

1. Feng, S. et al. Correlates of protection against symptomatic and asymptomatic SARS-CoV-2 infection. *Nat Med.* **27**, 2032–2040 (2021).

2. Li, L. et al. Effect of convalescent plasma therapy on time to clinical improvement in patients with severe and life-threatening COVID-19: a Randomized clinical trial. *Jama.* **324**, 460–470 (2020).

3. Wajnberg, A. et al. Robust neutralizing antibodies to SARS-CoV-2 infection persist for months. *Science.* **370**, 1227–1230 (2020).

4. Myers, E. W. & Miller, W. Optimal alignments in linear space. *Bioinformatics.* **4**, 11–17 (1988).

5. Wheeler, T. J. & Eddy, S. R. nhmmer: DNA homology search with profile HMMs. *Bioinformatics.* **29**, 2487–2489 (2013).

6. Rognes, T., Flouri, T., Nichols, B., Quince, C. & Mahé, F. VSEARCH: a versatile open source tool for metagenomics. *PeerJ.* **4**, e2584 (2016).

7. Yoon, S. H. et al. Introducing EzBioCloud: a taxonomically united database of 16S rRNA gene sequences and whole-genome assemblies. *Int J Syst Evol Microbiol.* **67**, 1613 (2017).

8. Edgar, R. C., Haas, B. J., Clemente, J. C., Quince, C. & Knight, R. UCHIME improves sensitivity and speed of chimera detection. *Bioinformatics.* **27**, 2194–2200 (2011).

9. Chao, A. & Lee, S. M. Estimating the number of classes via sample coverage. *J Am Stat Assoc.* **87**, 210–217 (1992).

10. Chao, A. Estimating the population size for capture-recapture data with unequal catchability. *Biometrics.* **43**, 783-791 (1987).

11. Burnham, K. P. & Overton, W. S. Robust estimation of population size when capture probabilities vary among animals. *Ecology.* **60**, 927–936 (1979).

12. Magurran, A. E. Measuring biological diversity. (John Wiley & Sons, 2013).

13. Chao, A. & Shen, T. J. Nonparametric estimation of Shannon’s index of diversity when there are unseen species in sample. *Environ Ecol Stat.* **10**, 429–443 (2003).

14. Faith, D. P. Conservation evaluation and phylogenetic diversity. *Biol Conserv.* **61**, 1–10 (1992).

15. Lin, J. Divergence measures based on the Shannon entropy. *IEEE Trans Inf Theory.* **37**, 145–151 (1991).

16. Beals, E. W. Bray-Curtis ordination: an effective strategy for analysis of multivariate ecological data. *Adv Ecol Res.* **14**, 1–55 (1984).

17. Chen, J. et al. Associating microbiome composition with environmental covariates using generalized UniFrac distances. *Bioinformatics.* **28**, 2106–2113 (2012).

18. Hamady, M., Lozupone, C. & Knight, R. Fast UniFrac: facilitating high-throughput phylogenetic analyses of microbial communities including analysis of pyrosequencing and PhyloChip data. *ISME J.* **4**, 17–27 (2010).

19. Ye, Y. & Doak, T. G. A parsimony approach to biological pathway reconstruction/inference for genomes and metagenomes. *PLoS Comput Biol.* **5**, e1000465 (2009).

20. Langille, M. G. et al. Predictive functional profiling of microbial communities using 16S rRNA marker gene sequences. *Nat Biotechnol.* **31**, 814–821 (2013).

21. Segata, N. et al. Metagenomic biomarker discovery and explanation. *Genome Biol.* **12**, 1–18 (2011).

22. Yun, S. H., Shim, J. S., Kweon, S. & Oh, K. Development of a food frequency questionnaire for the Korea National Health and Nutrition Examination Survey: data from the fourth Korea National Health and Nutrition Examination Survey (KNHANES IV). *kjn.* **46**, 186-196 (2013).

23. Kim, D. W. et al. Reproducibility and validity of an FFQ developed for the Korea National Health and Nutrition Examination Survey (KNHANES). *Public Health Nutr*. **18**, 1369–1377 (2015),

| **Table S1. Baseline characteristics and antibody responses for all participants** | | | | | | | | |
| --- | --- | --- | --- | --- | --- | --- | --- | --- |
| **Characteristics** | **ChAdOx1** (*n* = 26) | | | ***p* value** | **BNT162b2** (*n* = 27) | | | ***p* value** |
|  | **V1** | **V2** | **V3** | **V1-2/V2-3/V1-3** | **V1** | **V2** | **V3** | **V1-2/V2-3/V1-3** |
| **Age (years)** |  | 37.0±24.0 |  | **S1** |  | 31.0±8.5 |  | **S1** |
| **Female sex (%)** |  | 20 (50.0) |  | **S2** |  | 20 (50.0) |  | **S2** |
| **BMI (kg/m^2^)** |  | 23.4±5.7 |  | **S3** |  | 20.9±4.9 |  | **S3** |
| **Anti-SARS-CoV-2 S IgG (U/mL)** | 0.4 | 73.4±84.5 | 1373±3320.5 | **S4, S5** | 0.4 | 70.3±162.7 | 1981±1423.0 | **S4, S5** |
| **Laboratory test results** |  |  |  |  |  |  |  |  |
| WBC (/uL) | 5150±1380 | 5900±1600 | 5500±2050 | 0.213/0.153/0.799 | 6200±1900 | 6100±1830 | 5800±2200 | 0.933/0.279/0.264 |
| ANC (/uL) | 2897±1190 | 3185±1338 | 3023±1281 | 0.191/0.328/0.909 | 3357±1732 | 3052±1354 | 2890±1344 | 0.068/0.701/0.031 |
| Hemoglobin (g/dL) | 13.3±1.4 | 13.4±1.2 | 13.4±1.6 | 0.054/0.352/0.130 | 13.2±2.0 | 13.3±1.4 | 13.3±2.4 | 0.750/0.077/0.145 |
| MCV (fL) | 92.1±6.7 | 92.0±7.7 | 92.1±7.3 | 0.387/0.756/0.241 | 90.9±4.0 | 90.8±3.5 | 90.6±4.2 | 0.782/0.149/0.525 |
| MCH (pg) | 30.3±2.4 | 30.9±2.4 | 31.0±2.9 | <0.001/0.977/<0.001 | 29.9±1.9 | 29.9±2.0 | 30.0±1.9 | 0.218/0.714/0.415 |
| MCHC (g/dL) | 32.8±0.8 | 33.5±0.6 | 33.5±0.8 | <0.001/0.388/<0.001 | 33.0±0.9 | 33.1±0.9 | 33.1±1.0 | 0.312/0.882/0.393 |
| Platelet count (10^3^/uL) | 271.5±97.8 | 255.5±70.5 | 267.0±88.5 | 0.747/0.128/0.310 | 266.0±65.0 | 297.0±114.0 | 269.0±82.0 | 0.133/0.746/0.009 |
| BUN (mg/dL) | 12.6±6.7 | 11.9±6.9 | 12.6±4.8 | 0.174/0.760/0.186 | 12.5±4.4 | 13.3±5.1 | 11.6±4.1 | 0.102/0.501/0.030 |
| Creatinine (mg/dL) | 0.60±0.15 | 0.65±0.18 | 0.65±0.16 | 0.047/0.387/0.004 | 0.64±0.13 | 0.60±0.10 | 0.61±0.10 | 0.085/0.263/0.273 |
| Albumin (g/dL) | 4.50±0.40 | 4.50±0.20 | 4.45±0.32 | 0.145/0.885/0.232 | 4.40±0.30 | 4.60±0.30 | 4.50±0.30 | <0.001/0.006/0.002 |
| HDL cholesterol (mg/dL) | 67.0±27.8 | 65.5±19.8 | 70.0±19.0 | 0.352/0.909/0.208 | 57.0±30.0 | 64.0±26.0 | 63.0±32.0 | 0.009/0.760/0.009 |
| LDL cholesterol (mg/dL) | 118.0±34.3 | 114.0±35.3 | 119.5±28.5 | 0.767/0.751/0.939 | 99.0±39.0 | 109.0±28.0 | 110.0±27.0 | 0.049/0.301/0.041 |
| Triglyceride (mg/dL) | 90.5±65.8 | 79.0±71.8 | 82.5±80.8 | 0.292/0.603/0.989 | 78.0±103.0 | 94.0±73.0 | 83.0±66.0 | 0.799/0.380/0.904 |
| AST (IU/L) | 21.5±6.5 | 20.5±7.8 | 21.0±8.3 | 0.444/0.353/0.527 | 22.0±11.0 | 24.0±14.0 | 26.0±15.0 | 0.014/0.450/0.005 |
| ALT (IU/L) | 17.5±9.5 | 14.0±9.3 | 15.0±10.5 | 0.526/0.029/0.787 | 15.0±17.0 | 17.0±16.0 | 15.0±26.0 | 0.177/0.538/0.123 |
| GGT (IU/L) | 16.0±12.0 | 16.0±10.3 | 15.5±12.3 | 0.313/0.252/0.942 | 15.0±18.0 | 15.0±13.0 | 14.0±16.0 | 0.167/0.530/0.052 |
| Total bilirubin (mg/dL) | 0.57±0.42 | 0.60±0.43 | 0.65±0.32 | 0.155/0.689/0.170 | 0.59±0.42 | 0.65±0.46 | 0.74±0.43 | 0.015/0.648/0.013 |
| Glucose (mg/dL) | 102.5±27.8 | 97.5±15.5 | 97.5±17.3 | 0.186/0.703/0.174 | 91.0±14.0 | 94.0±18.0 | 97.0±19.0 | 0.556/0.600/0.839 |
| CRP (mg/L) | 0.36±0.95 | 0.37±0.61 | 0.66±0.79 | 0.062/0.053/0.060 | 0.31±0.59 | 0.31±0.62 | 0.26±0.35 | 0.203/0.313/0.046 |
| **Underlying diseases** |  |  |  |  |  |  |  |  |
| Hypertension |  | 2 (50.0) |  | **S6** |  | 2 (50.0) |  | **S6** |
| Diabetes mellitus |  | 1 (50.0) |  | **S7** |  | 1 (50.0) |  | **S7** |
| Dyslipidemia |  | 1 (33.3) |  | **S8** |  | 2 (66.7) |  | **S8** |
| HBV carrier |  | 3 (100.0) |  | **S9** |  | 0 (0.0) |  | **S9** |
| **S1:** ChAdOx1 age vs. BNT162b2 age, *p* = 0.050; **S2:** ChAdOx1 sex vs. BNT162b2 sex, *p* >0.999; **S3:** ChAdOx1 BMI vs. BNT162b2 BMI, *p* = 0.200; **S4:** V2 ChAdOx1 Anti-SARS-CoV-2 S IgG vs. V2 BNT162b2 Anti-SARS-CoV-2 S IgG, *p* = 0.528; **S5:** V3 ChAdOx1 Anti-SARS-CoV-2 S IgG vs. V3 BNT162b2 Anti-SARS-CoV-2 S IgG, *p* = 0.581; **S6:** ChAdOx1 vs. BNT162b2 hypertension, *p* >0.999; **S7:** ChAdOx1 vs. BNT162b2 diabetes mellitus, *p* >0.999; **S8:** ChAdOx1 vs. BNT162b2 dyslipidemia, *p* >0.999; **S9:** ChAdOx1 vs. BNT162b2 HBV carrier, *p* = 0.111.  BMI, body mass index; WBC, white blood cell; ANC, absolute neutrophil count; MCV, mean corpuscular volume; MCH, mean corpuscular hemoglobin; MCHC, mean corpuscular hemoglobin concentration; BUN, blood urea nitrogen; HDL, high density lipoprotein; LDL, low density lipoprotein; AST, aspartate transaminase; ALT, alanine transaminase; GGT, gamma-glutamyl transferase; CRP, C-reactive protein; HBV, hepatitis B.  Continuous variables are shown as median ± interquartile range (IQR) and categorical variables as numbers (percentage). | | | | | | | | |

| **Table S2. Comparison of baseline characteristics according to the antibody responses in ChAdOx1 group** | | | | | | | | |
| --- | --- | --- | --- | --- | --- | --- | --- | --- |
| **Characteristics** | **High responders** (*n* = 9) | | |  | **Low responders** (*n* = 8) | | | ***p* value** |
|  | **V1** | **V2** | **V3** |  | **V1** | **V2** | **V3** | **GV1-PV1/GV2-PV2/GV3-PV3** |
| **Age (years)** |  | 40.0±22.0 |  |  |  | 34.5±16.0 |  | 0.289 |
| **Female sex (%)** |  | 7 (50.0) |  |  |  | 7 (50.0) |  | > 0.999 |
| **BMI (kg/m^2^)** |  | 24.1±6.5 |  |  |  | 20.9±4.4 |  | 0.211 |
| **Anti-SARS-CoV-2 S IgG (U/mL)** | 0.4 | 69.4±100.6 | 4278.0±2663.0 |  | 0.4 | 58.9±84.2 | 522.0±496.0 | >0.999/0.773/0.001 |
| **Laboratory test results** |  |  |  |  |  |  |  |  |
| WBC (cells/uL) | 5200±1700 | 6200±2300 | 5100±2500 |  | 4750±2000 | 4950±2750 | 5150±2850 | 0.630/0.248/0.923 |
| ANC (cells/uL) | 2922±1539 | 3788±1381 | 3244±1942 |  | 2746±1624 | 2816±1901 | 3081±1332 | 0.847/0.178/0.700 |
| Hemoglobin (g/dL) | 13.3±1.8 | 13.6±1.1 | 13.5±2.2 |  | 13.4±0.9 | 13.6±0.9 | 13.4±1.6 | 0.499/0.809/0.772 |
| MCV (fL) | 89.9±6.7 | 89.2±7.0 | 88.7±6.0 |  | 93.8±4.6 | 93.0±3.2 | 93.1±4.0 | 0.248/0.248/0.163 |
| MCH (pg) | 29.5±2.6 | 29.7±2.8 | 29.8±2.8 |  | 30.7±1.2 | 31.5±1.1 | 31.5±1.9 | 0.194/0.112/0.163 |
| MCHC (g/dL) | 32.6±0.9 | 33.3±0.7 | 33.5±0.8 |  | 32.9±0.6 | 33.8±0.4 | 33.9±0.7 | 0.885/0.052/0.438 |
| Platelet count (10^3^/uL) | 221.0±104.0 | 252.0±107.5 | 243.0±115.0 |  | 291.5±80.3 | 266.5±74.5 | 274.0±73.5 | 0.211/0.810/0.630 |
| BUN (mg/dL) | 12.7±5.9 | 11.3±3.3 | 12.5±3.7 |  | 9.6±4.5 | 11.4±4.4 | 10.7±4.1 | 0.083/0.700/0.386 |
| Creatinine (mg/dL) | 0.60±0.24 | 0.64±0.25 | 0.63±0.26 |  | 0.64±0.11 | 0.65±0.14 | 0.65±0.11 | 0.735/0.736/>0.999 |
| Albumin (g/dL) | 4.40±0.40 | 4.50±0.30 | 4.40±0.45 |  | 4.60±0.35 | 4.50±0.17 | 4.45±0.20 | 0.355/0.882/0.845 |
| HDL cholesterol (mg/dL) | 61.0±23.5 | 64.0±26.0 | 63.0±24.5 |  | 69.0±20.0 | 72.5±19.3 | 71.0±18.8 | 0.082/0.290/0.248 |
| LDL cholesterol (mg/dL) | 117.0±50.0 | 135.0±66.5 | 121.0±69.0 |  | 120.0±30.0 | 109.0±22.8 | 111.0±22.0 | 0.923/0.441/0.441 |
| Triglyceride (mg/dL) | 111.0±59.0 | 100.0±145.5 | 90.0±163.5 |  | 71.0±79.3 | 63.0±60.0 | 74.0±64.8 | 0.178/0.075/0.810 |
| AST (IU/L) | 22.0±8.0 | 23.0±11.0 | 23.0±11.5 |  | 19.5±4.5 | 19.5±8.5 | 18.5±4.3 | 0.228/0.500/0.161 |
| ALT (IU/L) | 19.0±10.0 | 19.0±7.0 | 22.0±9.5 |  | 11.0±10.8 | 11.5±7.3 | 12.0±7.5 | 0.067/0.130/0.026 |
| GGT (IU/L) | 22.0±11.5 | 18.0±8.5 | 17.0±13.5 |  | 12.5±13.0 | 11.5±6.3 | 10.5±9.5 | 0.360/0.074/0.111 |
| Total bilirubin (mg/dL) | 0.49±0.43 | 0.56±0.43 | 0.72±0.26 |  | 0.73±0.49 | 0.68±0.36 | 0.66±0.38 | 0.335/0.335/0.885 |
| Glucose (mg/dL) | 106.0±35.0 | 95.0±16.5 | 109.0±26.0 |  | 99.5±22.5 | 97.5±27.5 | 93.5±13.8 | 0.773/0.885/0.027 |
| CRP (mg/L) | 0.29±1.57 | 0.33±2.30 | 0.64±2.27 |  | 0.37±0.35 | 0.35±0.48 | 0.63±0.63 | 0.470/0.193/0.441 |
| **Underlying diseases** |  |  |  |  |  |  |  |  |
| Hypertension |  | 2 (100.0) |  |  |  | 0 (0.0) |  | 0.471 |
| Diabetes mellitus |  | 0 (0.0) |  |  |  | 1 (100.0) |  | 0.471 |
| Dyslipidemia |  | 1 (100.0) |  |  |  | 0 (0.0) |  | > 0.999 |
| HBV carrier |  | 2 (66.7) |  |  |  | 1 (33.3) |  | > 0.999 |
| **GV1**, high responders V1; **GV2**, high responders V2; **GV3**, high responders V3; **PV1**, low responders V1; **PV2**, low responders V2; **PV3**, low responders V3.  BMI, body mass index; WBC, white blood cell; ANC, absolute neutrophil count; MCV, mean corpuscular volume; MCH, mean corpuscular hemoglobin; MCHC, mean corpuscular hemoglobin concentration; BUN, blood urea nitrogen; HDL, high density lipoprotein; LDL, low density lipoprotein; AST, aspartate transaminase; ALT, alanine transaminase; GGT, gamma-glutamyl transferase; CRP, C-reactive protein; HBV, hepatitis B.  Continuous variables are shown as median ± interquartile range (IQR) and categorical variables as numbers (percentage). | | | | | | | | |

| **Table S3. Comparison of baseline characteristics according to the antibody responses in BNT162b2 group** | | | | | | | | | |
| --- | --- | --- | --- | --- | --- | --- | --- | --- | --- |
| **Characteristics** |  | **High responders** (*n* = 9) | | |  | **Low responders** (*n* = 6) | | | ***p* value** |
|  |  | **V1** | **V2** | **V3** |  | **V1** | **V2** | **V3** | **GV1-PV1/GV2-PV2/GV3-PV3** |
| **Age (years)** | |  | 35.0±18.0 |  |  |  | 35.0±11.0 |  | > 0.999 |
| **Female sex (%)** | |  | 6 (60.0) |  |  |  | 4 (40.0) |  | > 0.999 |
| **BMI (kg/m^2^)** | |  | 23.5±7.1 |  |  |  | 20.6±10.1 |  | 0.814 |
| **Anti-SARS-CoV-2 S IgG (U/mL)** | | 0.4 | 181.0±120.9 | 3183.0±3222.5 |  | 0.4 | 12.9±37.6 | 808.0±351.3 | >0.999/0.001/0.001 |
| **Laboratory test results** | |  |  |  |  |  |  |  |  |
| WBC (cells/uL) | | 6200±2900 | 5500±2800 | 6500±2530 |  | 5900±1800 | 6100±2100 | 6100±2180 | 0.814/0.953/0.442 |
| ANC (cells/uL) | | 3196±2317 | 3052±2008 | 3588±2641 |  | 3148±993 | 3168±1185 | 2865±685 | 0.637/0.556/0.556 |
| Hemoglobin (g/dL) | | 13.4±3.0 | 13.7±2.8 | 13.3±3.2 |  | 13.1±3.3 | 13.3±2.2 | 12.9±2.9 | 0.479/0.768/0.595 |
| MCV (fL) | | 90.5±7.9 | 90.9±5.6 | 90.2±5.0 |  | 91.5±7.8 | 90.6±7.8 | 91.2±7.6 | >0.999/0.906/0.814 |
| MCH (pg) | | 29.9±2.0 | 29.8±2.2 | 29.7±2.5 |  | 30.2±3.3 | 30.3±3.0 | 30.6±2.4 | 0.679/0.768/0.556 |
| MCHC (g/dL) | | 33.0±0.6 | 32.9±0.65 | 33.0±1.1 |  | 33.0±1.3 | 33.5±1.4 | 33.5±1.1 | 0.471/0.441/0.213 |
| Platelet count (10^3^/uL) | | 286.0±65.5 | 298.0±107.0 | 306.0±81.0 |  | 240.5±66.0 | 257.5±101.3 | 257.0±52.3 | 0.216/0.346/0.195 |
| BUN (mg/dL) | | 13.9±4.6 | 13.3±5.8 | 12.6±4.5 |  | 12.4±6.3 | 13.4±7.1 | 11.6±5.7 | 0.953/0.953/0.723 |
| Creatinine (mg/dL) | | 0.64±0.09 | 0.63±0.13 | 0.64±0.08 |  | 0.64±0.31 | 0.61±0.14 | 0.63±0.33 | 0.953/0.906/0.678 |
| Albumin (g/dL) | | 4.40±0.25 | 4.50±0.50 | 4.50±0.40 |  | 4.25±0.58 | 4.80±0.38 | 4.55±0.55 | 0.309/0.531/0.812 |
| HDL cholesterol (mg/dL) | | 53.0±25.5 | 58.0±25.5 | 55.0±21.5 |  | 61.0±28.3 | 71.5±27.0 | 71.0±37.0 | 0.724/0.215/0.555 |
| LDL cholesterol (mg/dL) | | 113.0±41.5 | 122.0±50.5 | 127.0±36.0 |  | 103.5±68.0 | 109.5±24.3 | 105.5±58.3 | 0.409/0.480/0.111 |
| Triglyceride (mg/dL) | | 78.0±116.0 | 106.0±118.5 | 97.0±75.5 |  | 90.0±281.5 | 91.0±97.5 | 86.0±64.8 | 0.480/0.409/0.556 |
| AST (IU/L) | | 22.0±17.0 | 26.0±12.0 | 26.0±17.5 |  | 28.0±11.8 | 34.5±15.3 | 31.5±20.3 | 0.553/0.215/0.516 |
| ALT (IU/L) | | 19.0±25.5 | 21.0±34.0 | 25.0±42.5 |  | 28.0±32.0 | 24.0±45.3 | 29.5±42.3 | 0.316/0.723/0.906 |
| GGT (IU/L) | | 18.0±29.5 | 22.0±33.0 | 22.0±33.5 |  | 23.5±34.8 | 20.5±24.3 | 17.0±20.8 | 0.768/0.953/0.439 |
| Total bilirubin (mg/dL) | | 0.51±0.42 | 0.58±0.70 | 0.59±0.40 |  | 0.57±0.49 | 0.71±0.79 | 0.82±0.57 | 0.479/0.239/0.195 |
| Glucose (mg/dL) | | 93.0±34.5 | 100.0±8.0 | 99.0±19.0 |  | 98.0±68.8 | 82.5±27.0 | 97.5±27.3 | 0.814/0.237/0.768 |
| CRP (mg/L) | | 0.42±1.36 | 0.76±1.25 | 0.32±0.70 |  | 0.26±0.44 | 0.28±0.43 | 0.41±0.27 | 0.173/0.216/0.637 |
| **Underlying diseases** | |  |  |  |  |  |  |  |  |
| Hypertension | |  | 0 (0.0) |  |  |  | 2 (100.0) |  | 0.143 |
| Diabetes mellitus | |  | 0 (0.0) |  |  |  | 1 (100.0) |  | 0.400 |
| Dyslipidemia | |  | 0 (0.0) |  |  |  | 2 (100.0) |  | 0.143 |
| HBV carrier | |  | 0 (0.0) |  |  |  | 0 (0.0) |  | - |
| **GV1**, high responders V1; **GV2**, high responders V2; **GV3**, high responders V3; **PV1**, low responders V1; **PV2**, low responders V2; **PV3**, low responders V3.  BMI, body mass index; WBC, white blood cell; ANC, absolute neutrophil count; MCV, mean corpuscular volume; MCH, mean corpuscular hemoglobin; MCHC, mean corpuscular hemoglobin concentration; BUN, blood urea nitrogen; HDL, high density lipoprotein; LDL, low density lipoprotein; AST, aspartate transaminase; ALT, alanine transaminase; GGT, gamma-glutamyl transferase; CRP, C-reactive protein; HBV, hepatitis B.  Continuous variables are shown as median ± interquartile range (IQR) and categorical variables as numbers (percentage). | | | | | | | | | |

| **Table S4. Taxonomy biomarkers by linear discriminant analysis effect size (LEfSe) analysis** | | | | | | | |
| --- | --- | --- | --- | --- | --- | --- | --- |
| **ChAdOx1** | | | | | |  |  |
| **Taxon name** | **Taxon rank** | **Low responder** | **High responders** | **LDA effect size** | ***P* value** |  |  |
| *Betaproteobacteria* | Class | 0.06670 | 1.48750 | 3.89865 | 0.01722 |  |  |
| *Burkholderiales* | Order | 0.06670 | 1.38750 | 3.86464 | 0.01722 |  |  |
| *Sutterellaceae* | Family | 0.06670 | 1.37500 | 3.86101 | 0.01722 |  |  |
| *Parasutterella* | Genus | 0.00000 | 0.61250 | 3.55382 | 0.02402 |  |  |
| *Eubacterium PAC001034_s* | Species | 0.00000 | 0.13750 | 2.95184 | 0.02381 |  |  |
| *Bifidobacterium animalis group* | Species | 0.16670 | 0.00000 | 2.85243 | 0.03075 |  |  |
| *Blautia_uc* | Species | 0.00000 | 0.05000 | 2.72821 | 0.04829 |  |  |
| *Anaerotignum PAC001031_s* | Species | 0.05000 | 0.00000 | 2.56697 | 0.02969 |  |  |
| **BNT162b2** | | | | | |  |  |
| **Taxon name** | **Taxon rank** | **Low responder** | **High responders** | **LDA effect size** | ***P* value** |  |  |
| *Proteobacteria* | Phylum | 3.76670 | 1.47500 | 4.06665 | 0.01392 |  |  |
| *Ruminococcaceae PAC000661_g* | Genus | 0.00000 | 1.02500 | 3.72866 | 0.01823 |  |  |
| *Megasphaera indica* | Species | 0.81670 | 0.00000 | 3.63587 | 0.02295 |  |  |
| *Eubacterium LT907848_s* | Species | 0.16670 | 0.82500 | 3.61881 | 0.02987 |  |  |
| *Romboutsia* | Genus | 0.26670 | 0.77500 | 3.58706 | 0.04067 |  |  |
| *Bacteroides dorei* | Species | 0.51670 | 0.02500 | 3.58106 | 0.01250 |  |  |
| *Roseburia cecicola group* | Species | 0.01670 | 0.77500 | 3.56697 | 0.04119 |  |  |
| *Romboutsia timonensis* | Species | 0.26670 | 0.77500 | 3.55002 | 0.04067 |  |  |
| *Clostridium PAC001136_s* | Species | 0.00000 | 0.32500 | 3.39063 | 0.01823 |  |  |
| *Lachnospiraceae PAC001043_g* | Genus | 0.01670 | 0.25000 | 3.30157 | 0.04119 |  |  |
| *Lachnospiraceae PAC001043_g PAC001449_s* | Species | 0.00000 | 0.15000 | 3.20569 | 0.01823 |  |  |
| LDA, linear discriminant analysis. Taxonomy biomarkers found in V1 microbiota according to V3 antibody titer. | | | | | |  |  |

| **Table S5. Common taxonomic biomarkers in each time point** | | | | | | | | | | | | | |
| --- | --- | --- | --- | --- | --- | --- | --- | --- | --- | --- | --- | --- | --- |
| **ChAdOx1** | | | | | | | | | | | | | |
| **Taxonomic markers** | | | | |  | **V1 taxonomic markers** | | |  | **V2 taxonomic markers** | | |  |
| **Taxon name** | **LDA score (log 10)** | | | |  | **Taxon name** | **LDA score (log 10)** | |  | **Taxon name** | **LDA score (log 10)** | |  |
| *Parasutterella* | 3.55485 | | | |  |  |  | |  | *Escherichia coli group* | .-3.8413 | |  |
|  |  | | | |  |  |  | |  | *Escherichia* | -3.84391 | |  |
|  |  | | | |  |  |  | |  | *Bifidobacteriaceae* | -4.12477 | |  |
|  |  | | | |  |  |  | |  | *Bifidobacteriales* | -4.12477 | |  |
| **BNT162b2** | | | | | | | | | | | | |  |
| **Taxonomic markers** | | | | |  | **V1 taxonomic markers** | | |  | **V2 taxonomic markers** | | |  |
| **Taxon name** | | | **LDA score (log 10)** | |  | **Taxon name** | **LDA score (log 10)** | |  | **Taxon name** | **LDA score (log 10)** | |  |
| *Eubacterium LT907848_s* | | | 3.61881 | |  | *Romboutsia* | 3.47008 | |  | *Roseburia cecicola group* | | dfdfffffffffffffffffffffffffffffffffffffffffffffffffffffffffffffffffffffffffff3.47193 |  |
| *Romboutsia* | ……………………………………………………… ……………………………3.58706 | | | |  | *Romboutsia timonensis* | 3.46984 | |  | *Parasutterella* | 3.38126 | |  |
| *Roseburia cecicola group* | | 3.56697 | | |  | *Eubacterium LT907848_s* | | 3.25248 |  | *Blautia AJ408961_s* | 3.26068 | |  |
| *Romboutsia timonensis* | 3.55002 | | | |  | *Blautia AJ408961_s* | 3.12932 | |  | *Bacteroides dorei* | -3.49332 | |  |
| *Clostridium PAC001136_s* | | | | 3.39063 |  | *Clostridium PAC001136_s* | | 3.05463 |  | *Campylobacterales* | -4.12274 | |  |
| *Bacteroides dorei* | -3.58106 | | | |  | *Megasphaera indica* | -3.45518 | |  | *Epsilonproteobacteria* | -4.12331 | |  |
| *Megasphaera indica* | -3.63587 | | | |  | *Bacteroides dorei* | -3.87908 | |  | *Campylobacter* | -4.12395 | |  |
| *Proteobacteria* | -4.06665 | | | |  | *Campylobacteraceae* | -4.06762 | |  | *Campylobacteraceae* | -4.12485 | |  |
|  |  | | | |  | *Epsilonproteobacteria* | -4.0679 | |  |  |  | |  |
|  |  | | | |  | *Campylobacter* | -4.06805 | |  |  |  | |  |
|  |  | | | |  | *Campylobacterales* | -4.06835 | |  |  |  | |  |
|  |  | | | |  | *Bifidobacteriaceae* | -4.06882 | |  |  |  | |  |
|  |  | | | |  | *Bifidobacteriales* | -4.06882 | |  |  |  | |  |
|  |  | | | |  | *Escherichia* | -4.3722 | |  |  |  | |  |
|  |  | | | |  | *Escherichia coli group* | -4.37341 | |  |  |  | |  |
|  |  | | | |  | *Proteobacteria* | -4.55491 | |  |  |  | |  |
| LDA, linear discriminant analysis. Taxonomic biomarkers commonly found at two or more of the three times points (taxonomic markers in V1 based on the antibody immune responses of V3, taxonomic markers in V1 based on the antibody immune responses of V2, and taxonomic biomarkers in V2 based on the antibody immune responses of V3) are shown here. | | | | | | | | | | | | | |

| **Table S6. Functional biomarkers by linear discriminant analysis effect size (LEfSe) analysis** | | | | | | | | |
| --- | --- | --- | --- | --- | --- | --- | --- | --- |
| **ChAdOx1** | | | | | | |  |  |
| **Ortholog** | **Definition** | **Low responder** | **High responders** | **LDA effect size** | ***P* value** | ***P* value (FDR)** |  |  |
| K01179 | endoglucanase | 0.0532 | 0.08 | 2.207036 | 0.020137 | 0.896388 |  |  |
| K01676 | fumarate hydratase, class I | 0.0157 | 0.0227 | 1.666197 | 0.038867 | 0.896388 |  |  |
| K02478 | two-component system, LytTR family, sensor kinase | 0.0102 | 0.0178 | 1.658674 | 0.020137 | 0.896388 |  |  |
| K01657 | anthranilate synthase component I | 0.0271 | 0.035 | 1.603838 | 0.038867 | 0.896388 |  |  |
| K00248 | butyryl-CoA dehydrogenase | 0.0131 | 0.0064 | 1.525516 | 0.014171 | 0.896388 |  |  |
| K07662 | two-component system, OmpR family, response regulator CpxR | 0.0463 | 0.0363 | 1.813081 | 0.038867 | 0.896388 |  |  |
| K02033 | peptide/nickel transport system permease protein | 0.0717 | 0.0532 | 2.062776 | 0.038867 | 0.896388 |  |  |
| K02003 | putative ABC transport system ATP-binding protein | 0.2344 | 0.1901 | 2.393052 | 0.020137 | 0.896388 |  |  |
| **BNT162b2** | | | | | | |  |  |
| **Ortholog** | **Definition** | **Low responder** | **High responders** | **LDA effect size** | ***P* value** | ***P* value (FDR)** |  |  |
| K03392 | aminocarboxymuconate-semialdehyde decarboxylase | 0.0181 | 0.0653 | 2.397185 | 0.019016 | 0.673289 |  |  |
| K02013 | iron complex transport system ATP-binding protein | 0.0923 | 0.116 | 2.180459 | 0.019016 | 0.673289 |  |  |
| K01593 | aromatic-L-amino-acid/L-tryptophan decarboxylase | 0.0076 | 0.0249 | 2.00677 | 0.033006 | 0.673289 |  |  |
| K03567 | glycine cleavage system transcriptional repressor | 0.0174 | 0.0342 | 1.957881 | 0.010515 | 0.673289 |  |  |
| K02006 | cobalt/nickel transport system ATP-binding protein | 0.0158 | 0.0308 | 1.957086 | 0.033006 | 0.673289 |  |  |
| K01975 | RNA 2',3'-cyclic 3'-phosphodiesterase | 0.0177 | 0.033 | 1.922388 | 0.033006 | 0.673289 |  |  |
| K01966 | propionyl-CoA carboxylase beta chain | 0.0182 | 0.0312 | 1.910368 | 0.033006 | 0.673289 |  |  |
| K02021 | putative ABC transport system ATP-binding protein | 0.0187 | 0.032 | 1.88189 | 0.019016 | 0.673289 |  |  |
| K00534 | ferredoxin hydrogenase small subunit | 0.0207 | 0.0344 | 1.872734 | 0.033006 | 0.673289 |  |  |
| K03451 | betaine/carnitine transporter, BCCT family | 0.0159 | 0.0298 | 1.838406 | 0.033006 | 0.673289 |  |  |
| K02022 | HlyD family secretion protein | 0.0248 | 0.0377 | 1.838199 | 0.019016 | 0.673289 |  |  |
| K01011 | thiosulfate/3-mercaptopyruvate sulfurtransferase | 0.0078 | 0.0187 | 1.807469 | 0.033006 | 0.673289 |  |  |
| K00654 | serine palmitoyltransferase | 0.0093 | 0.02 | 1.77698 | 0.019016 | 0.673289 |  |  |
| K01420 | CRP/FNR family transcriptional regulator, anaerobic regulatory protein | 0.0111 | 0.0217 | 1.77055 | 0.010515 | 0.673289 |  |  |
| K06817 | podocalyxin-like | 0.0157 | 0.0278 | 1.767455 | 0.033006 | 0.673289 |  |  |
| K02687 | ribosomal protein L11 methyltransferase | 0.0458 | 0.036 | 1.739812 | 0.033006 | 0.673289 |  |  |
| K00286 | pyrroline-5-carboxylate reductase | 0.05 | 0.0421 | 1.749211 | 0.019016 | 0.673289 |  |  |
| K03218 | 23S rRNA (guanosine2251-2'-O)-methyltransferase | 0.0618 | 0.0504 | 1.790968 | 0.019016 | 0.673289 |  |  |
| K04068 | anaerobic ribonucleoside-triphosphate reductase activating protein | 0.057 | 0.0446 | 1.803531 | 0.019016 | 0.673289 |  |  |
| K09765 | epoxyqueuosine reductase | 0.0429 | 0.0328 | 1.807832 | 0.033006 | 0.673289 |  |  |
| K05808 | putative sigma-54 modulation protein | 0.0521 | 0.0401 | 1.817599 | 0.033006 | 0.673289 |  |  |
| K09022 | 2-iminobutanoate/2-iminopropanoate deaminase | 0.0656 | 0.0546 | 1.825438 | 0.033006 | 0.673289 |  |  |
| K05837 | rod shape determining protein RodA | 0.0538 | 0.0423 | 1.826586 | 0.033006 | 0.673289 |  |  |
| K02237 | competence protein ComEA | 0.0561 | 0.0427 | 1.842621 | 0.033006 | 0.673289 |  |  |
| K01303 | acylaminoacyl-peptidase | 0.0308 | 0.0175 | 1.87819 | 0.033006 | 0.673289 |  |  |
| LDA, linear discriminant analysis. Taxonomy prognostic markers found in V1 microbiota according to V3 antibody titer. | | | | | | |  |  |

| **Table S7. Common functional biomarkers in each time point** | | | | | | | | | | | | | | | | |
| --- | --- | --- | --- | --- | --- | --- | --- | --- | --- | --- | --- | --- | --- | --- | --- | --- |
| **ChAdOx1** | | | | | | | | | | | | | | | | |
| **Functional markers** | | |  | **V1 functional markers** | | | |  | **V2 functional markers** | | | | | | |  |
| **Ortholog** | **Definition** | **LDA score (log 10)** |  | **Ortholog** | **Definition** | **LDA score (log 10)** | |  | **Ortholog** | | | **Definition** | **LDA score (log 10)** | | |  |
| K01179 | endoglucanase | 2.207036 |  | K00244 | fumarate reductase flavoprotein subunit | 2.138837 | |  |  | | | **-** |  | | |  |
| K01657 | anthranilate synthase component I | 1.603838 |  |  |  |  | |  |  | | |  |  | | |  |
| **BNT162b2** | | | | | | | | | | | | | | | |  |
| **Functional markers** | | |  | **V1 functional markers** | | | |  | **V2 functional markers** | | | | | | |  |
| **Ortholog** | **Definition** | **LDA score (log 10)** |  | **Ortholog** | **Definition** | | **LDA score (log 10)** |  | **Ortholog** | | **Definition** | | | **LDA score (log 10)** | |  |
| K03392 | aminocarboxymuconate-semialdehyde decarboxylase | 2.397185 |  | K03392 | aminocarboxymuconate-semialdehyde decarboxylase | | 2.314161 |  | K01179 | | endoglucanase | | | 2.265260 | |  |
| K02013 | iron complex transport system ATP-binding protein | 2.180459 |  | K02013 | iron complex transport system ATP-binding protein | | 1.943087 |  | K00244 | | fumarate reductase flavoprotein subunit | | | 2.167653 | |  |
| K01593 | aromatic-L-amino-acid/L-tryptophan decarboxylase | 2.006770 |  | K01593 | aromatic-L-amino-acid/L-tryptophan decarboxylase | | 1.832424 |  | K15578 | | nitrate/nitrite transport system ATP-binding protein | | | -1.905914 | |  |
| K02006 | cobalt/nickel transport system ATP-binding protein | 1.957086 |  | K01657 | anthranilate synthase component I | | 1.817083 |  |  | |  | | |  | |  |
| K02021 | putative ABC transport system ATP-binding protein | 1.881890 |  | K02006 | cobalt/nickel transport system ATP-binding protein | | 1.797934 |  |  |  | | | | |  |  |
|  |  |  |  | K02021 | putative ABC transport system ATP-binding protein | | 1.793769 |  |  | |  | | |  | |  |
|  |  |  |  | K15578 | nitrate/nitrite transport system ATP-binding protein | | -1.867171 |  |  | |  | | |  | |  |
| LDA, linear discriminant analysis. functional biomarkers commonly found at two or more of the three times points (functional markers in V1 based on the antibody immune responses of V3, functional markers in V1 based on the antibody immune responses of V2, and functional biomarkers in V2 based on the antibody immune responses of V3) are presented. | | | | | | | | | | | | | | | | |

**Figure S1. Changes in alpha diversity indexes (ACE, CHAO, Jackknife, NP Shannon, Simpson, and phylogenetic diversity) following vaccination.** Changes in fecal microbiota alpha diversity based on the ACE (a), CHAO (b), Jackknife (c), NP Shannon (d), Simpson (e), and phylogenetic diversity (f) are presented following ChAdOx1 (left side, magenta color) and BNT162b2 vaccination (right side, cyan color), respectively.

**(a) ACE**

**(b) CHAO**

**ChAdOx1**

**BNT162b2**


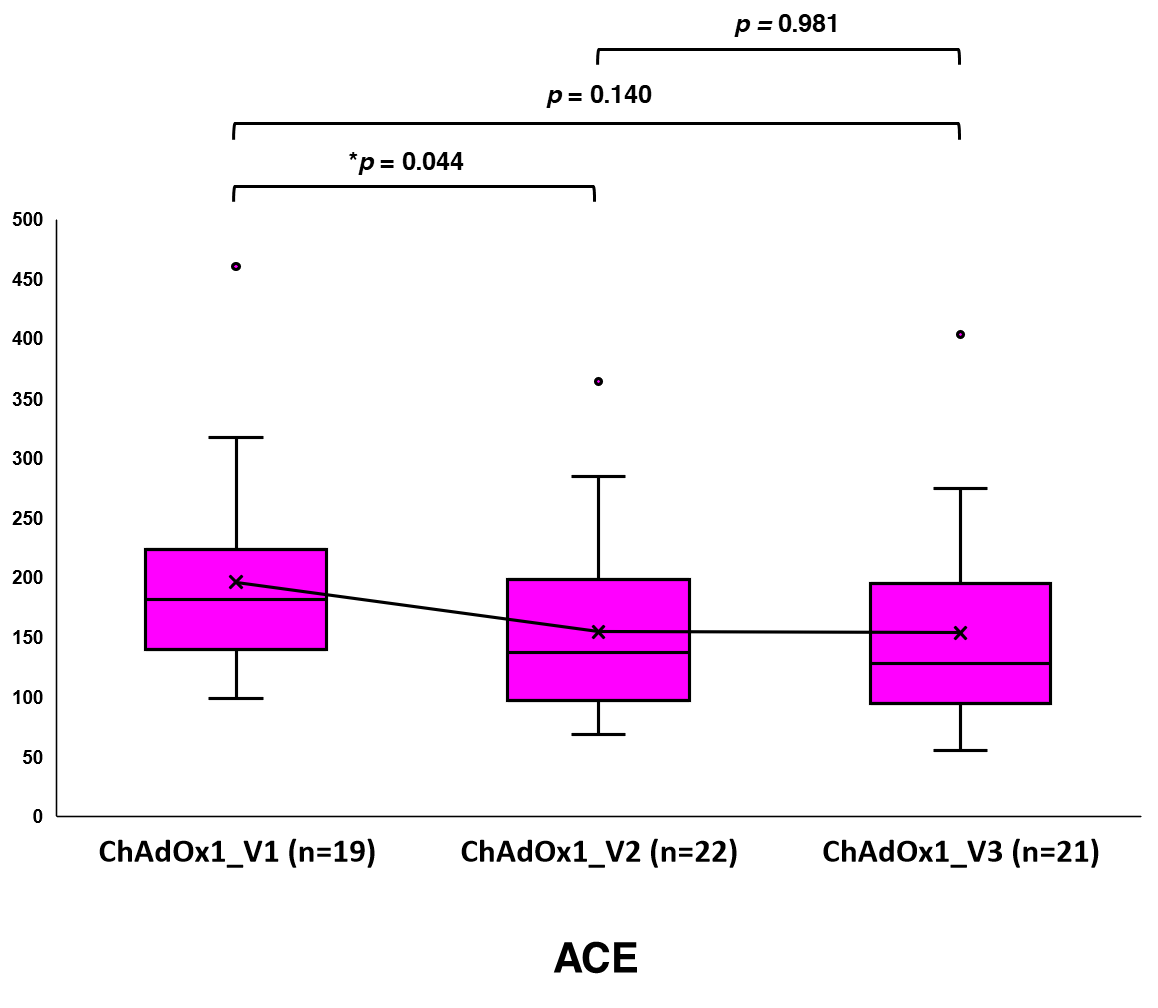

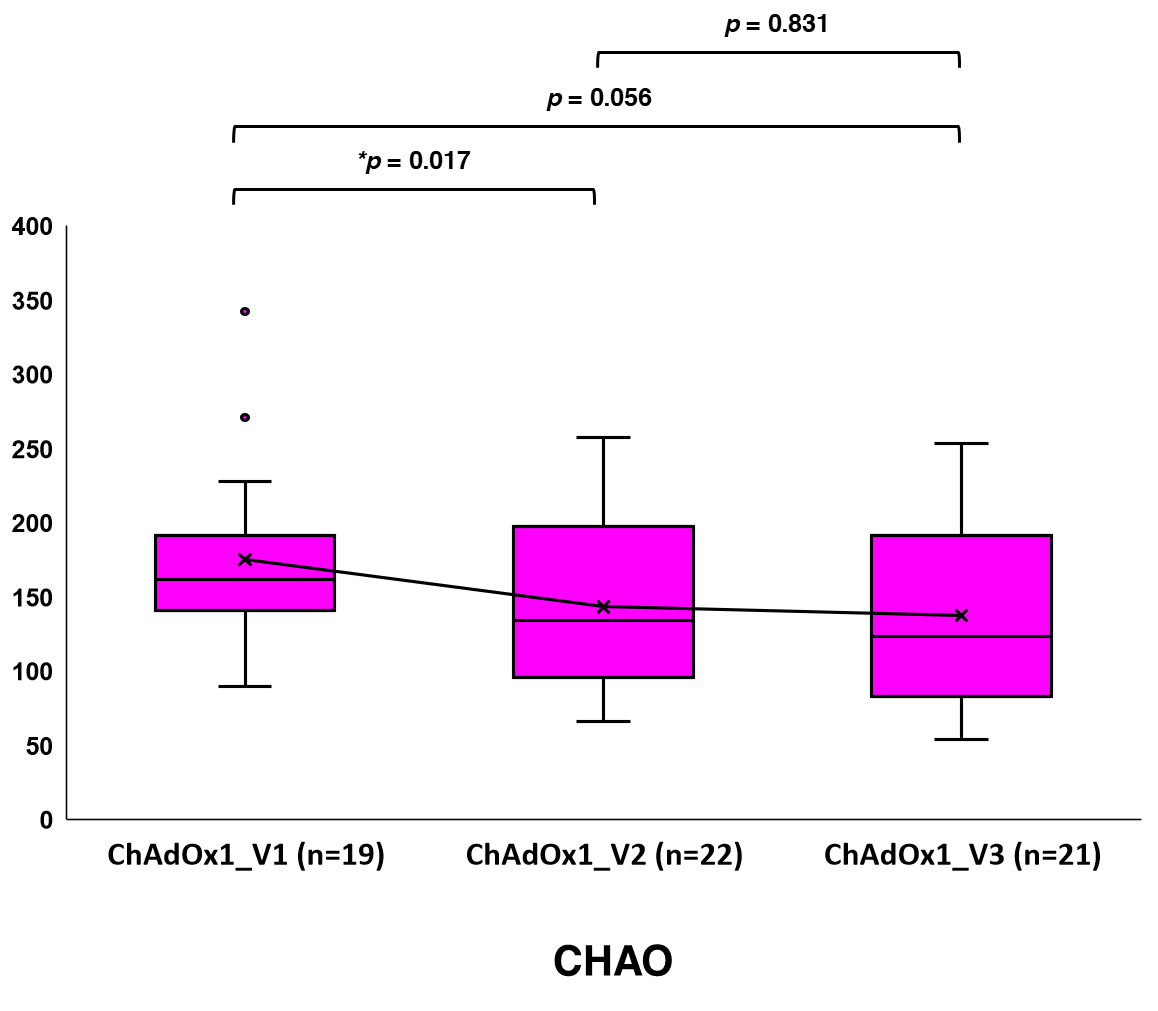

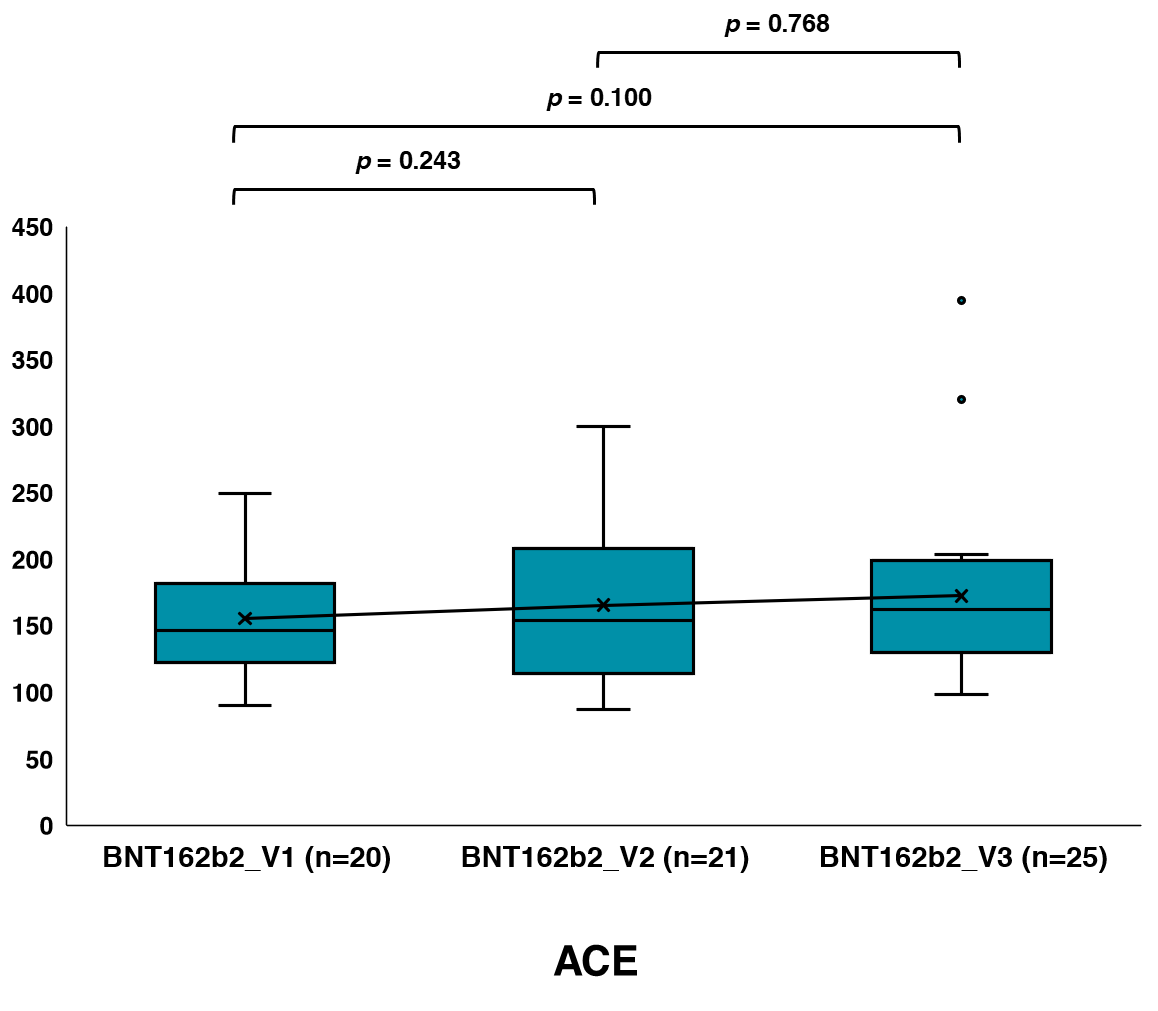

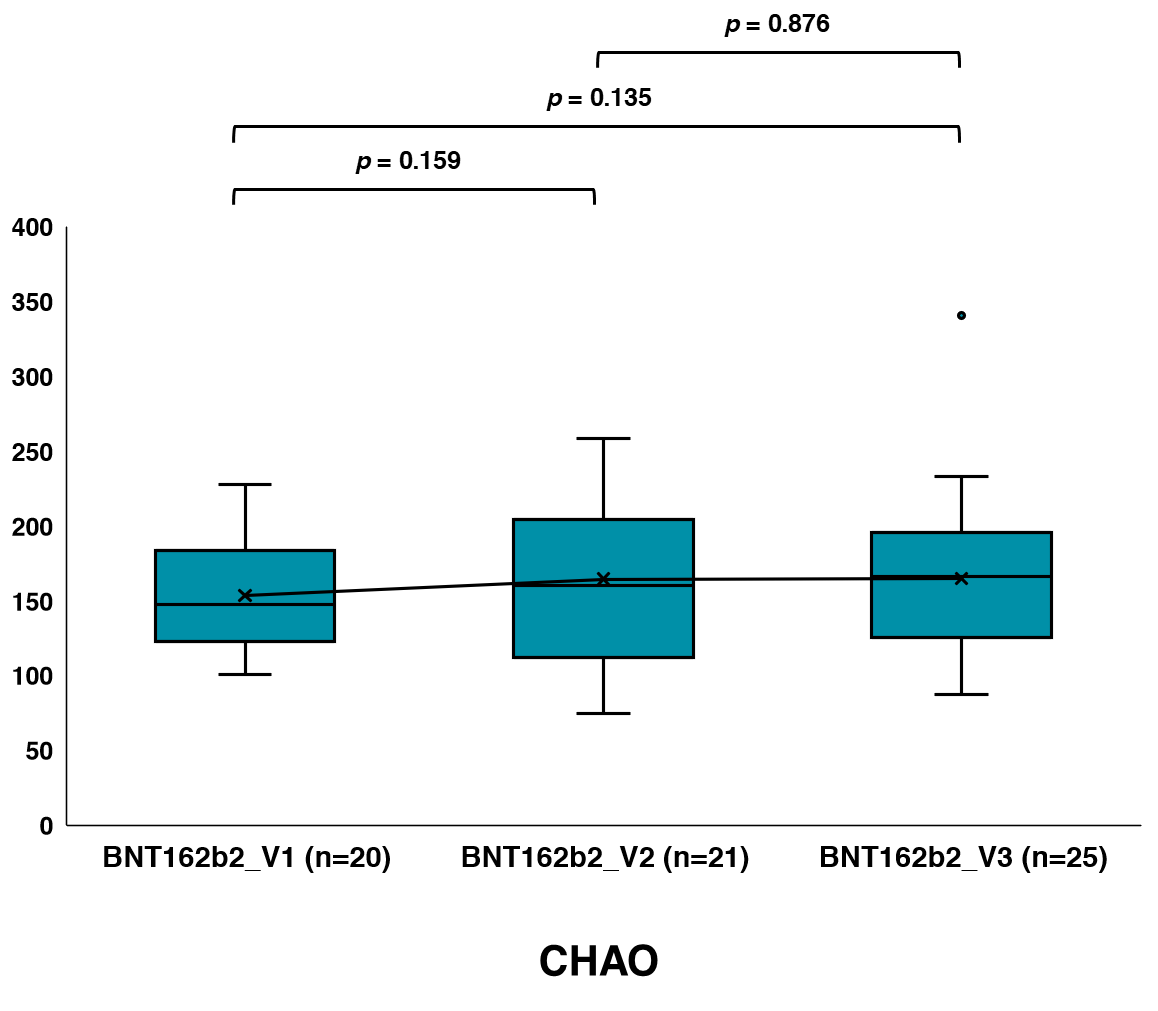


**Figure S2. Ternary plot (phylum, class, order, family, and species).** The microbiota changes in phylum (a), class (b), order (c), family (d), and species-level (e) on V1, V2, and V3 are presented as ternary plots for the ChAdOx1-vaccinated group on the left side and BNT162b2-vaccinated group on the right side.

**(c) Jackknife**

**ChAdOx1**

**BNT162b2**

**(d) NP Shannon**


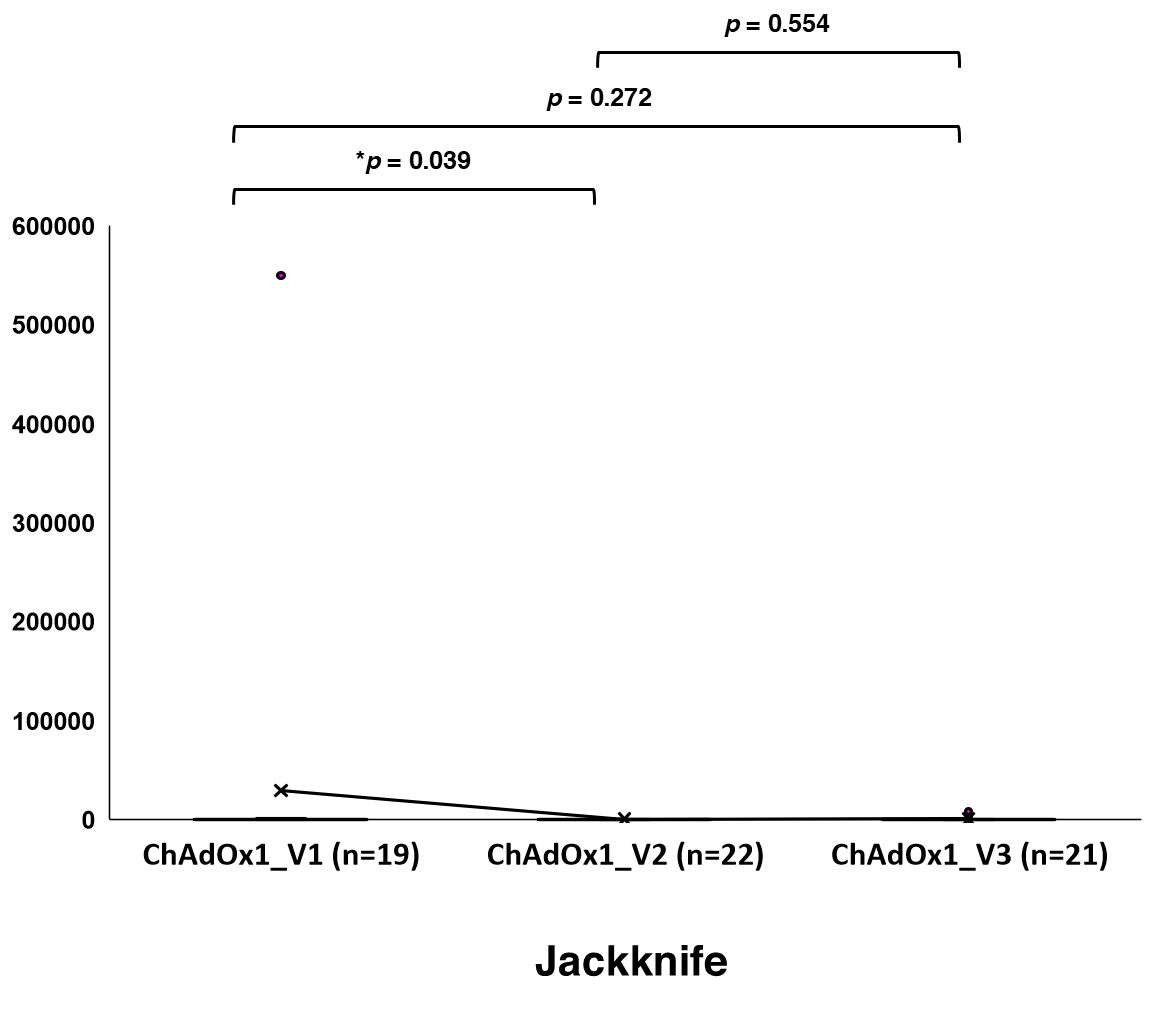

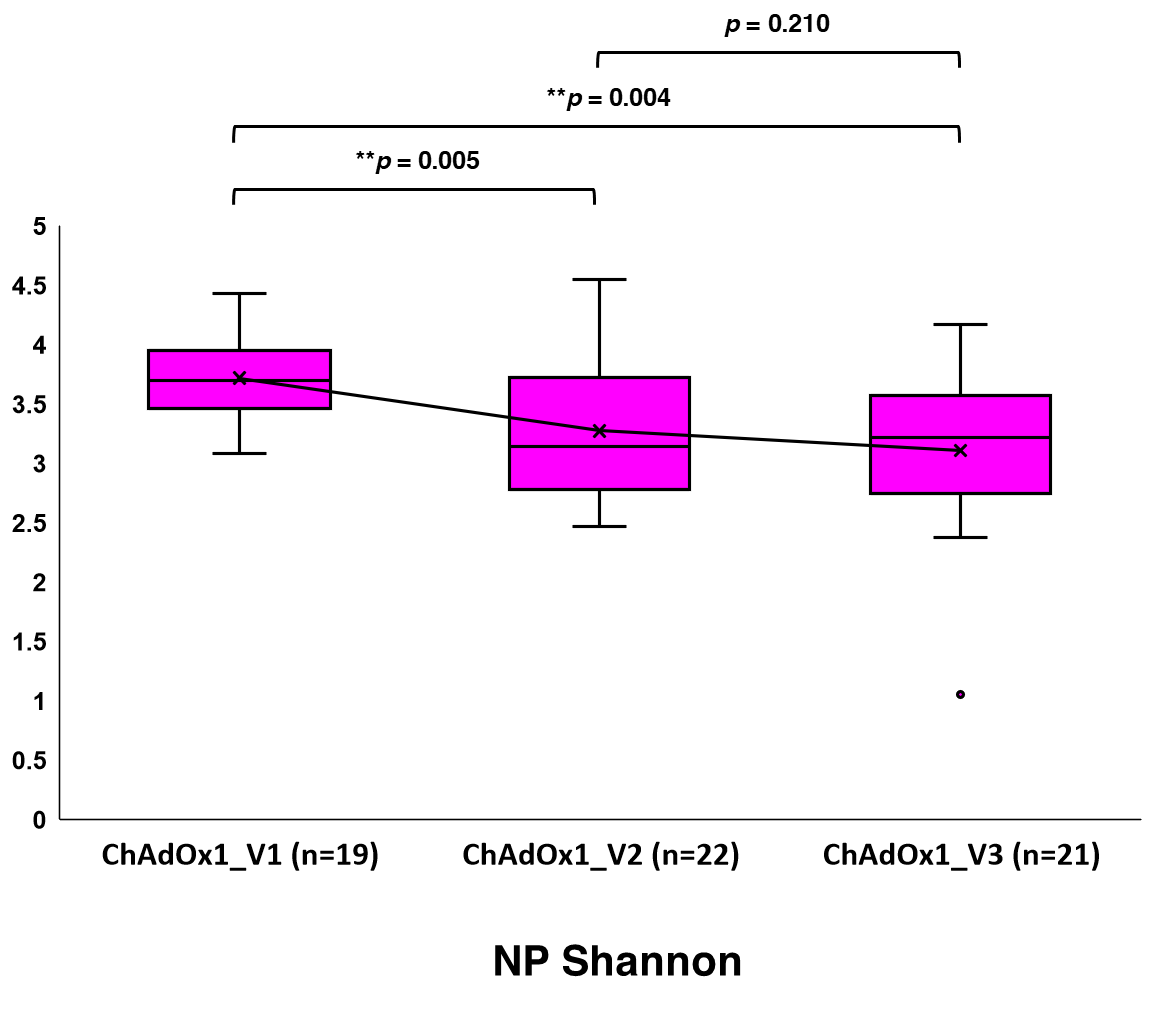

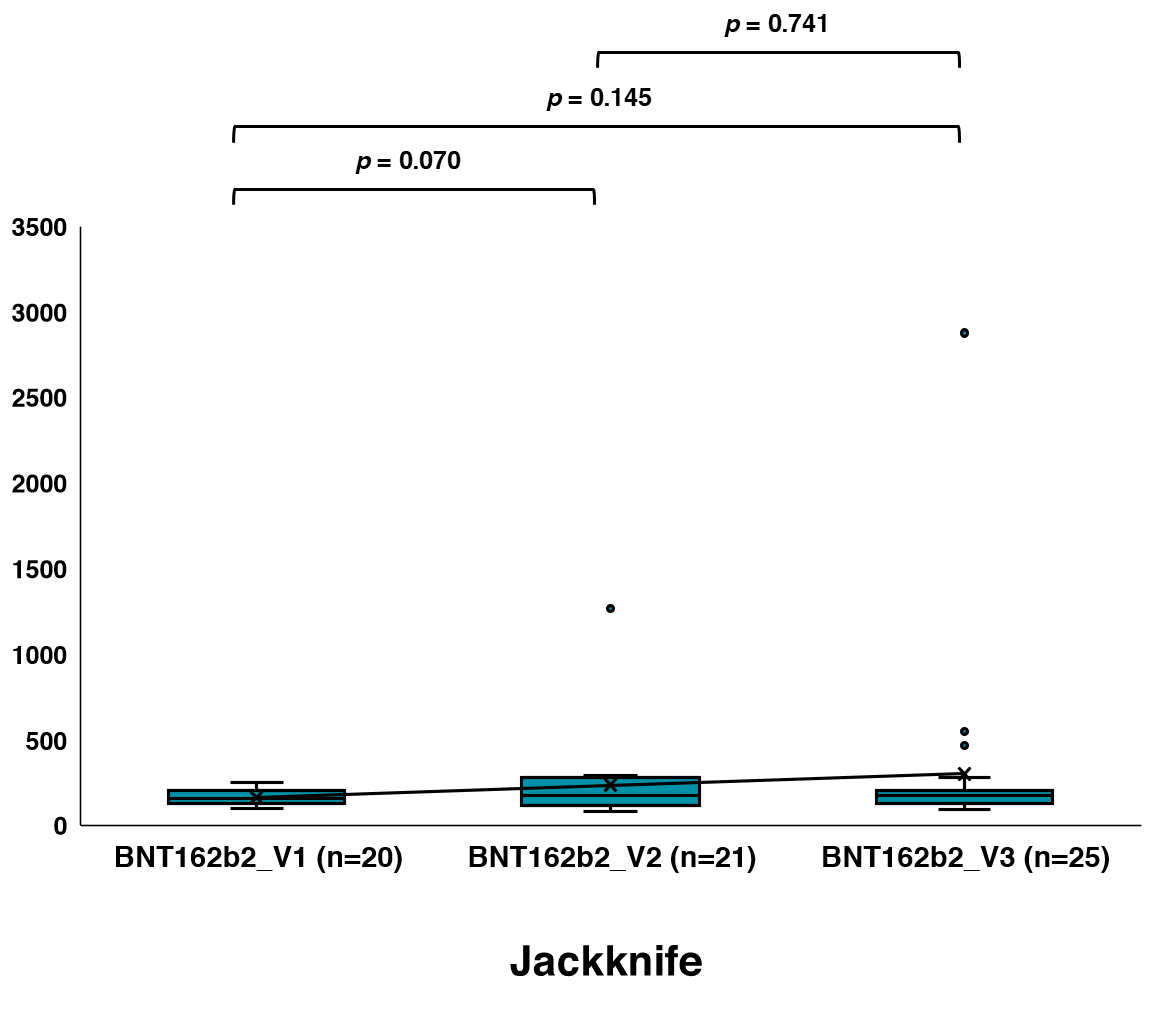

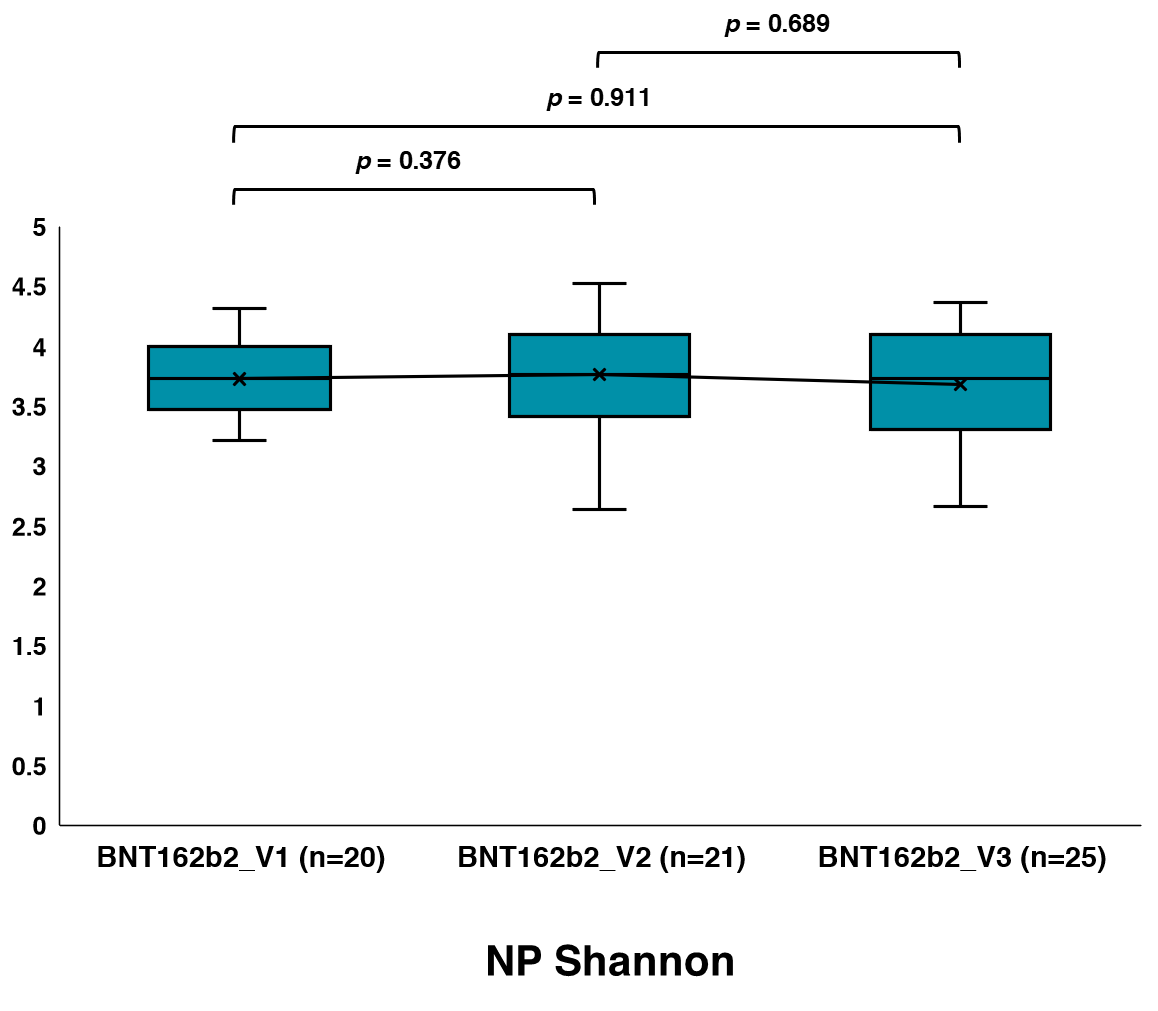


**ChAOx1**

**BNT162b2**

**(e) Simpson**

**(f) Phylogenetic Diversity**


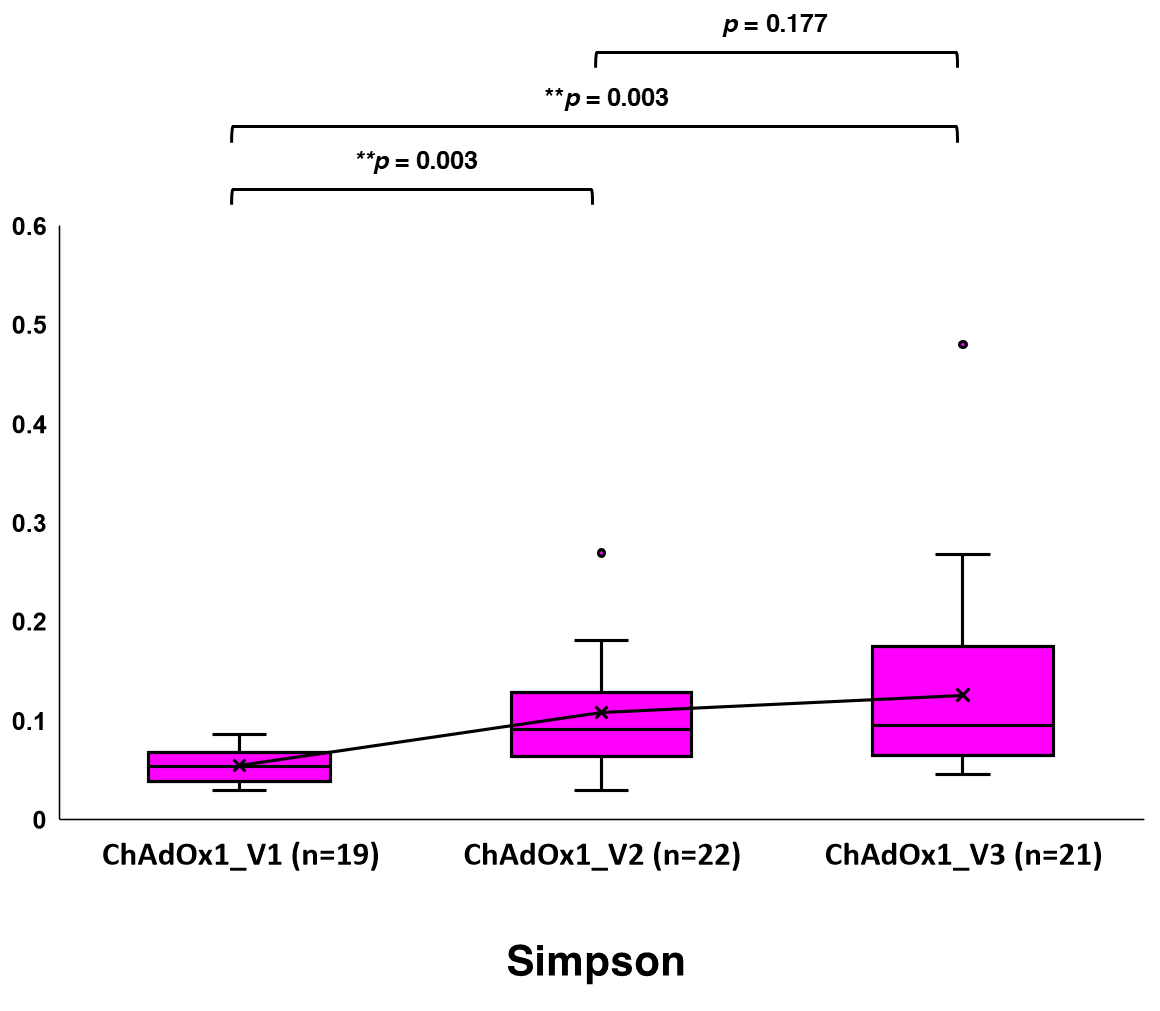

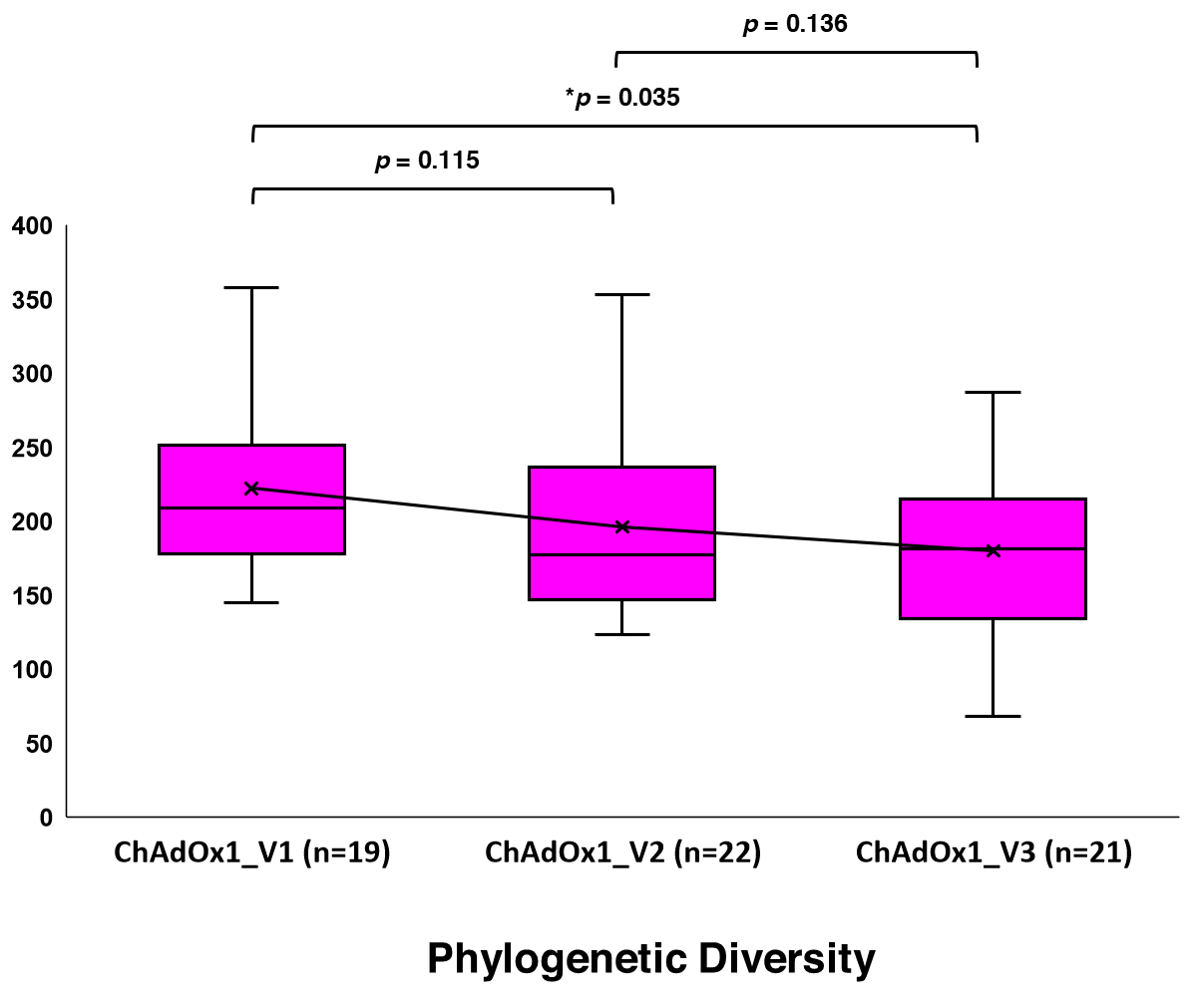

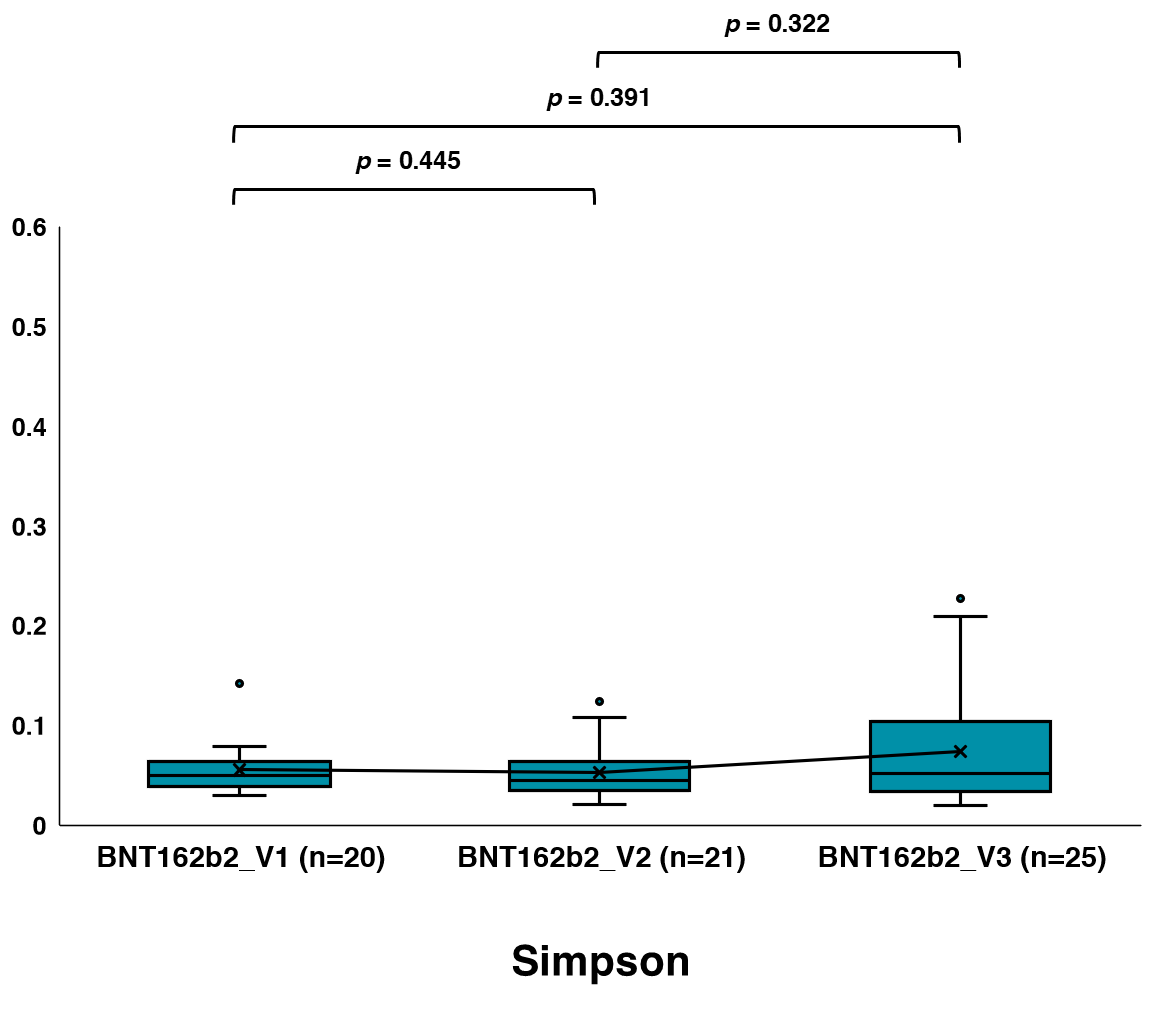

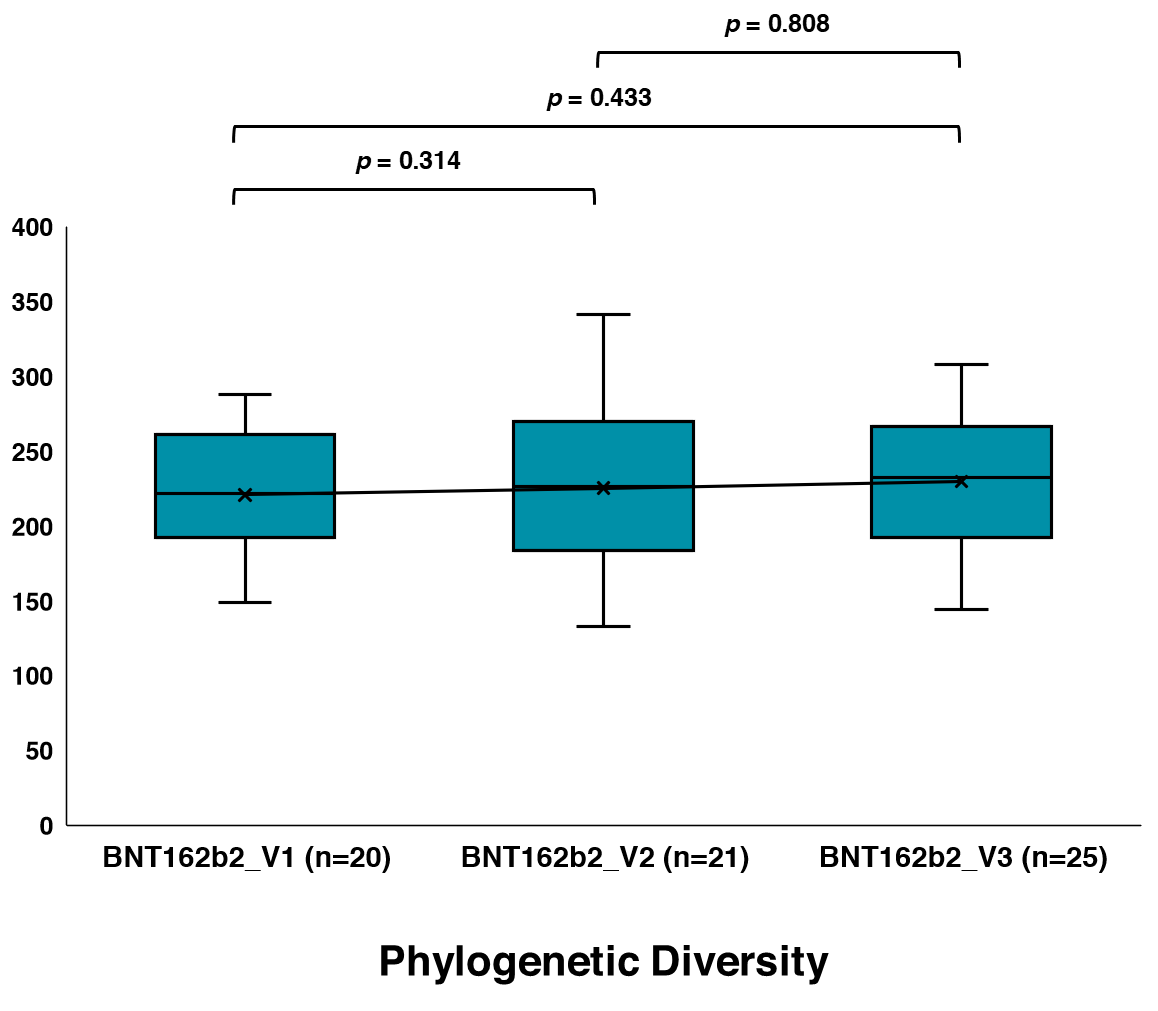

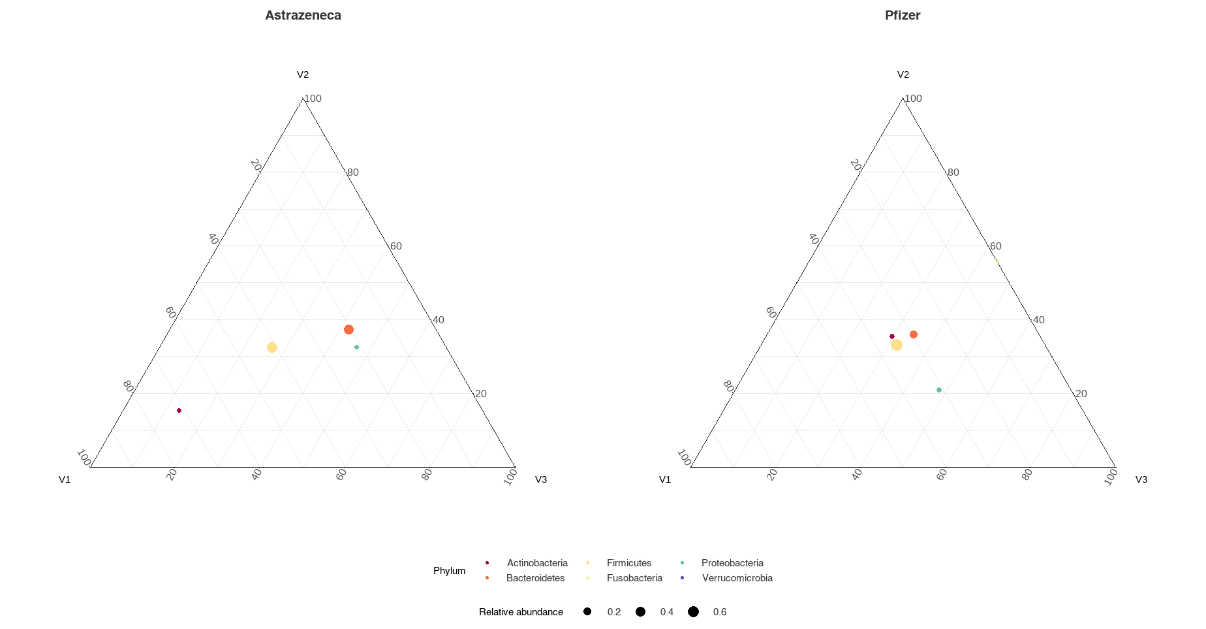

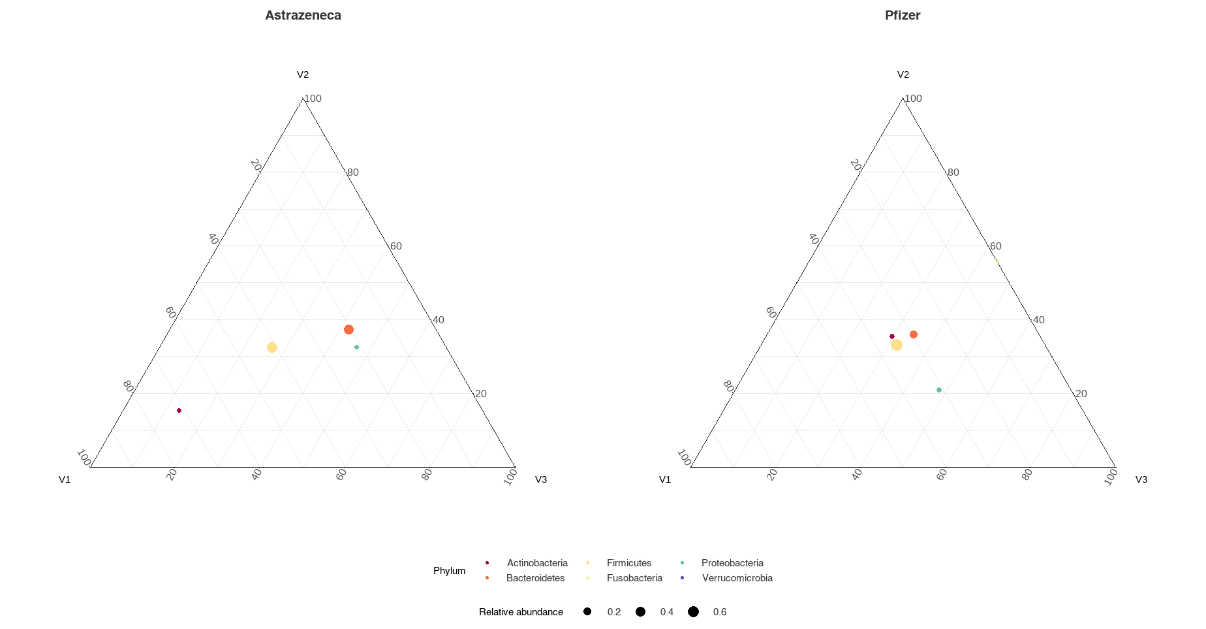


**BNT162b2**

**(a)**

**ChAdOx1**

**(b)**


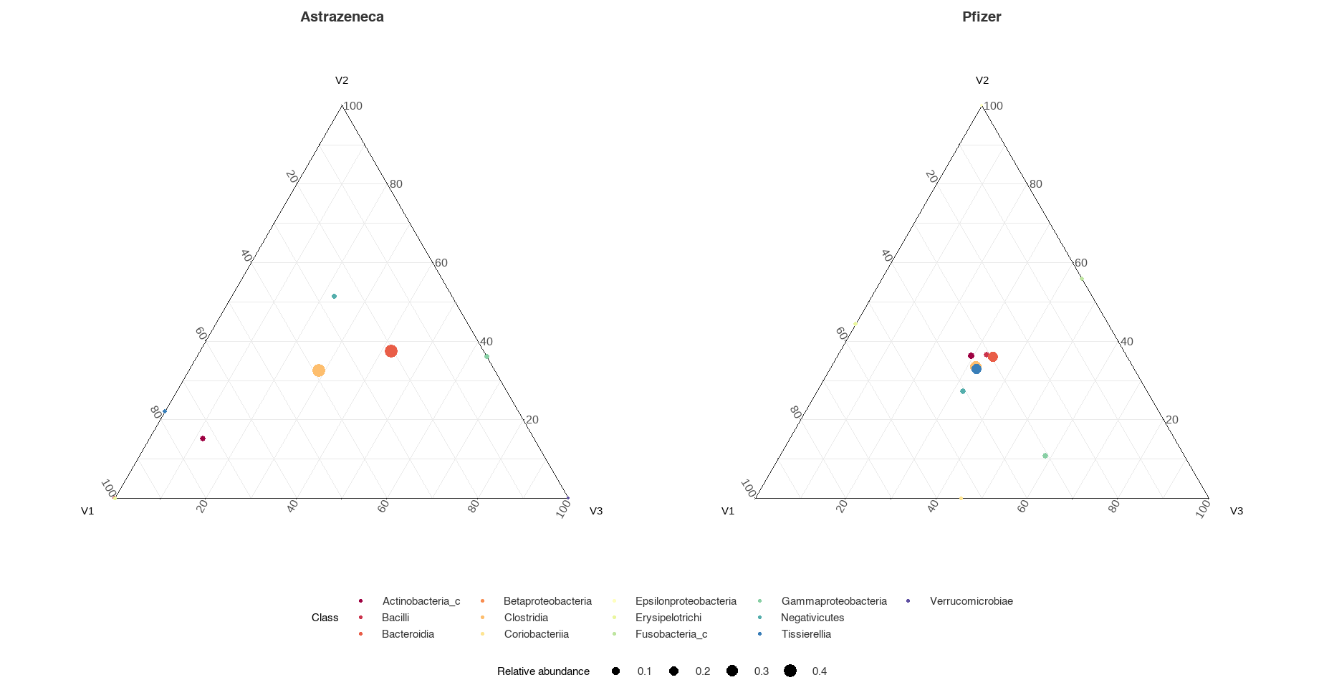

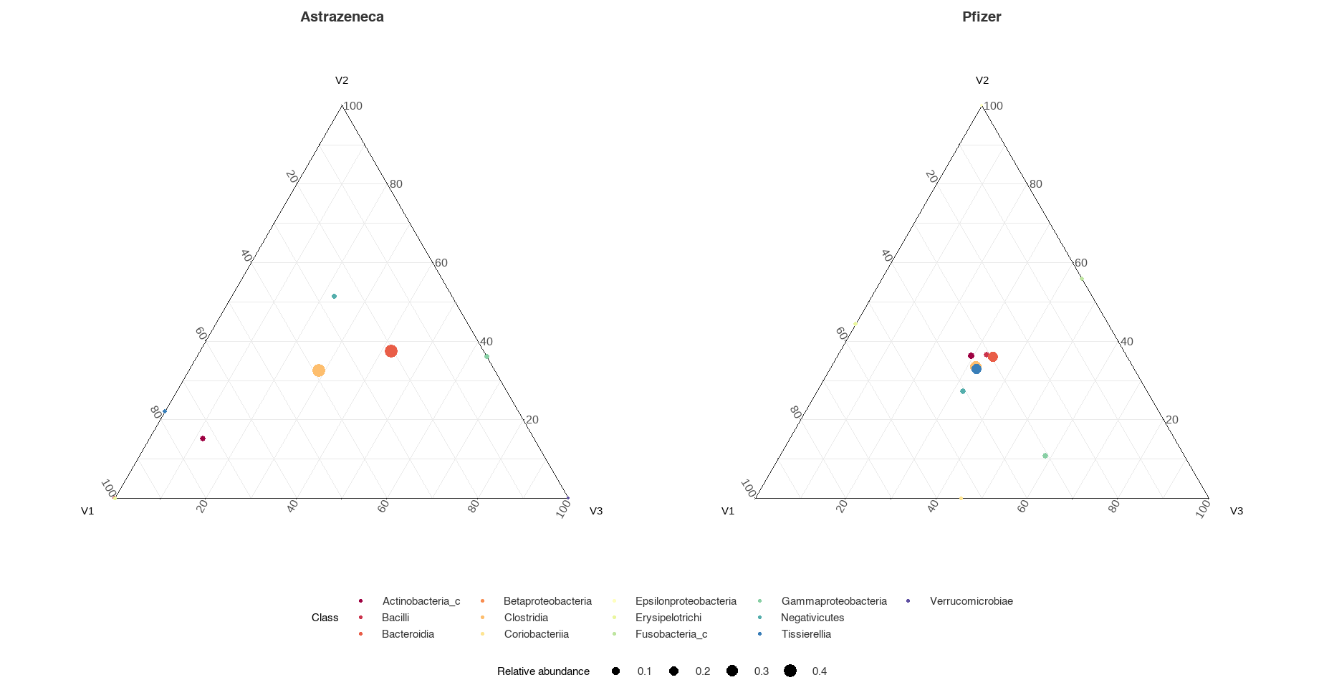

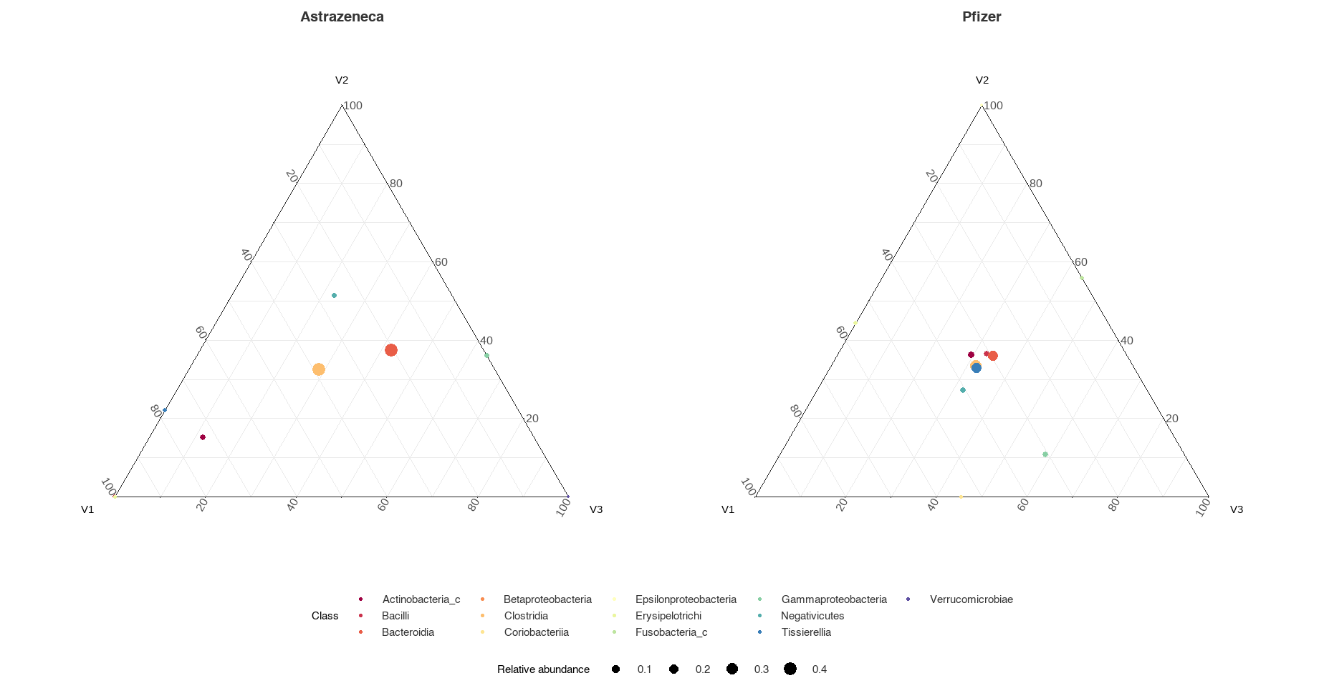


**Figure S3. Baseline microbiota differences in alpha diversity indexes (CHAO, Jackknife, Shannon, NP Shannon, Simpson, and phylogenetic diversity) according to the immune response in vaccinated groups.** The baseline differences in fecal microbiota alpha diversity based on the CHAO (a), Jackknife (b), NP Shannon (c), Shannon (d), Simpson (e), and phylogenetic diversity (f) are presented in ChAdOx1 and BNT162b2 vaccination groups, respectively.


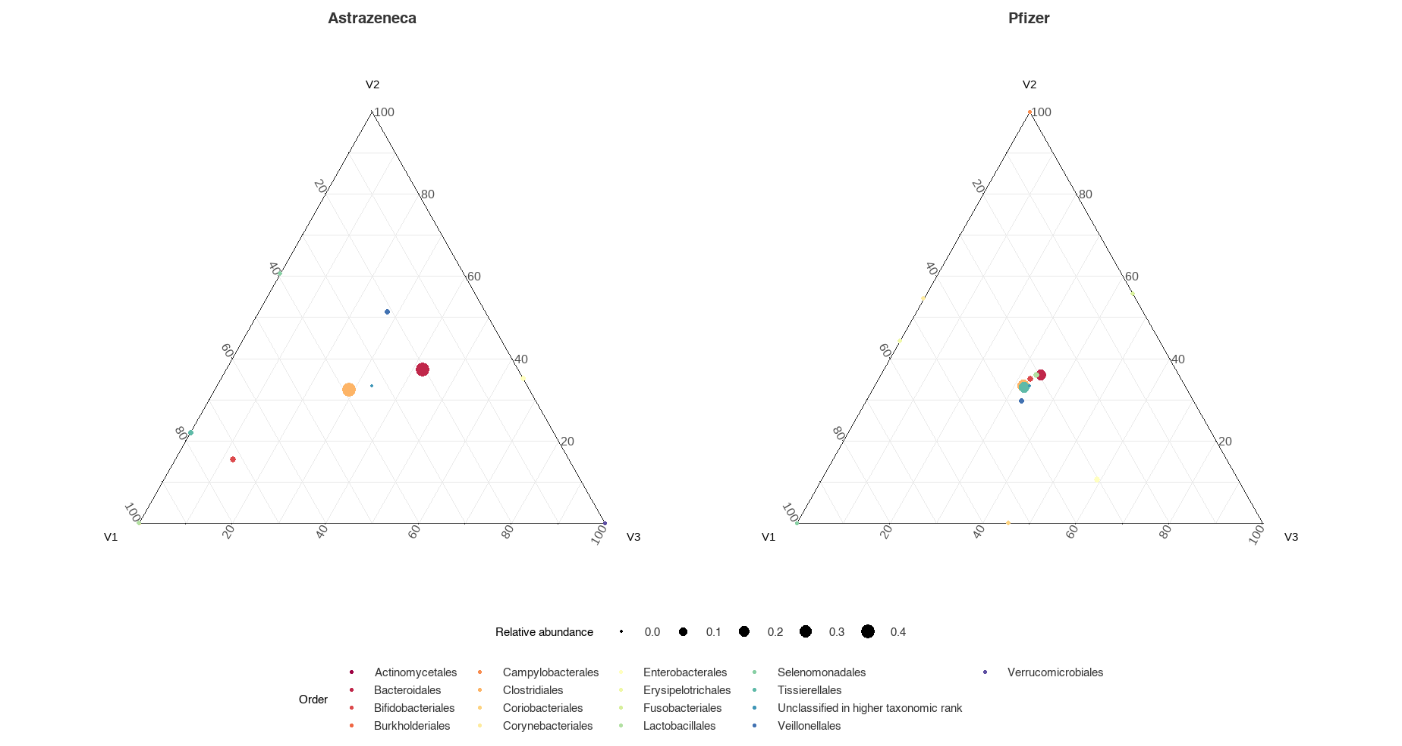

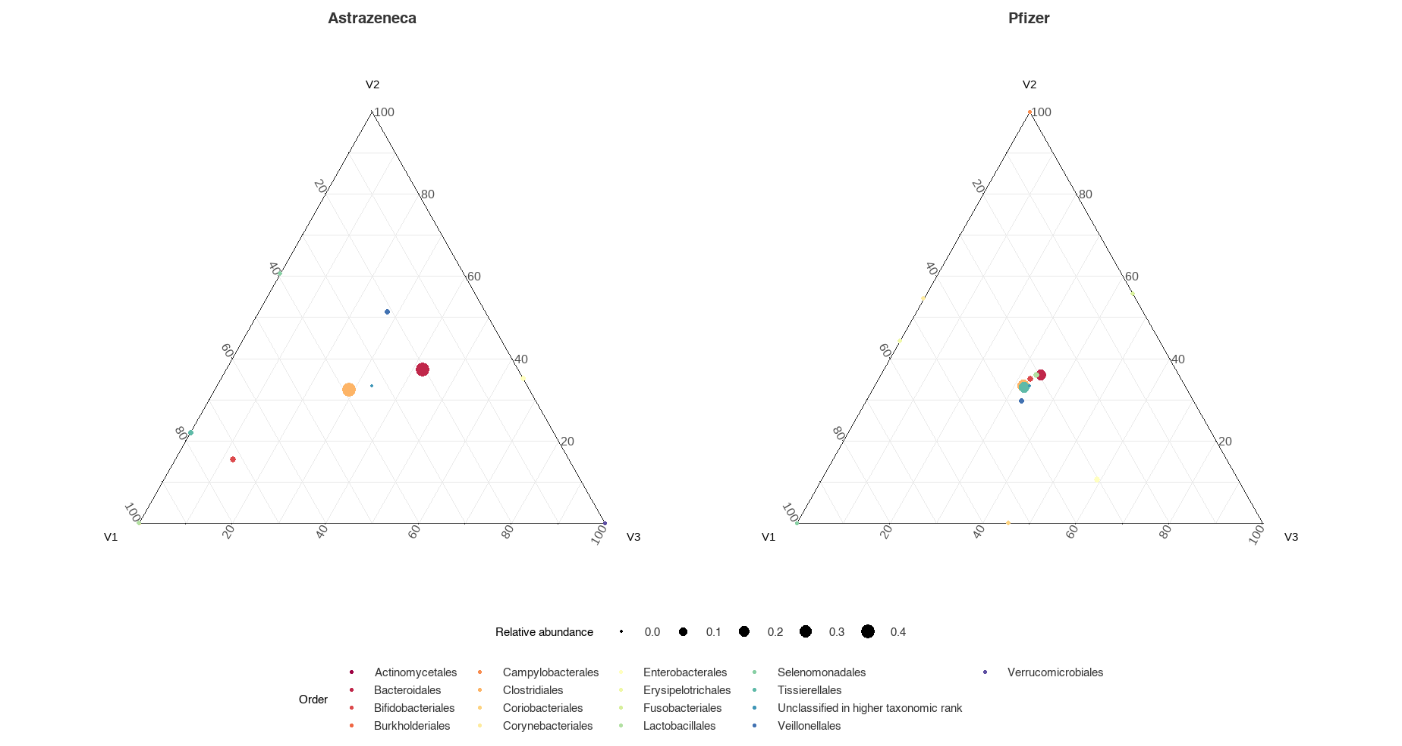

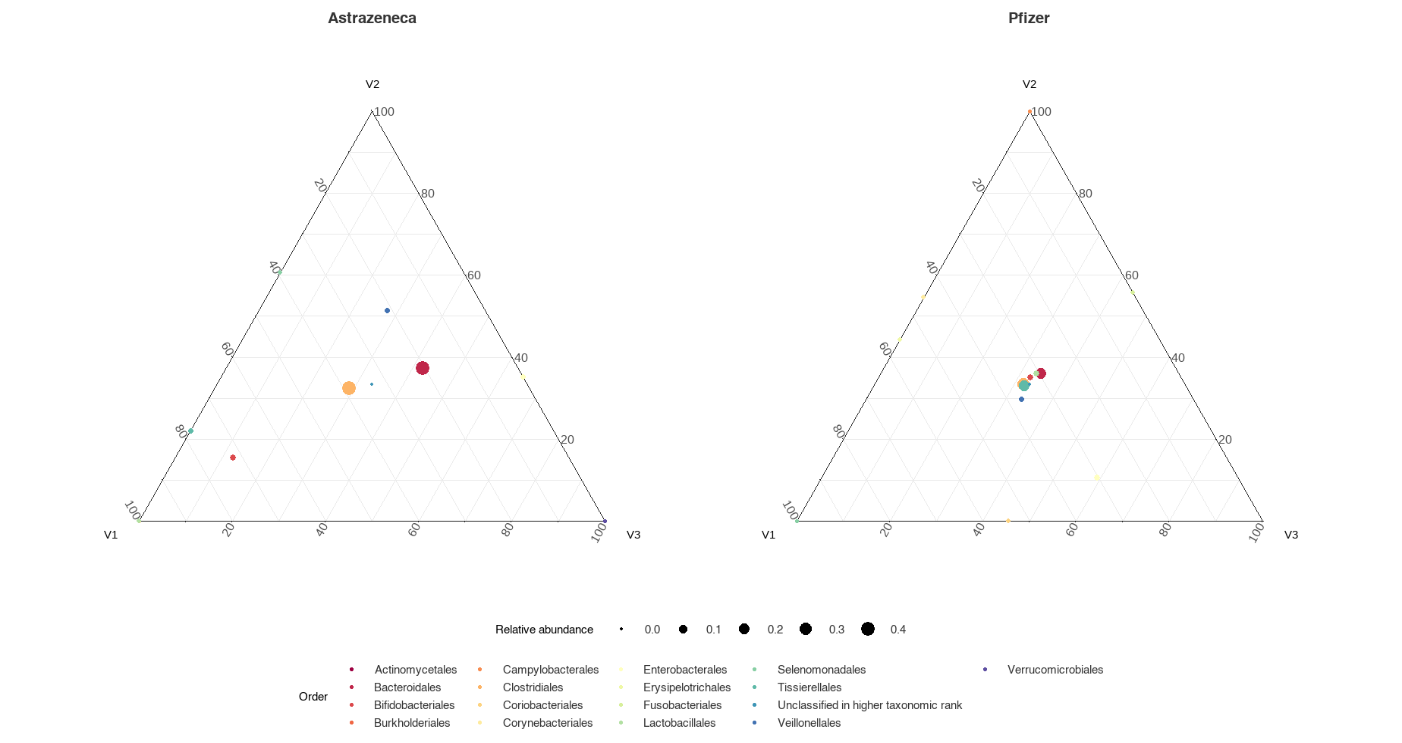


**BNT162b2**

**(c)**

**ChAdOx1**

**(d)**


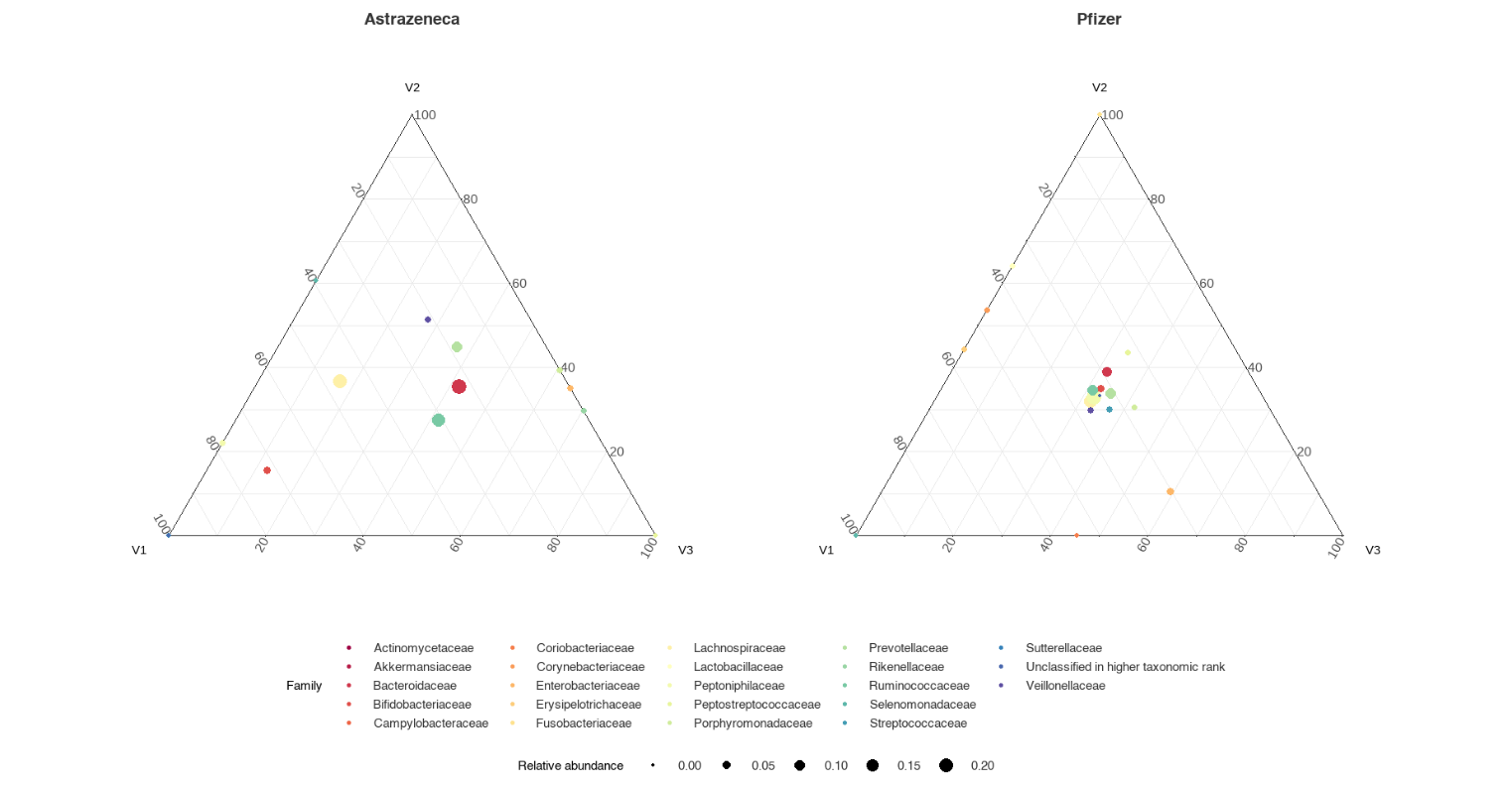

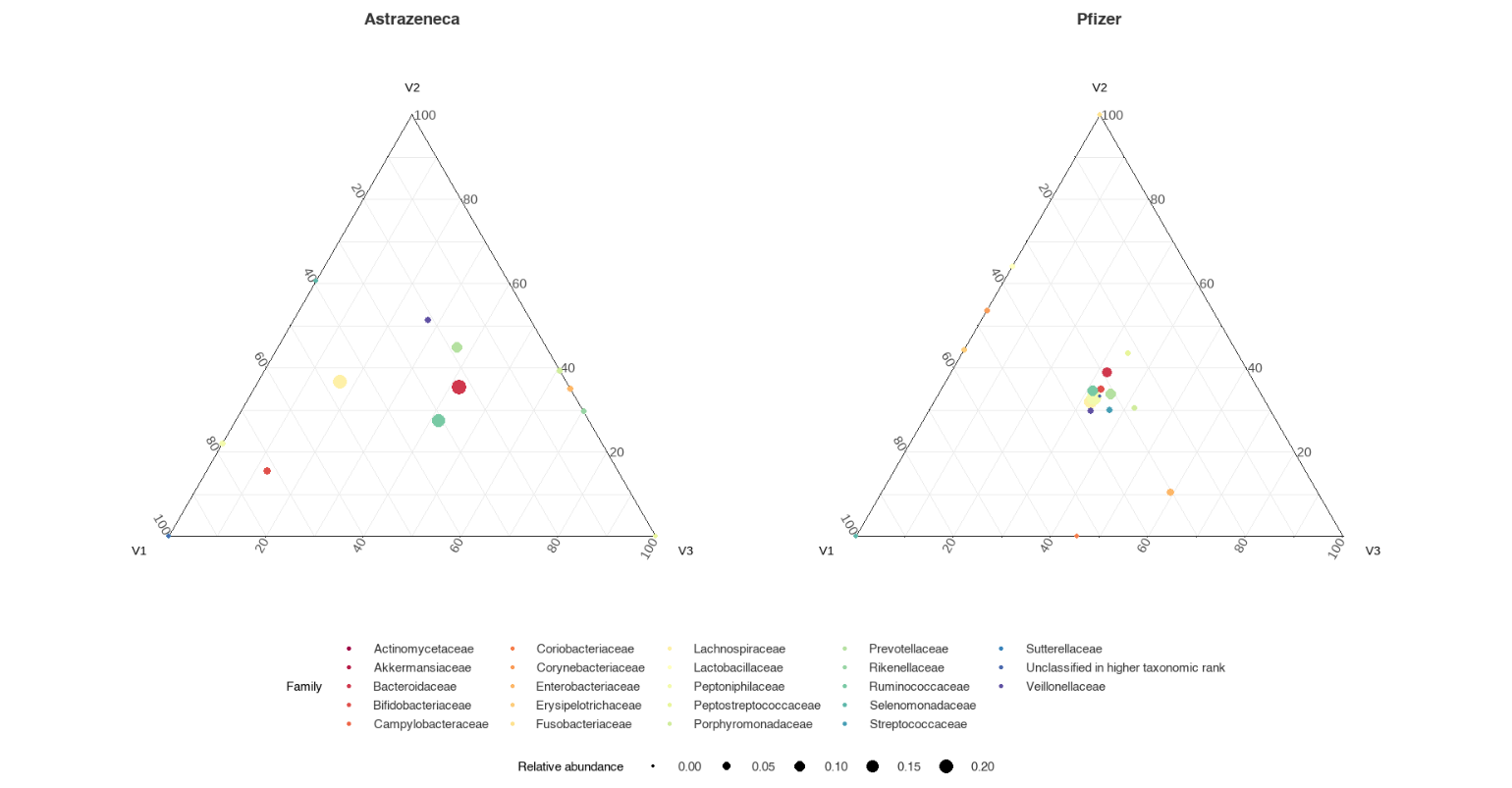

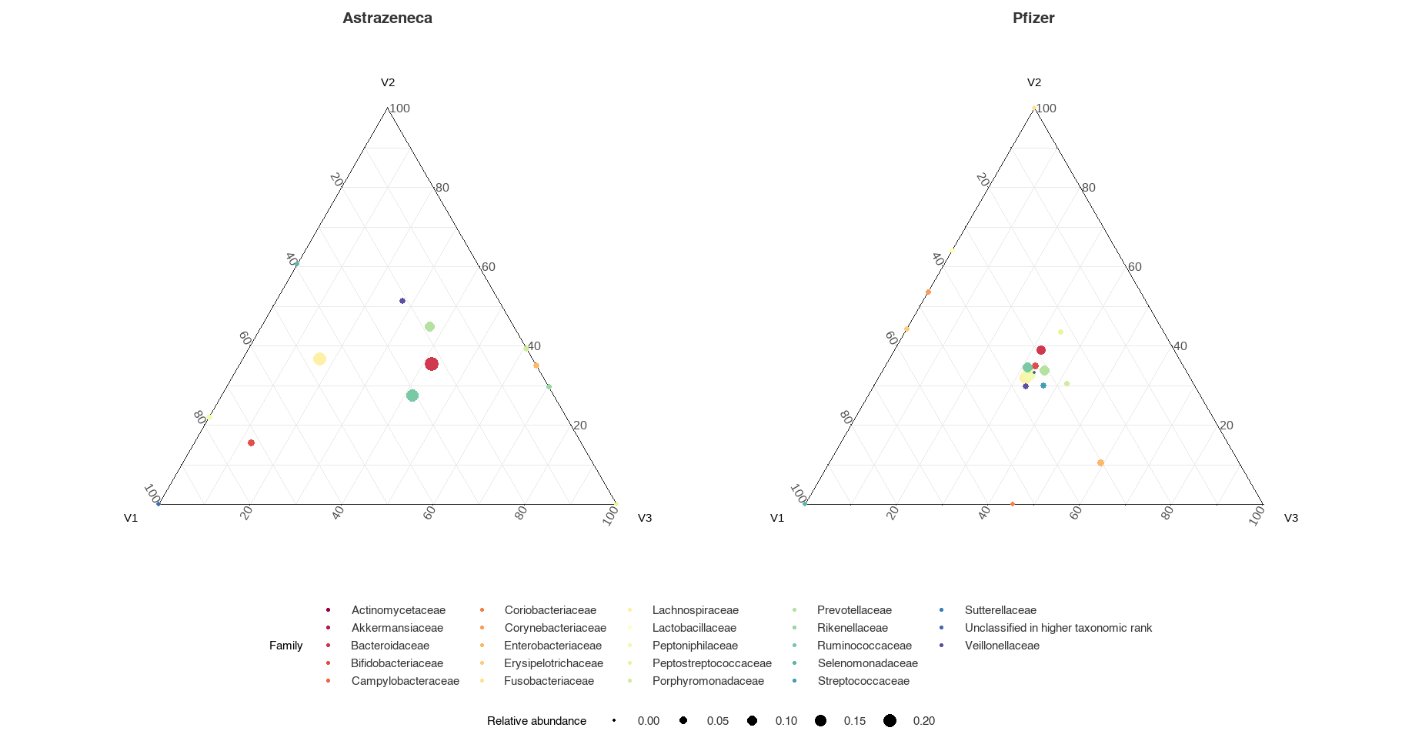

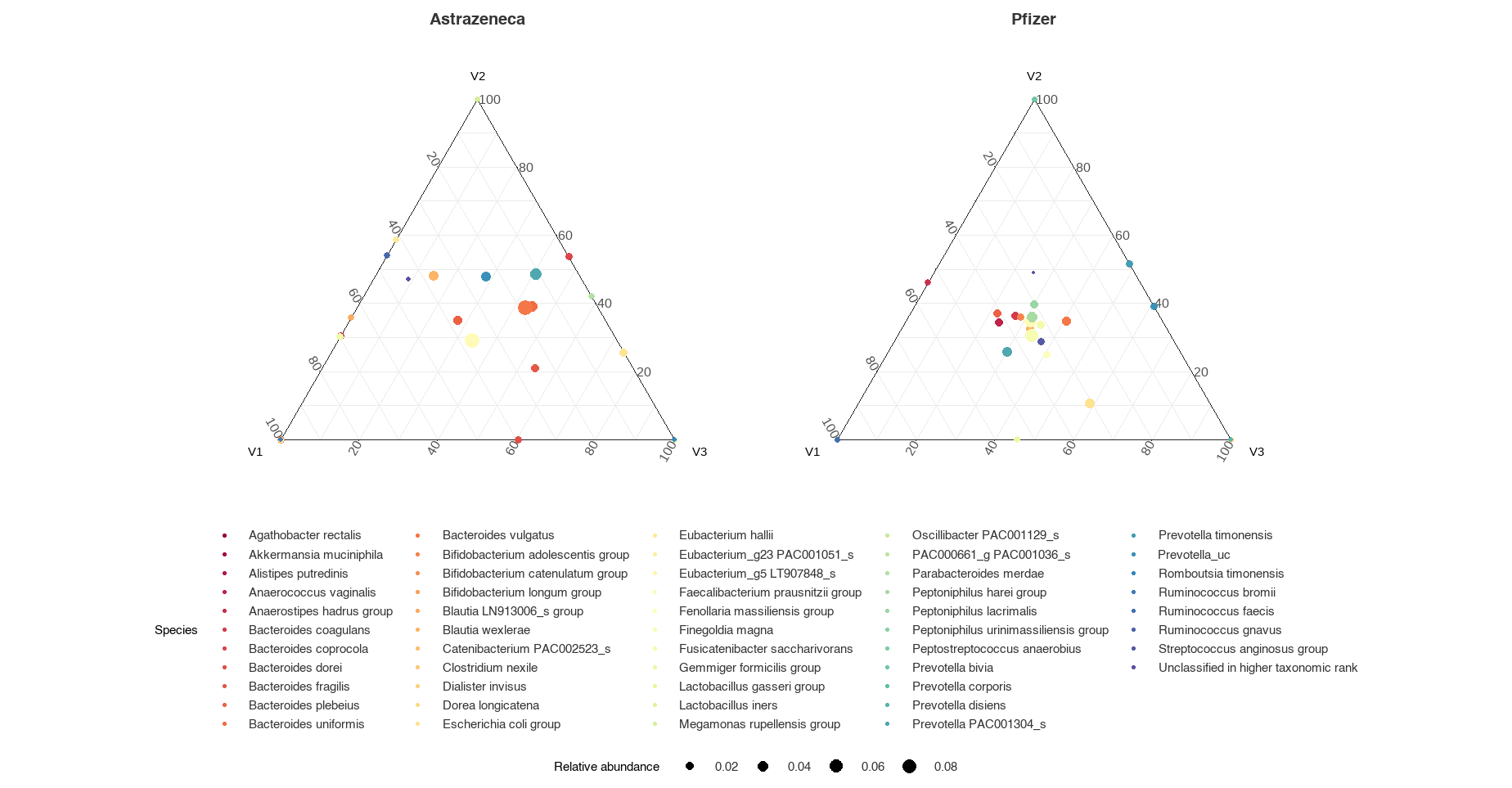

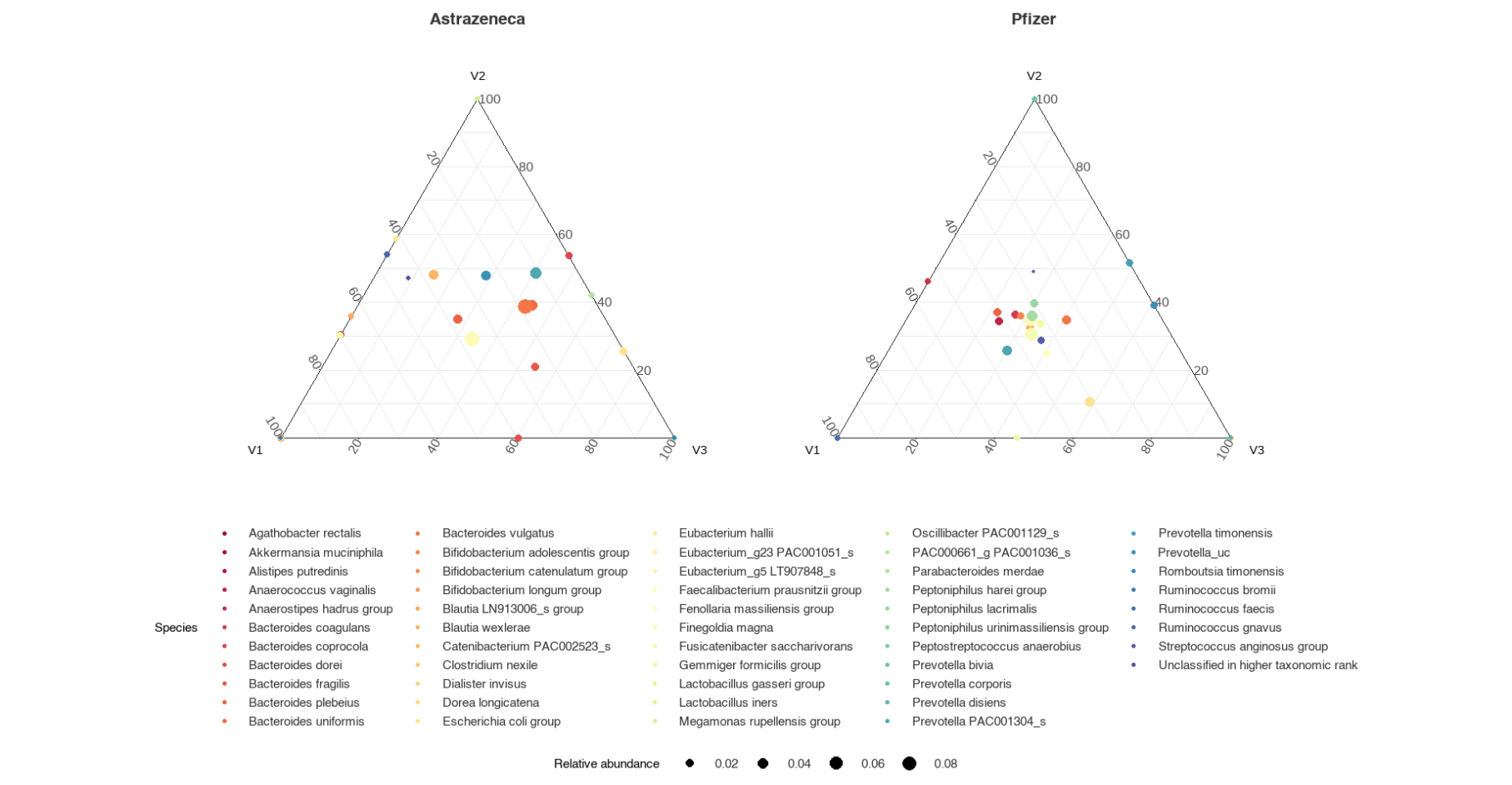

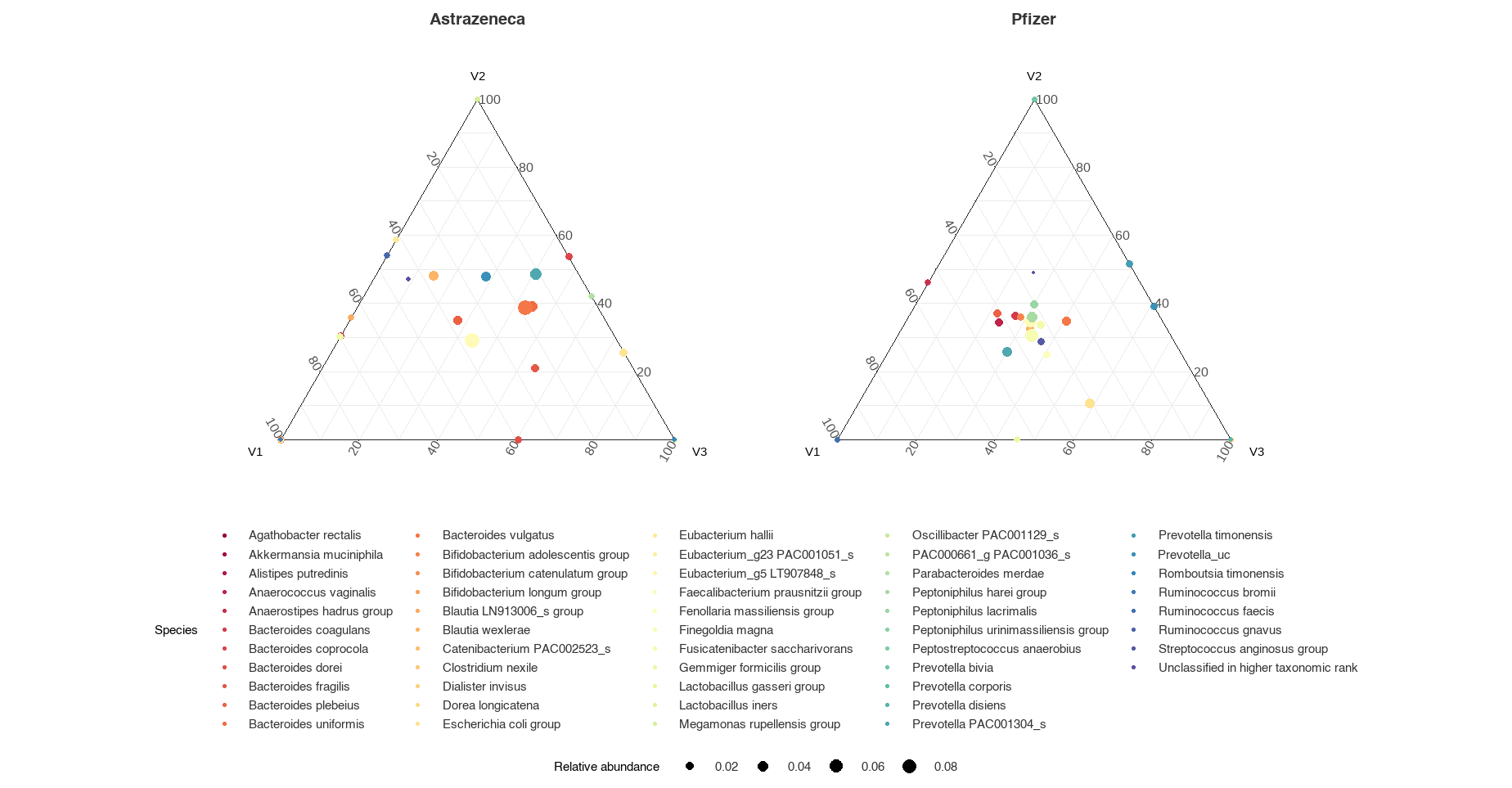


**BNT162b2**

**(e)**

**ChAdOx1**

**BNT162b2**

**(a) CHAO**

**ChAdOx1**

**(b) Jackknife**


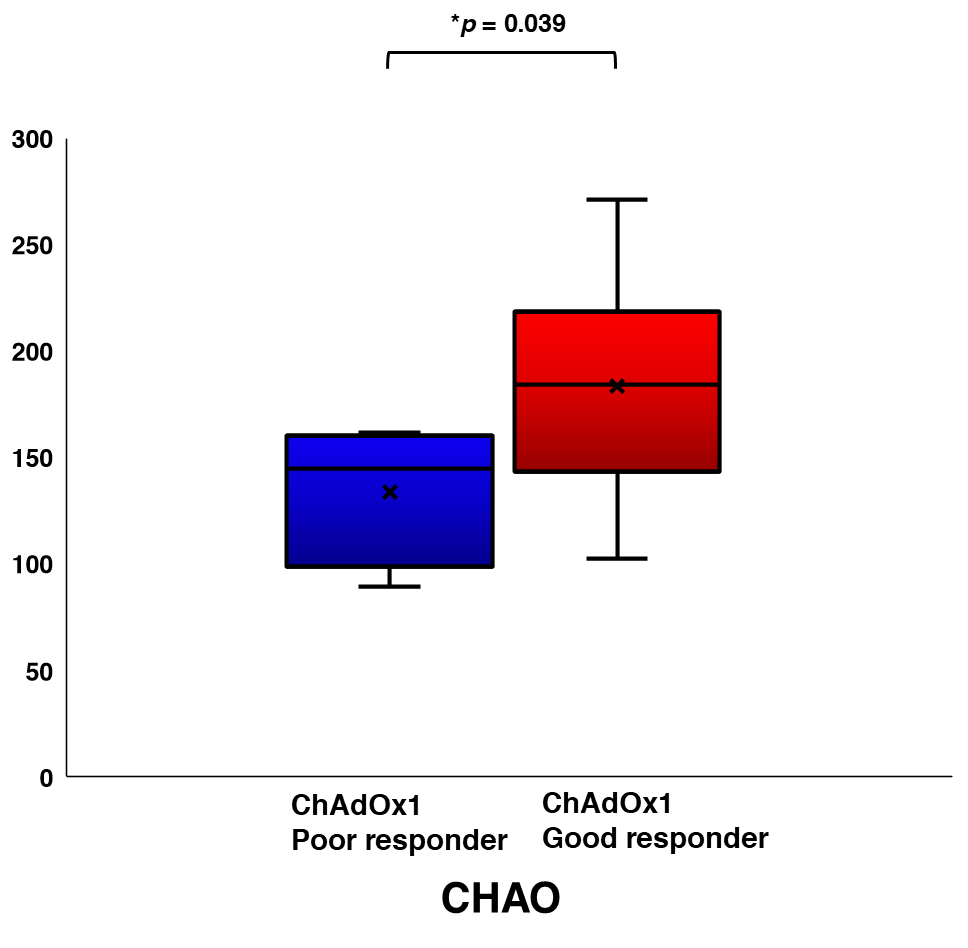

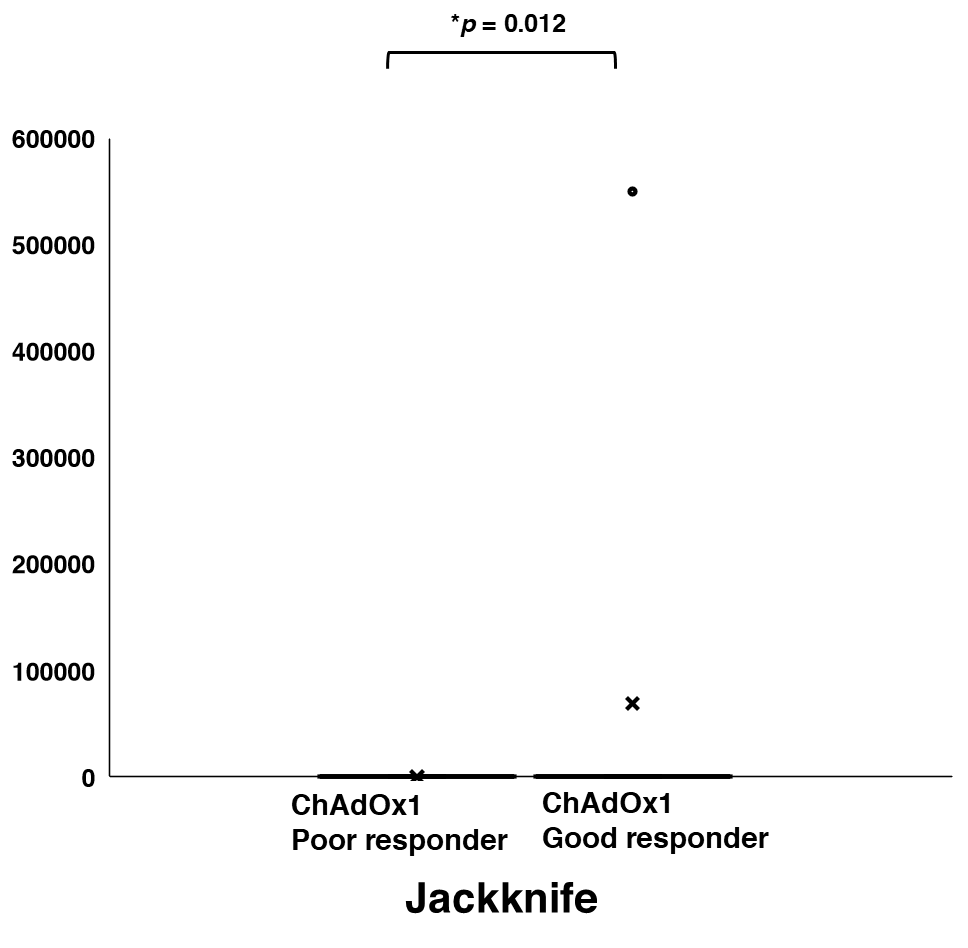

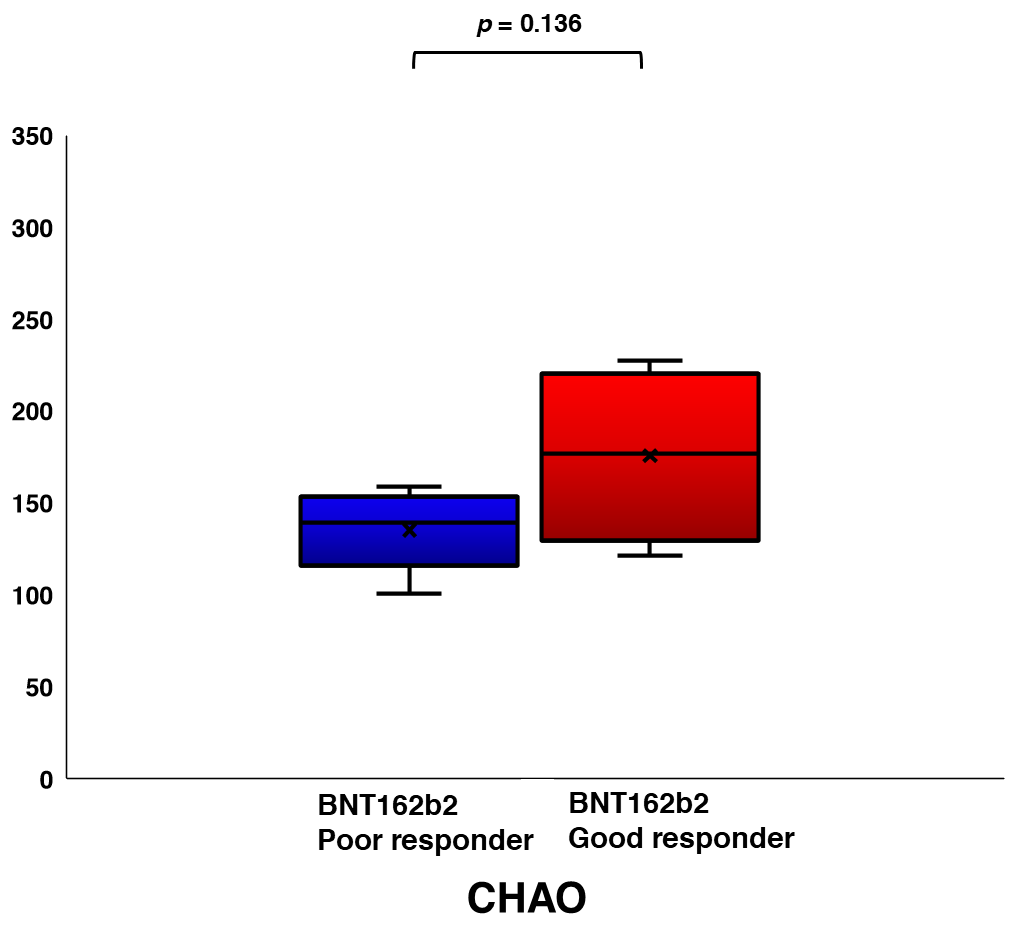

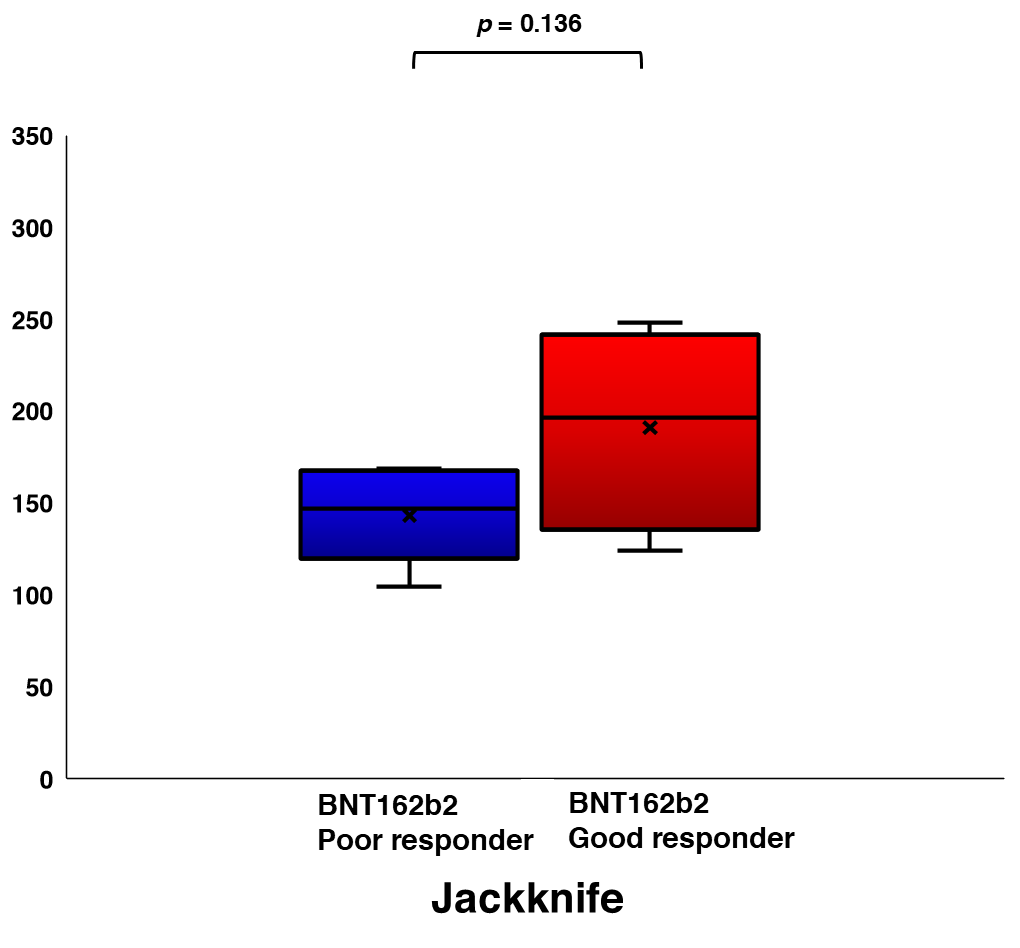


**Low responder**

**High responder**

**Low responder**

**High responder**

**Low responder**

**High responder**

**Low responder**

**High responder**

**Low responder**

**High responder**

**Low responder**

**High responder**

**BNT162b2**

**(c) NP Shannon**

**ChAdOx1**

**(d) Shannon**


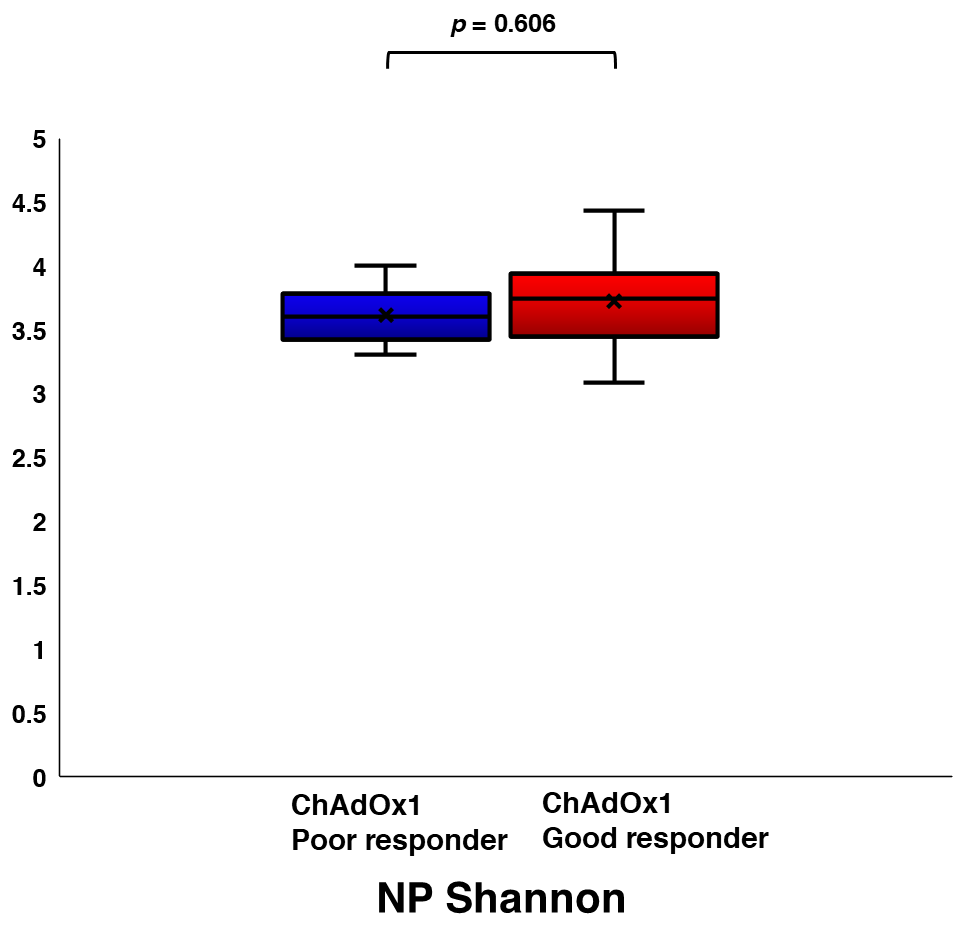

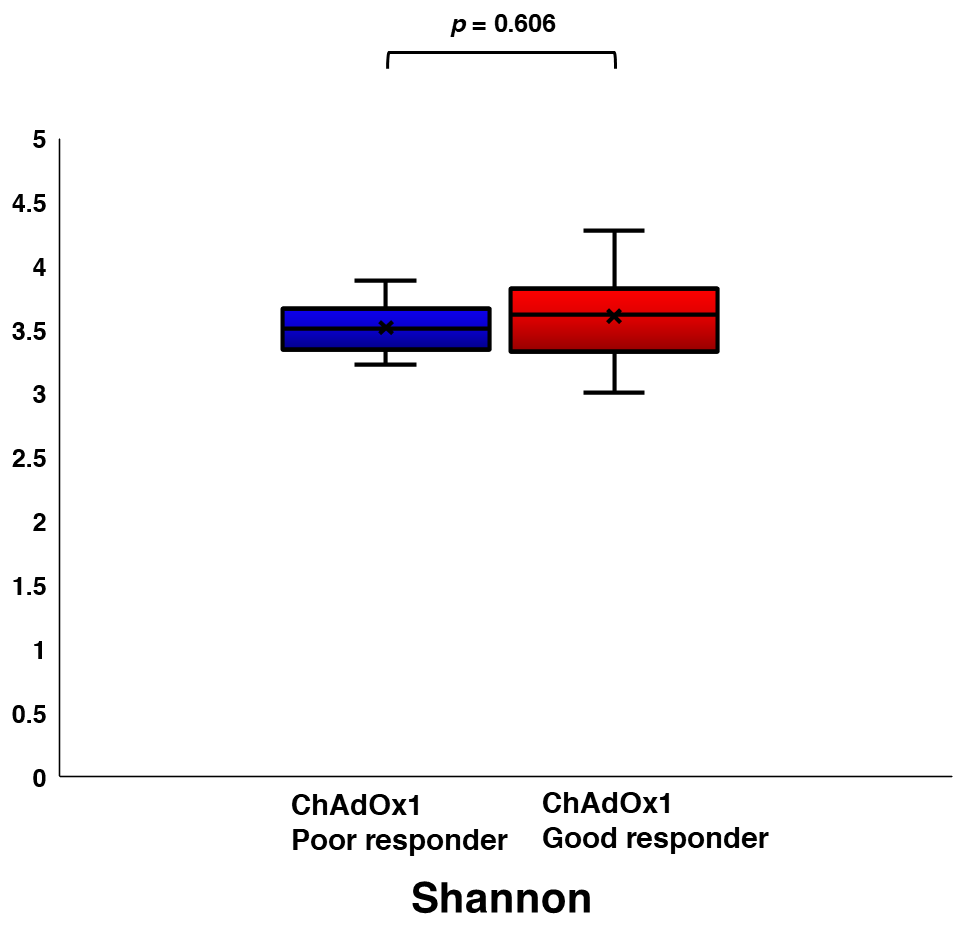

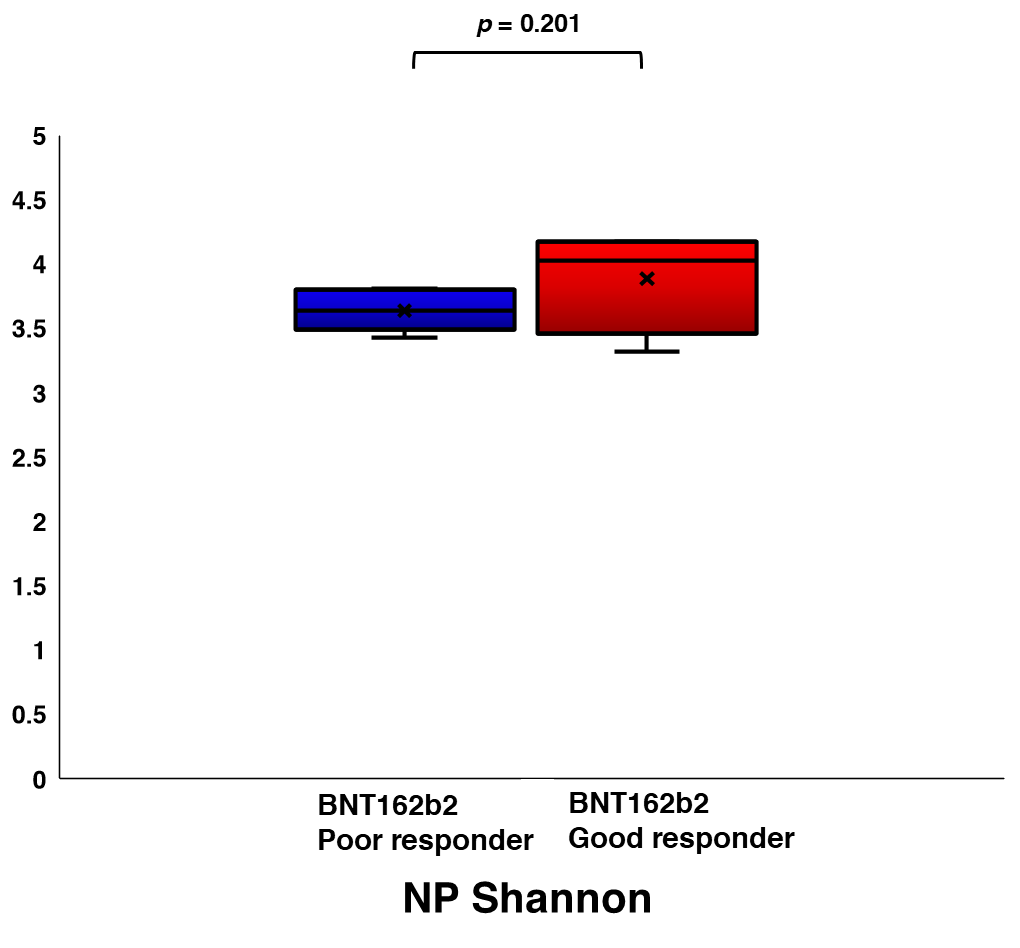

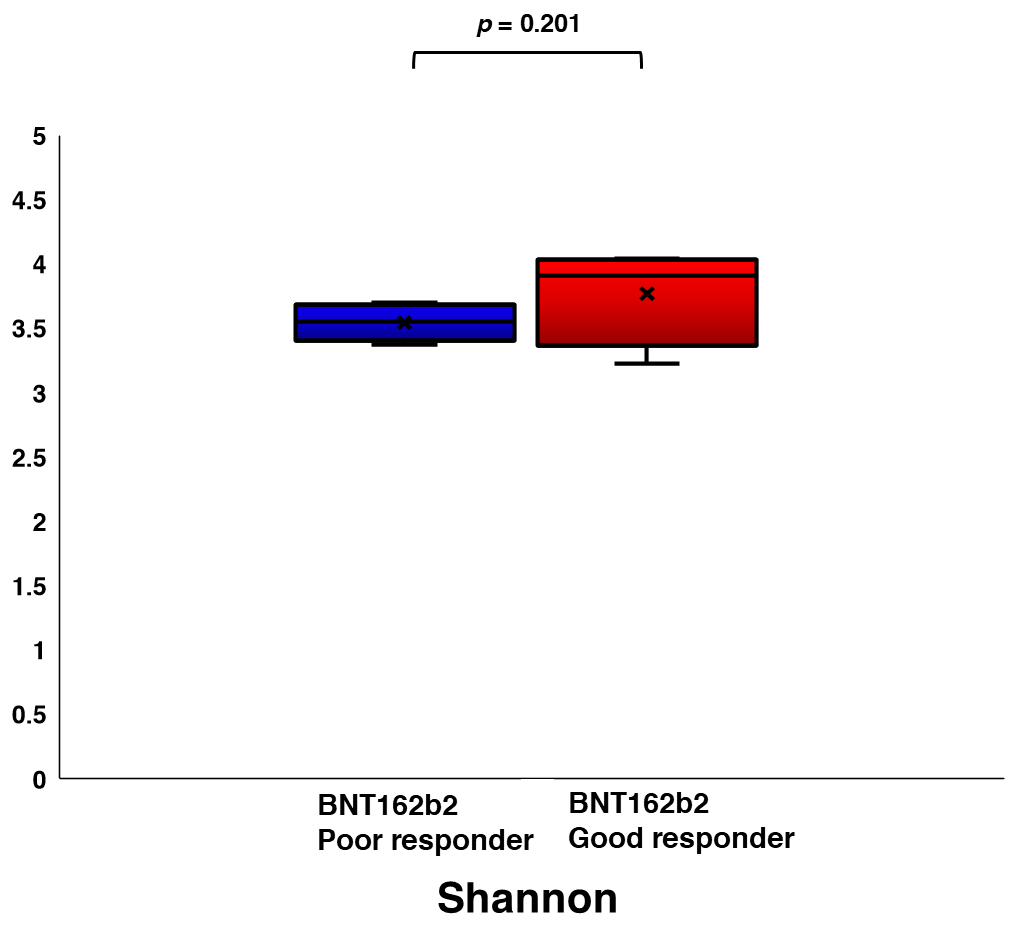


**Low responder**

**High responder**

**Low responder**

**High responder**

**Low responder**

**High responder**

**BNT162b2**

**(e) Simpson**

**ChAdOx1**

**(f) Phylogenetic Diversity**


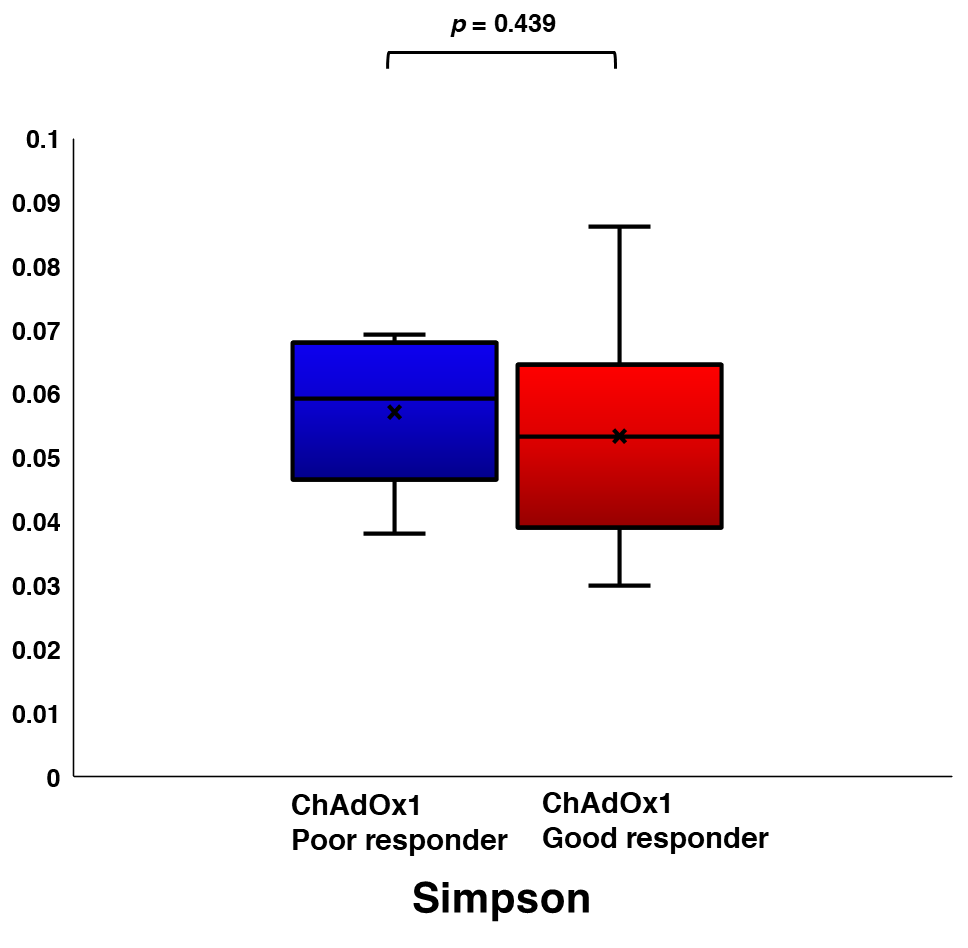

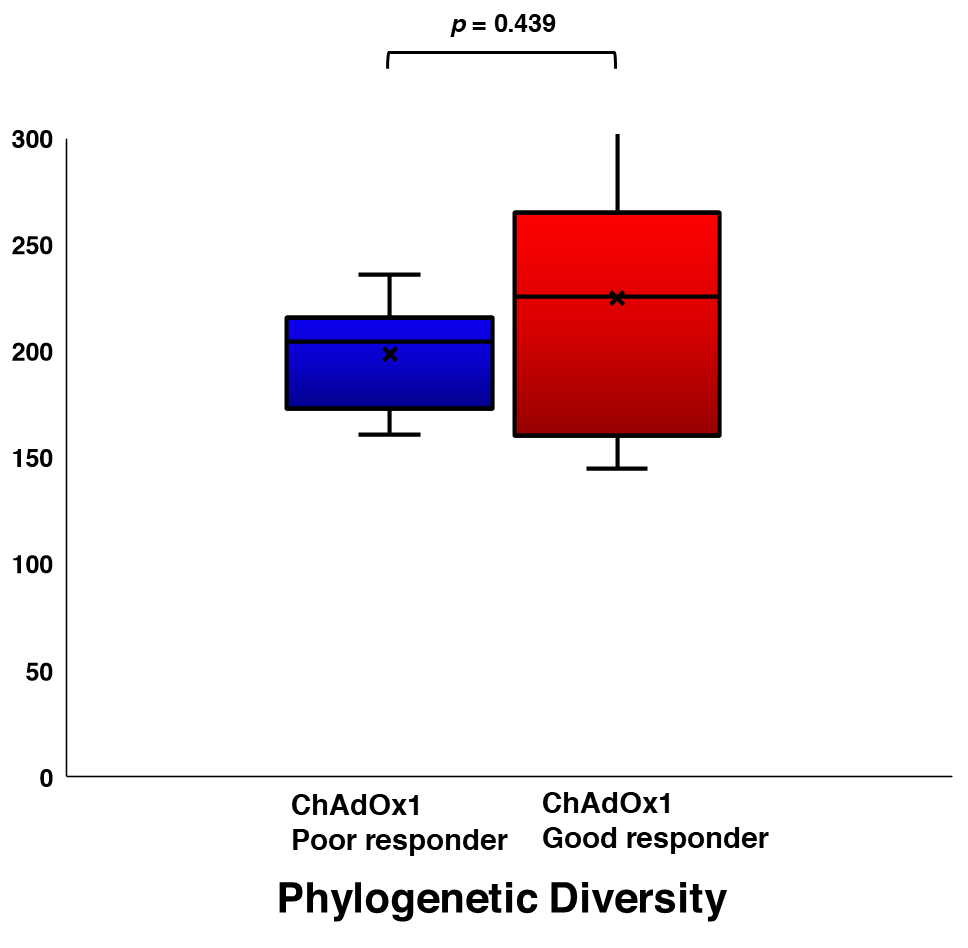

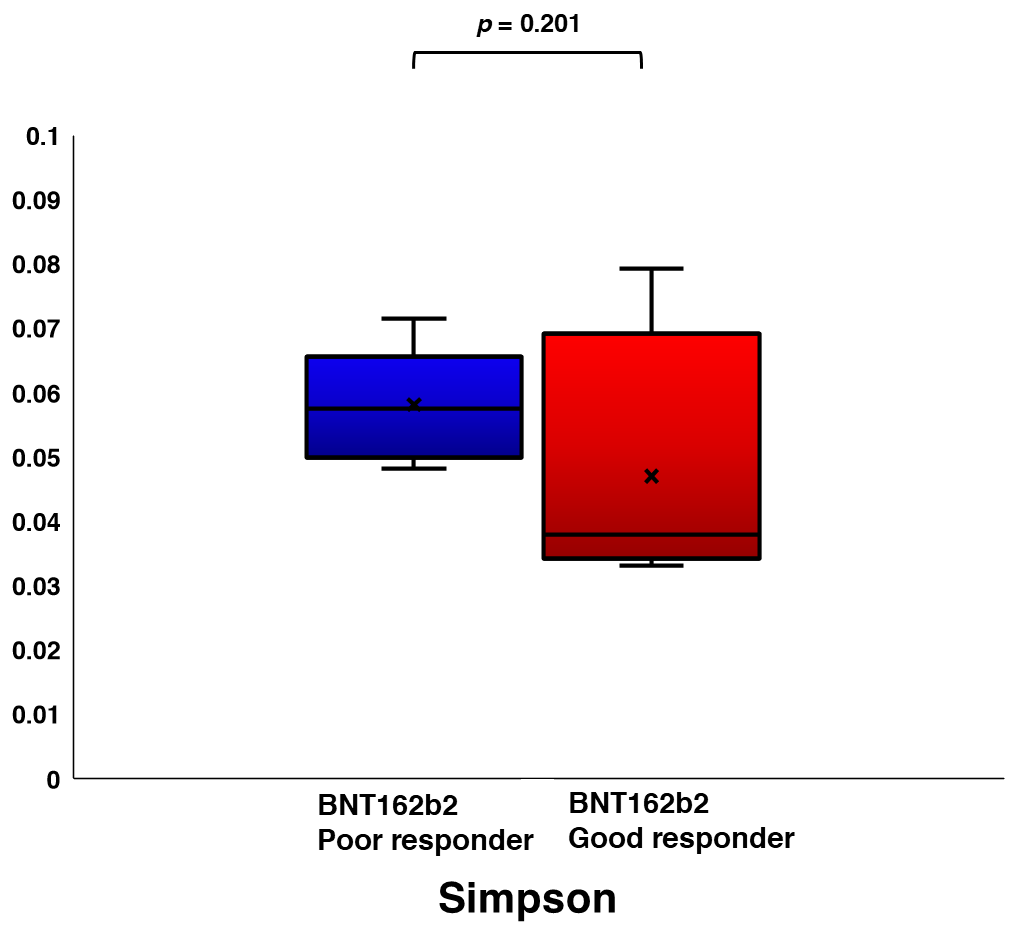

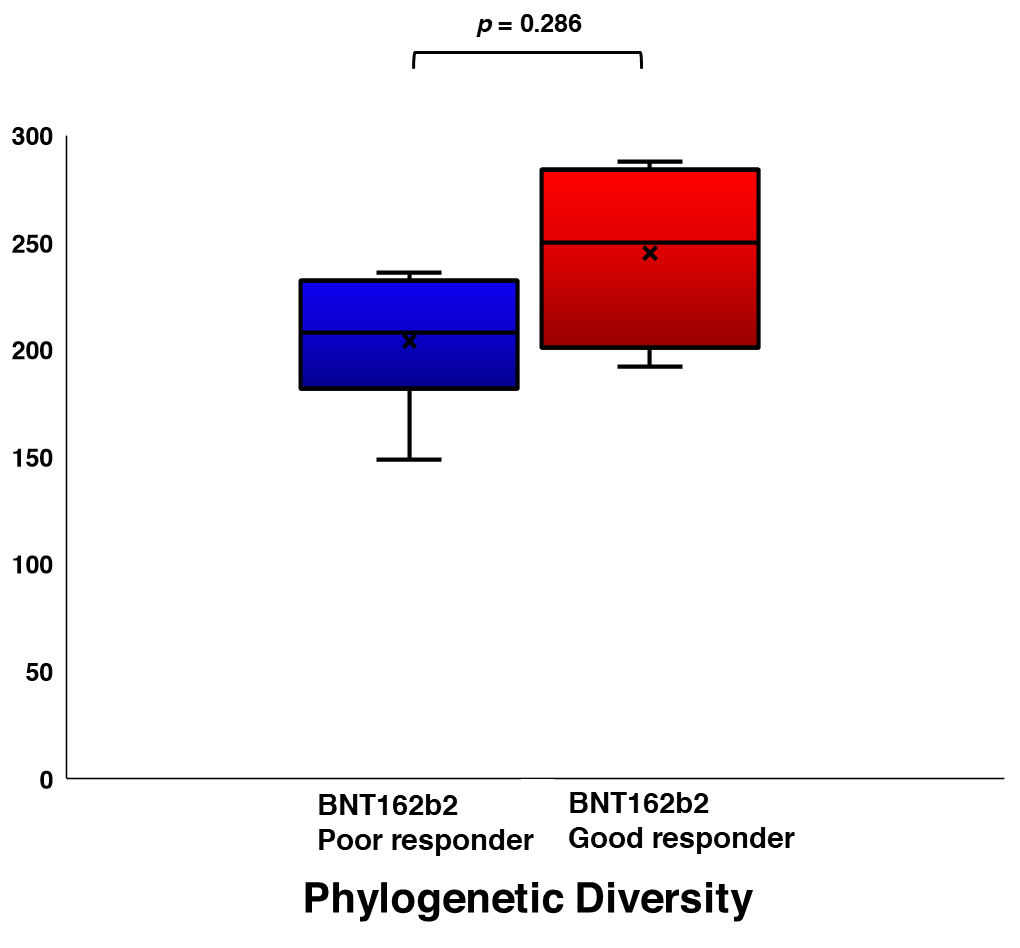


**Low responder**

**High responder**

**Low responder**

**High responder**

**Low responder**

**High responder**

**Figure S4.** **Linear discriminant analysis effect size (LEfSe) analysis to identify taxonomic biomarkers for immune response (high vs. low) in ChAdOx1 (a) and BNT162b2 (b) recipients. (a)** V2 taxonomic biomarkers based on the antibody titers of V3 and V1 taxonomic biomarkers based on the antibody titers of V2 in the ChAdOx1-vaccinated group. **(b)** V2 taxonomic biomarkers based on the antibody titers of V3 and V1 taxonomic biomarkers based on the antibody titers of V2 in the BNT162b2-vaccinated group. Only taxa with p<0.05 are presented.

**(a)**


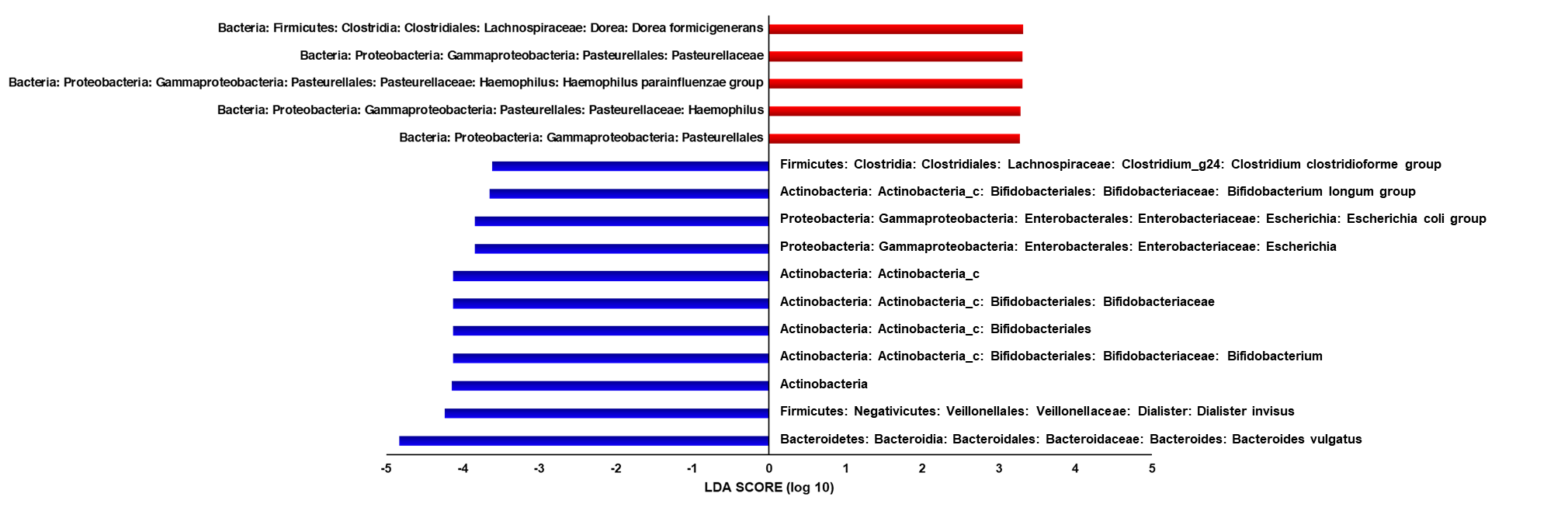

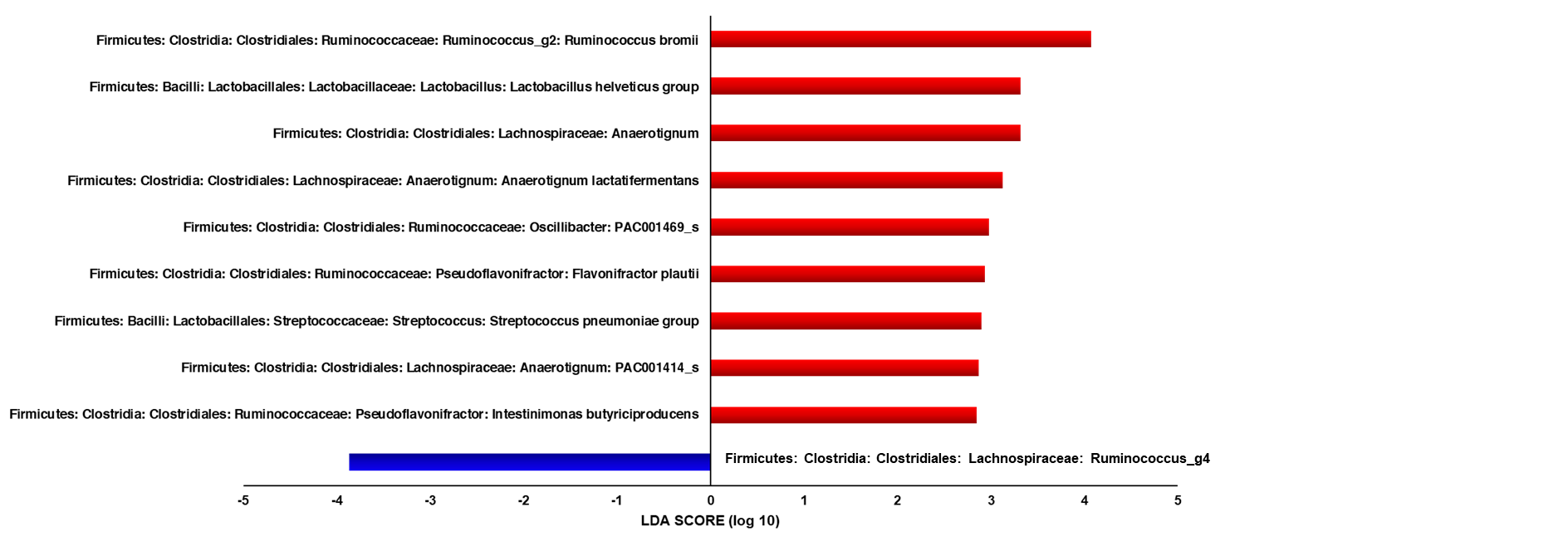


**ChAdOx1**

**V2 taxonomic markers**

**V1 taxonomic markers**

**(b)**

**BNT162b2**


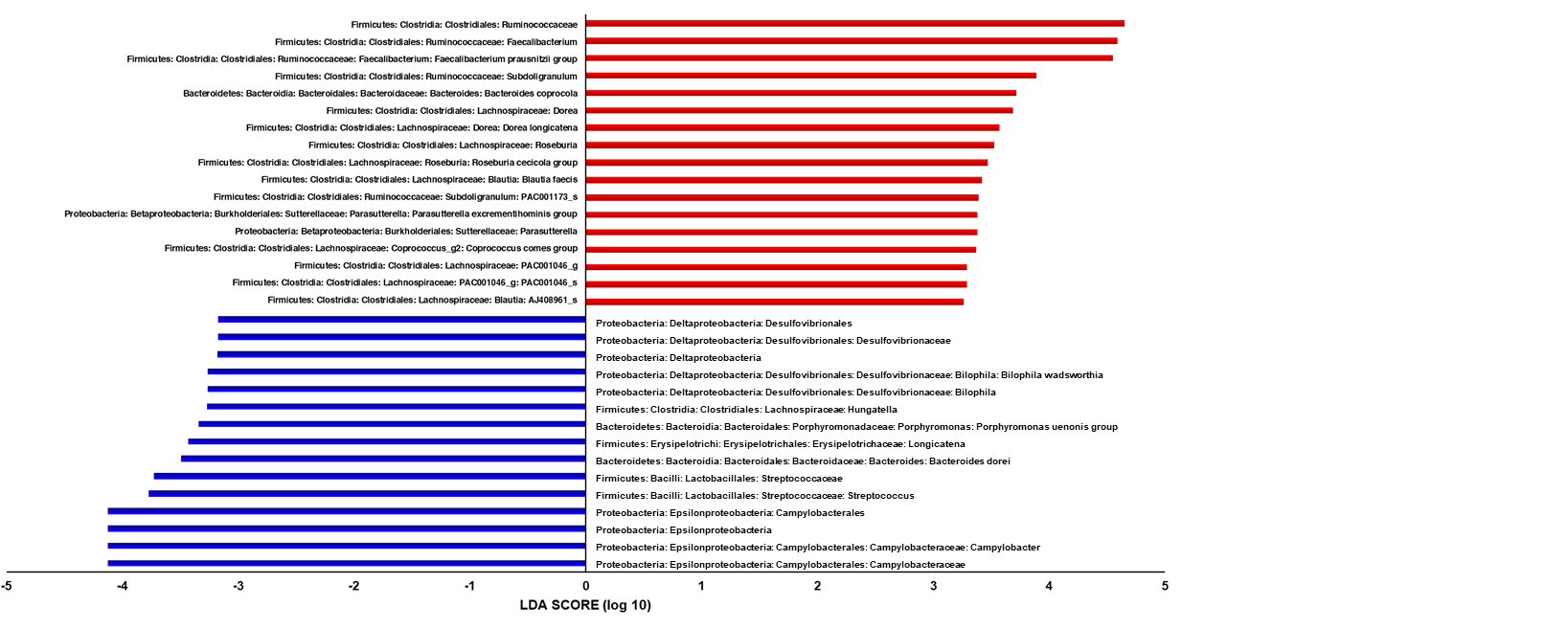

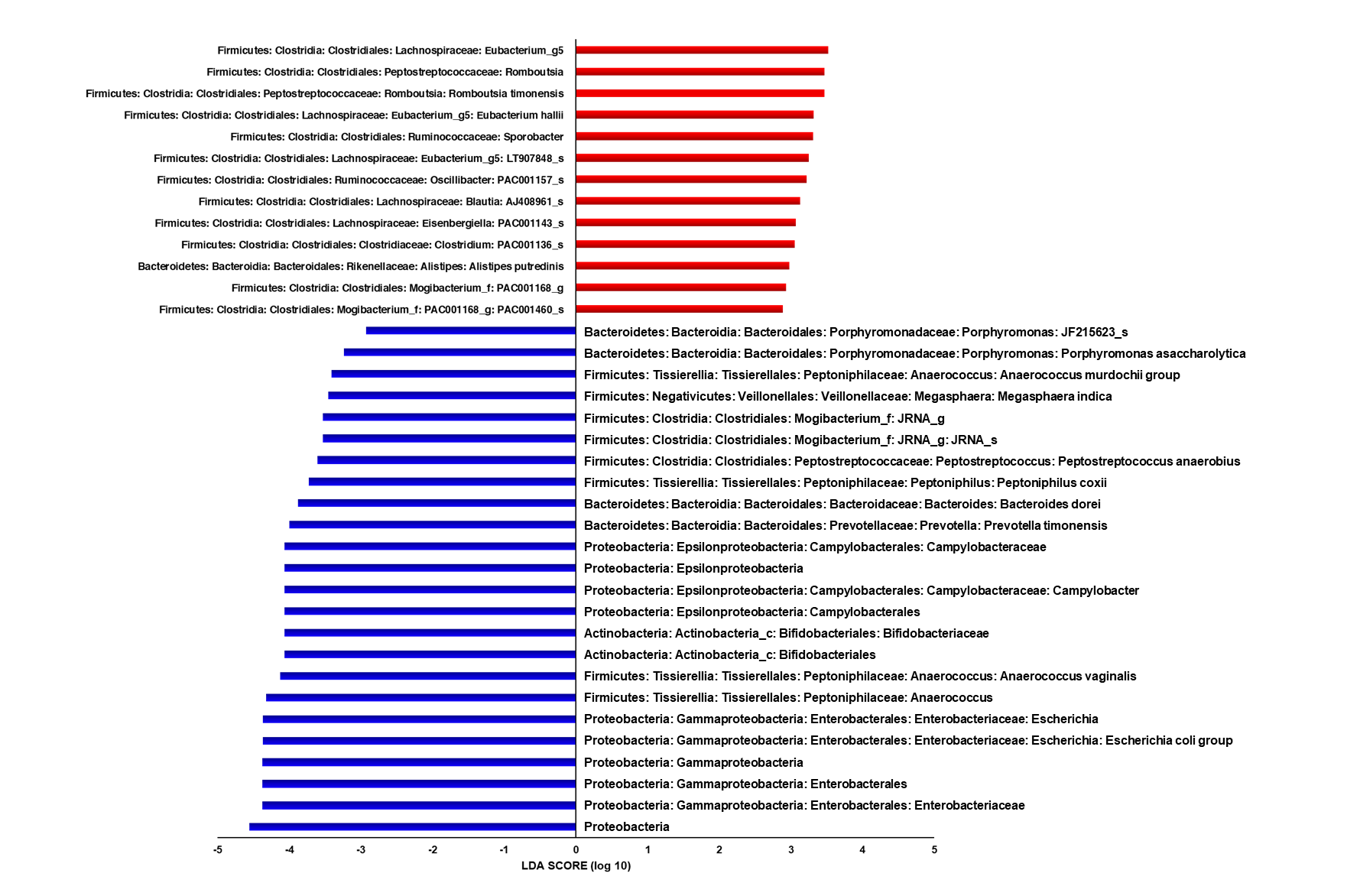


**V2 taxonomic markers**

**V1 taxonomic markers**

**Figure S5. Linear discriminant analysis effect size (LEfSe) analysis to identify functional biomarkers for immune response (high vs. low) in ChAdOx1 (a) and BNT162b2 (b) recipients.  (a)** V2 functional biomarkers based on the antibody titers of V3 and V1 functional biomarkers based on the antibody titers of V2 in the ChAdOx1-vaccinated group. **(b)** V2 functional biomarkers based on the antibody titers of V3 and V1 functional biomarkers based on the antibody titers of V2 in the BNT162b2-vaccinated group. Only taxa with p<0.05 are presented.

**(a) ChAdOx1**

**V2 functional markers**

**V1 functional markers**


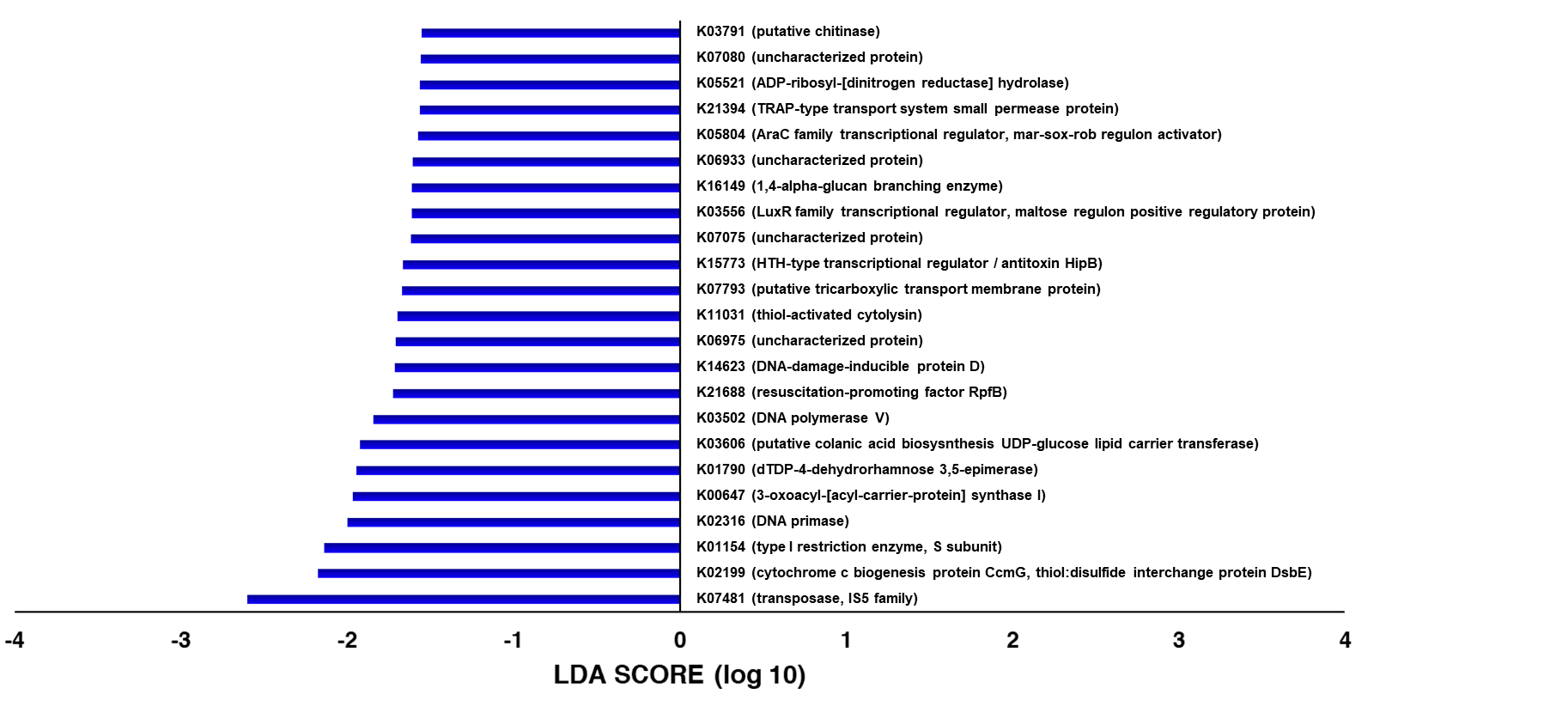

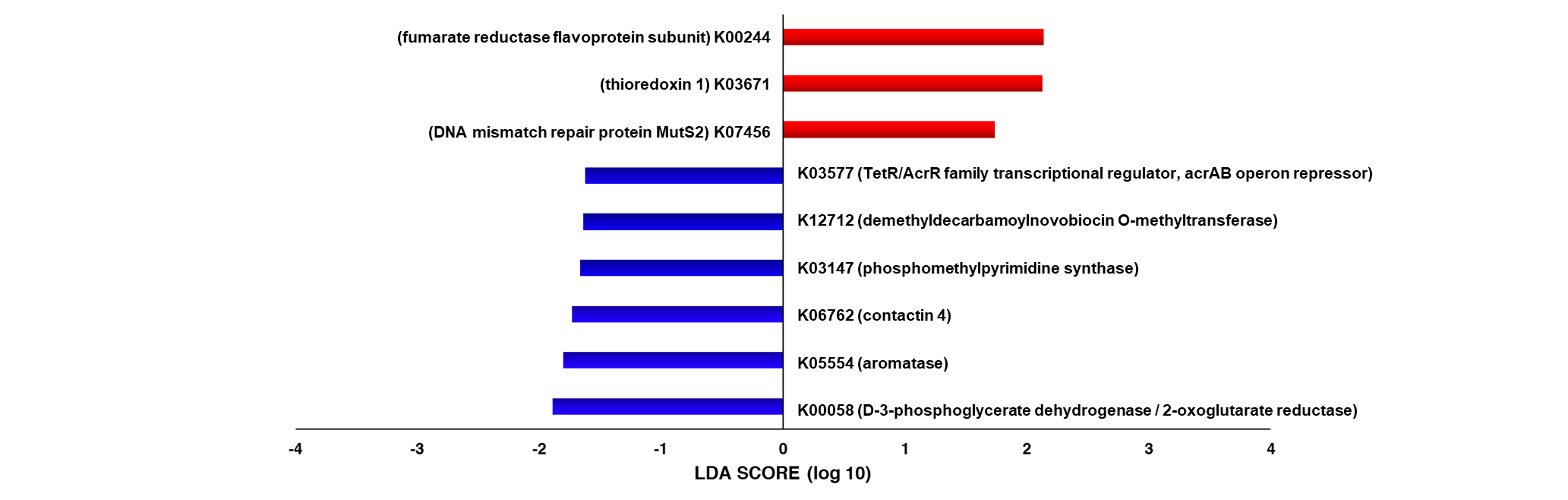


**(b) BNT162b2**

**V2 functional markers**

**V1 functional markers**


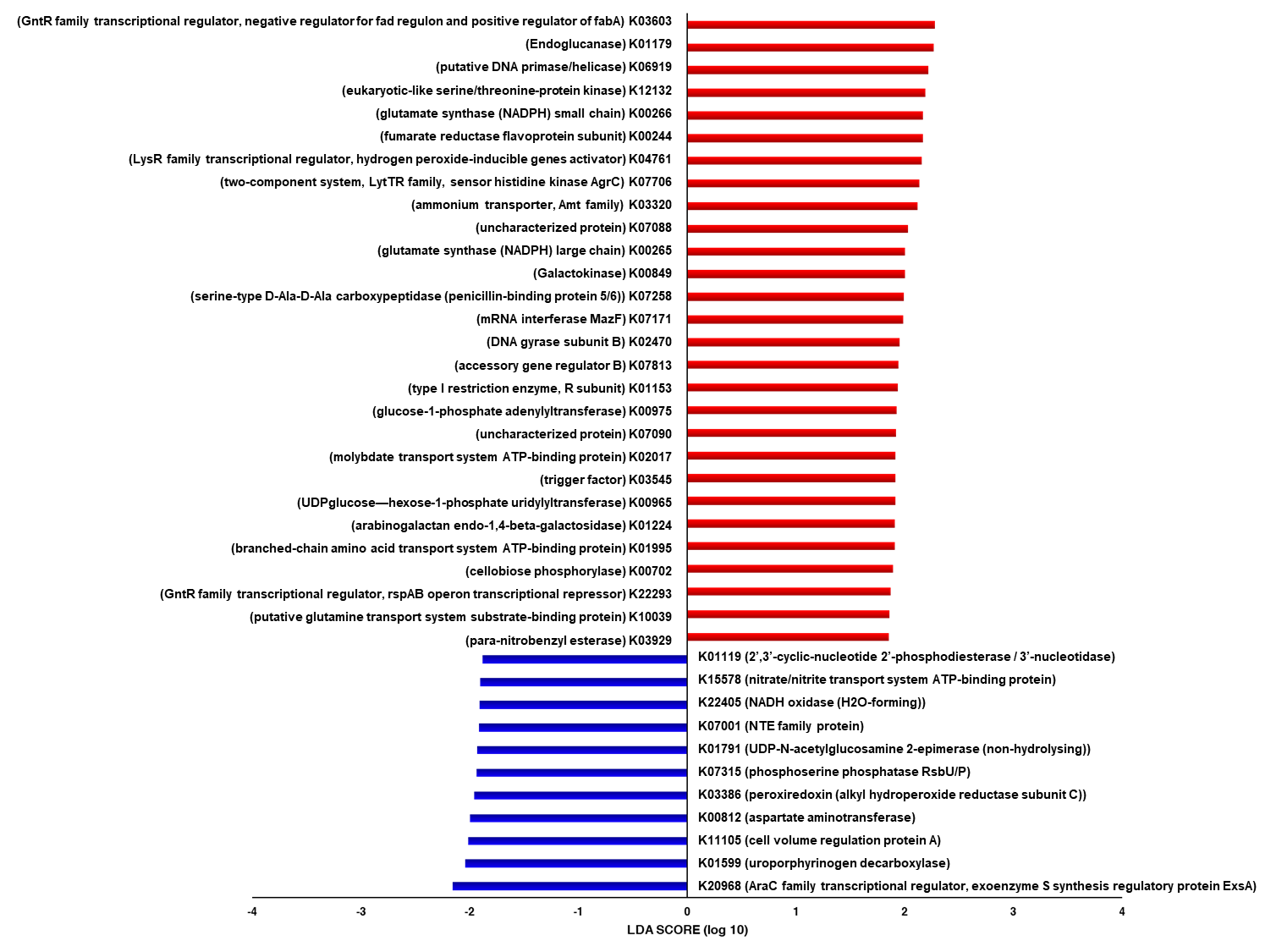

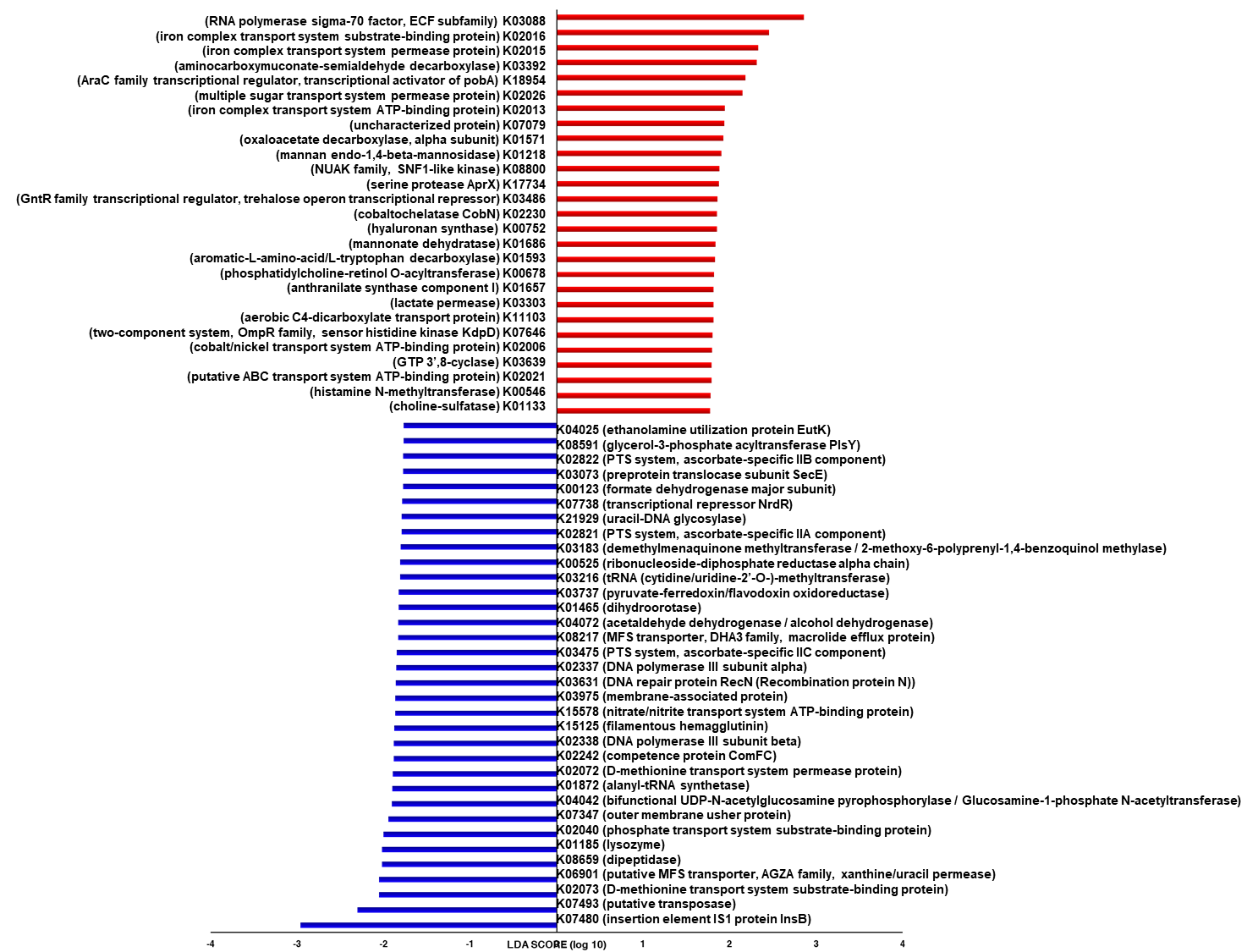


**Figure S6. Correlation between taxonomic biomarkers and 112 listed food items.** Spearman rank analysis was conducted to evaluate the association between immunogenicity-related taxonomic biomarkers and 112 listed food items in participants with ChAdOx1 (a) and BNT162b2 (b) vaccination, respectively. The color gradients indicate the degree of correlation from red (positive correlation) to blue (negative correlation). *p < 0.05; **p = 0.01 – 0.001; ***p < 0.001.

**
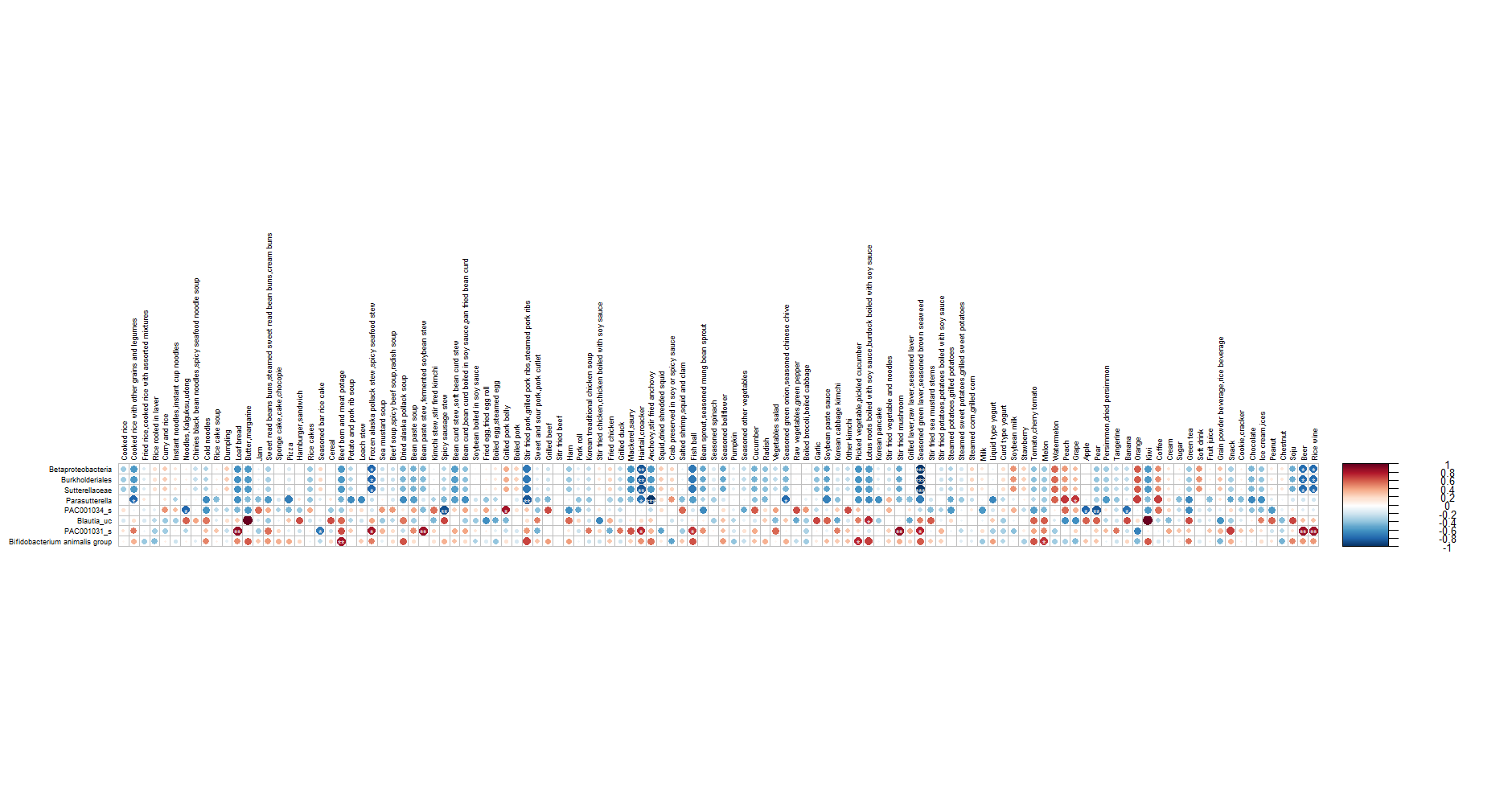

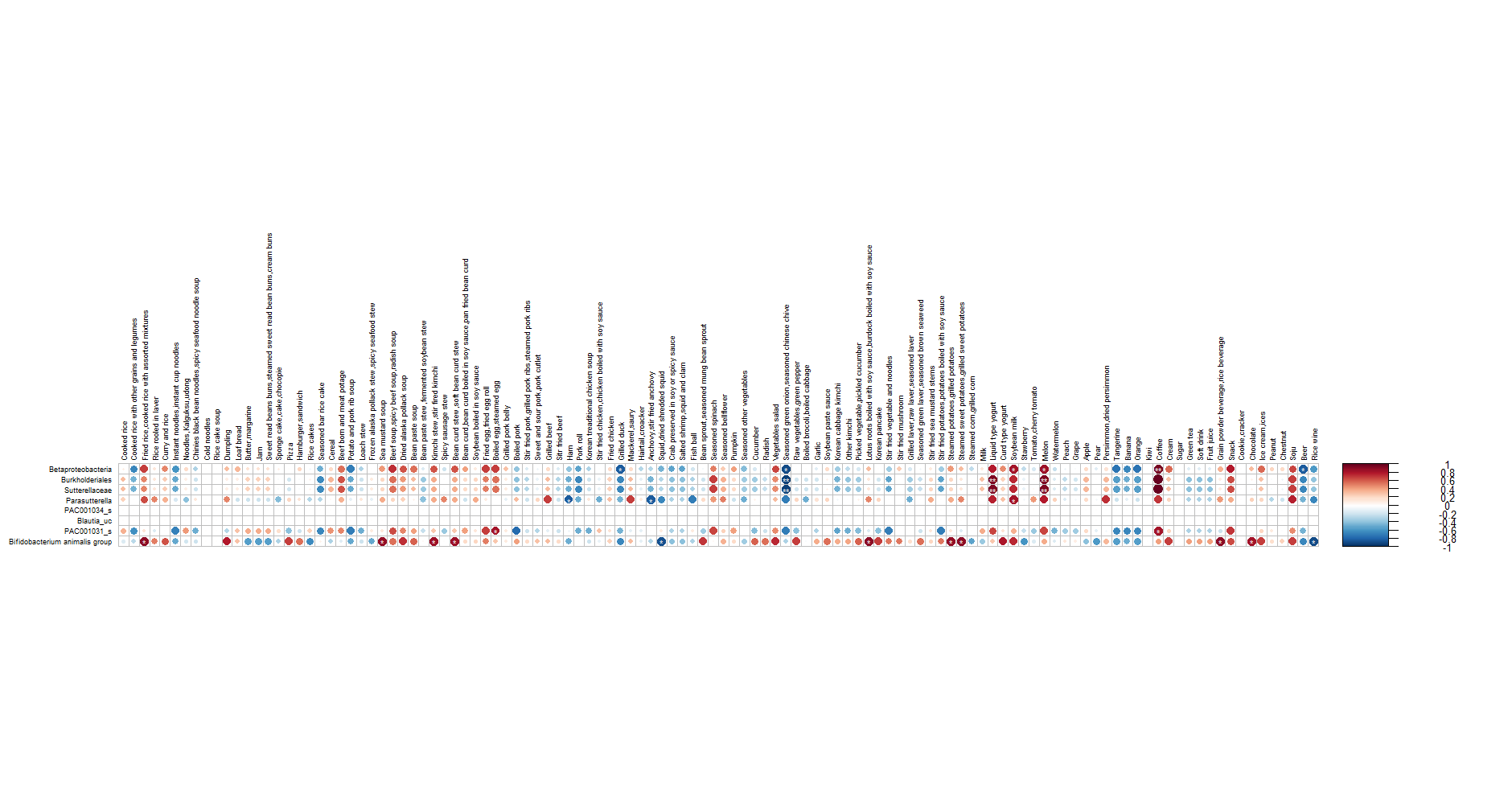
**

**(a) ChAdOx1**

**Low responders**

**High responders**

**
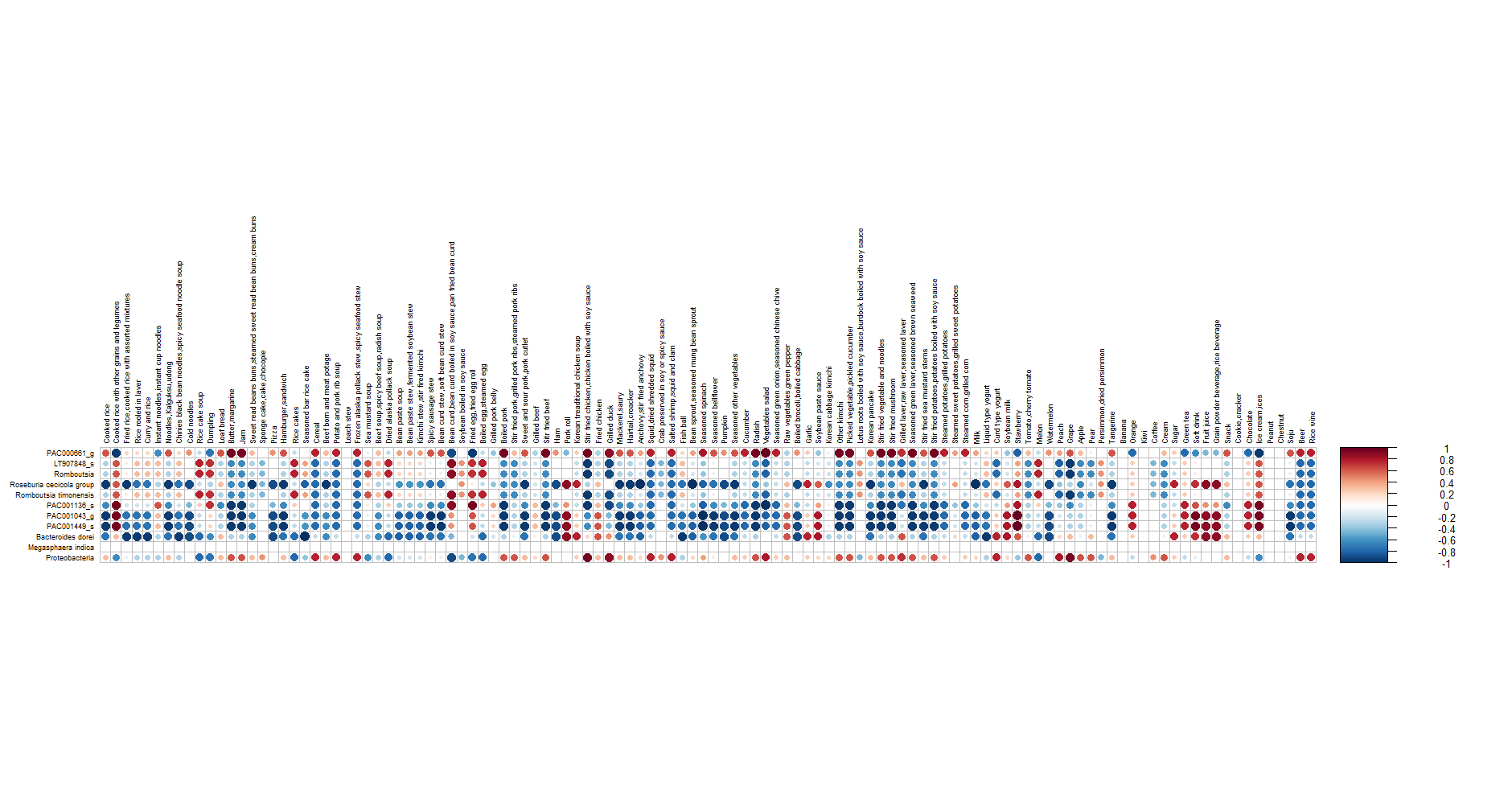
**

**(b) BNT162b2**

**
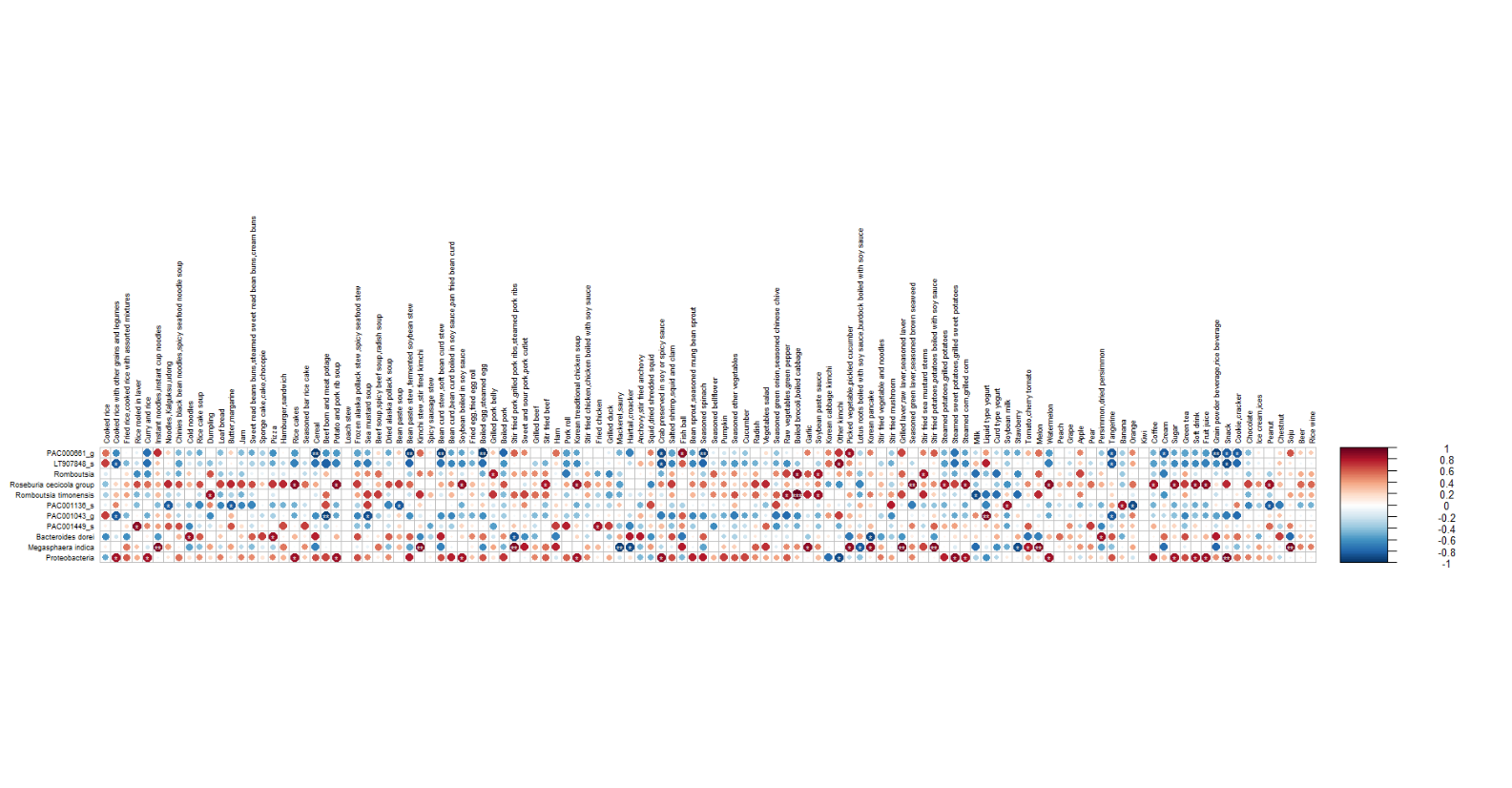
**

**Low responders**

**High responders**

**Figure S7. Correlation analysis between taxonomic biomarkers and daily energy and 13 nutrients.**Nutrients calculated based on food frequency questionnaires and taxonomic biomarkers correlated in the ChAdOx1- (**a**) and BNT162b2- (**b**) vaccinated groups.

**(a) ChAdOx1**

**High responders**

**Low responders**


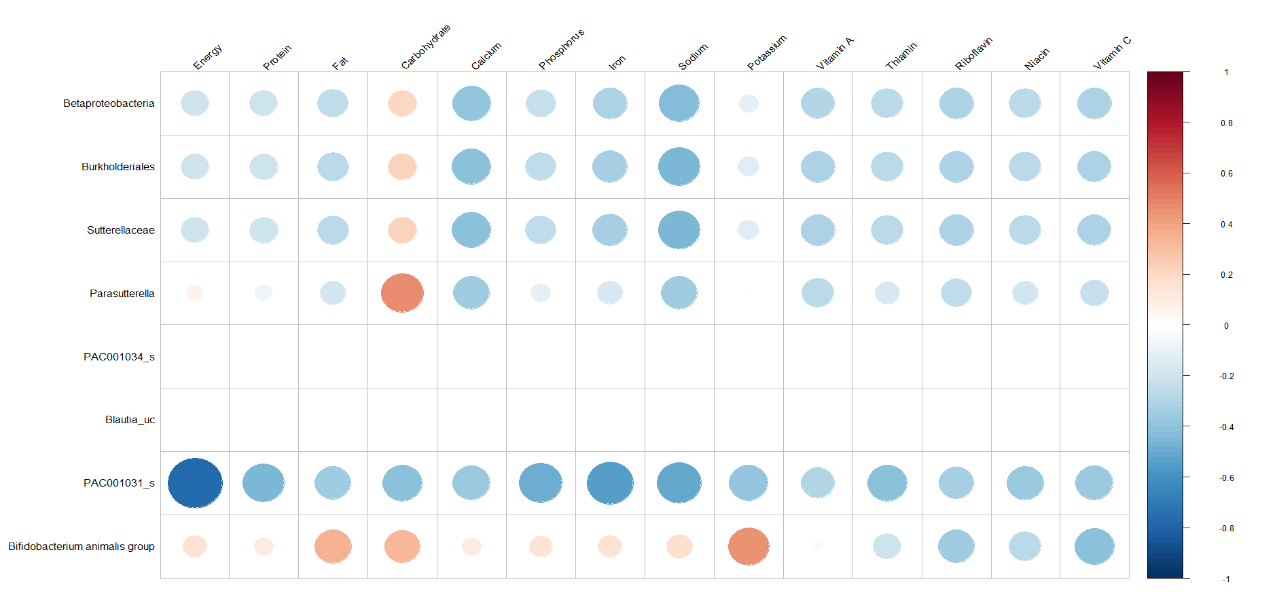

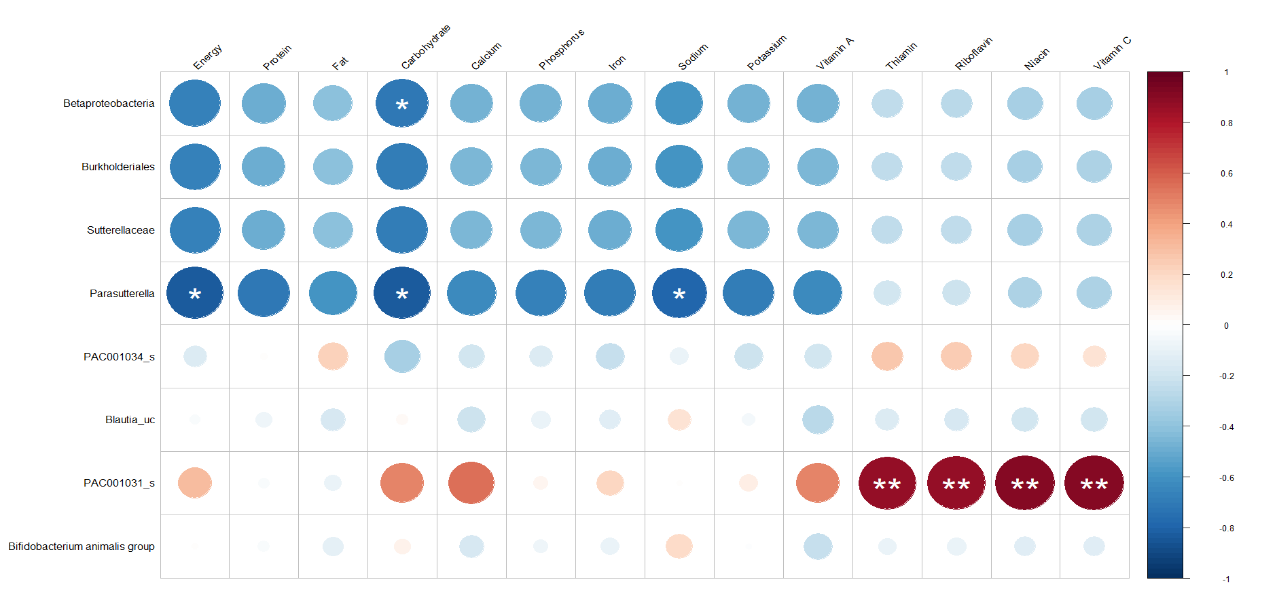


**(b) BNT162b2**

**High responders**

**Low responders**


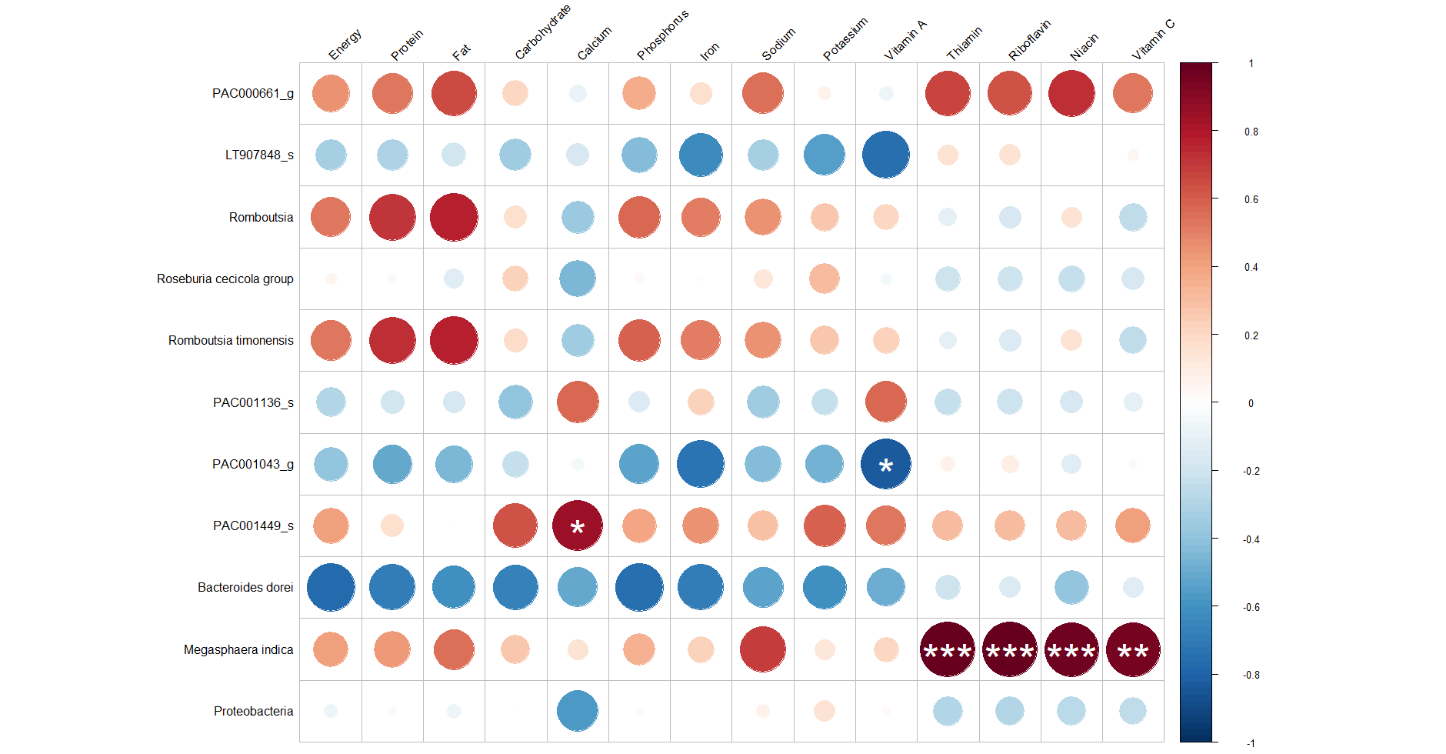

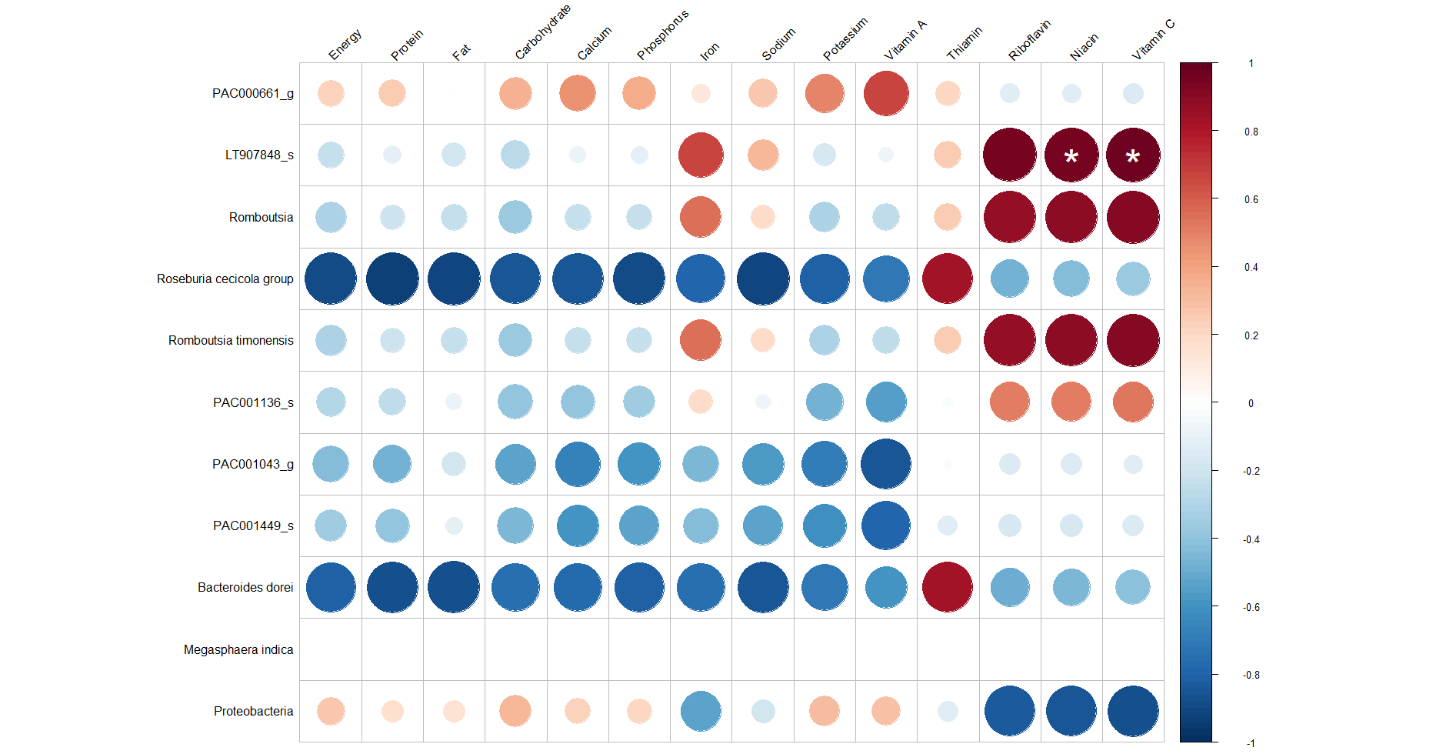

Supplement: Supplementary file 1 — Supplementary information [file 41392_2023_1445_MOESM1_ESM.docx]
